# Genomic and Phenotypic Variation in Morphogenetic Networks of Two *Candida albicans* Isolates Subtends Their Different Pathogenic Potential

**DOI:** 10.3389/fimmu.2017.01997

**Published:** 2018-01-19

**Authors:** Duccio Cavalieri, Monica Di Paola, Lisa Rizzetto, Noemi Tocci, Carlotta De Filippo, Paolo Lionetti, Andrea Ardizzoni, Bruna Colombari, Simona Paulone, Ivo G. Gut, Luisa Berná, Marta Gut, Julie Blanc, Misha Kapushesky, Eva Pericolini, Elisabetta Blasi, Samuele Peppoloni

**Affiliations:** ^1^Dipartimento di Biologia, Università di Firenze, Florence, Italy; ^2^Dipartimento di Neuroscienze, Psicologia, Area del Farmaco e Salute del Bambino, Università di Firenze, Florence, Italy; ^3^Centro Ricerca e Innovazione, Fondazione Edmund Mach, San Michele all’Adige, Italy; ^4^Institute of Agricultural Biology and Biotechnology, National Research Council (CNR), Pisa, Italy; ^5^Dipartimento di Medicina Diagnostica, Clinica e di Sanità Pubblica, Università di Modena e Reggio Emilia, Modena, Italy; ^6^Centro Nacional de Anàlisi Genòmica, Barcelona, Spain; ^7^Unidad de Biologia Molecular, Institut Pasteur de Montevideo, Montevideo, Uruguay; ^8^European Bioinformatics Institute (EMBL-EBI), Cambridge, United Kingdom

**Keywords:** *Candida albicans*, host adaptation, biofilm, pathogenic traits, genomic, fungal isolates, phagocytes

## Abstract

The transition from commensalism to pathogenicity of *Candida albicans* reflects both the host inability to mount specific immune responses and the microorganism’s dimorphic switch efficiency. In this study, we used whole genome sequencing and microarray analysis to investigate the genomic determinants of the phenotypic changes observed in two *C. albicans* clinical isolates (YL1 and YQ2). *In vitro* experiments employing epithelial, microglial, and peripheral blood mononuclear cells were thus used to evaluate *C. albicans* isolates interaction with first line host defenses, measuring adhesion, susceptibility to phagocytosis, and induction of secretory responses. Moreover, a murine model of peritoneal infection was used to compare the *in vivo* pathogenic potential of the two isolates. Genome sequence and gene expression analysis of *C. albicans* YL1 and YQ2 showed significant changes in cellular pathways involved in environmental stress response, adhesion, filamentous growth, invasiveness, and dimorphic transition. This was in accordance with the observed marked phenotypic differences in biofilm production, dimorphic switch efficiency, cell adhesion, invasion, and survival to phagocyte-mediated host defenses. The mutations in key regulators of the hyphal growth pathway in the more virulent strain corresponded to an overall greater number of budding yeast cells released. Compared to YQ2, YL1 consistently showed enhanced pathogenic potential, since *in vitro*, it was less susceptible to ingestion by phagocytic cells and more efficient in invading epithelial cells, while *in vivo* YL1 was more effective than YQ2 in recruiting inflammatory cells, eliciting IL-1β response and eluding phagocytic cells. Overall, these results indicate an unexpected isolate-specific variation in pathways important for host invasion and colonization, showing how the genetic background of *C. albicans* may greatly affect its behavior both *in vitro* and *in vivo*. Based on this approach, we propose that the co-occurrence of changes in sequence and expression in genes and pathways driving dimorphic transition and pathogenicity reflects a selective balance between traits favoring dissemination of the pathogen and traits involved in host defense evasion. This study highlights the importance of investigating strain-level, rather than species level, differences, when determining fungal–host interactions and defining commensal or pathogen behavior.

## Introduction

Fungi belonging to *Candida* genus are commonly harbored in up to 80% of human population, being the most prevalent commensal species inhabiting skin, gastrointestinal, and urogenital mucosae (1). Nevertheless, *Candida* spp. may give rise to opportunistic infections in susceptible hosts, such as in immunocompromised individuals (2), where they are responsible for the majority of mucosal and deep-seated candidiasis (3–5), or skin/mucosal barriers are affected, such as in extensively hospitalized and/or surgical patients where health care-associated candidemia often occur (6). This is an exponentially increasing threat, in particular, with the rise of azole resistant strains.

Genome-wide surveys of the microorganisms inhabiting the different human body microenvironments (i.e., gut, skin, oral mucosa, and urogenital tract) (7) have suggested the presence of strong and selective control on microbial communities that colonize and persist in peculiar environments. *C. albicans* efficaciously adapts to its host by escaping immune system recognition/detection (8, 9), as well as by acquiring specific drug-resistance (10, 11). The nature and extent of such changes have been studied *ex vivo* employing clinical or environmental isolates. Differences in cell wall composition have been accounted for escape from Dectin-1 (12, 13) or even TLR4 (14) recognition. Recently, Schönherr and collaborators explored the host immune response to different clinical *C. albicans* isolates (9), showing how fungal diversity determines diverse immune outcomes, where a delayed induction of Th17 response allows *C. albicans* colonization, while isolates inducing an immediate and strong Th17 response are rapidly cleared.

Different molecular typing approaches, including multilocus sequence typing (15, 16) and DNA fingerprinting (17) helped in unraveling the epidemiology and population structure of *C. albicans* (18). In particular, extensive plasticity of *C. albicans* genome has been documented, consisting of multiple major and minor clades, some of which showing a geographical imprinting. Although *C. albicans* isolates tend to show clonal origin, recombination and cross-chromosomal rearrangements may occur and appear more common under environmental stress conditions, such as prolonged drug treatments or exposure to host immune defenses; likely, these phenomena play a significant role in promoting microevolution and in turn virulence of this opportunistic pathogen.

Different fungal isolates of clinical origin vary in adhesion, dimorphic transition, and virulence (8, 10, 12), revealing an intriguing microbial adaptation possibly acting as an immune evasion strategy. *In vivo* and *in vitro* analysis of isolates from oral candidiasis has allowed identification of high, intermediate, and low virulent strains, with differences in metabolic pathways, such as the phosphate metabolism, known to have an important role in pathogenesis (8). One of the most potent virulence traits of *C. albicans in vivo* is its morphogenetic plasticity that allows fungal transition from yeast-to-hyphal forms, depending on environmental cues, such as morphogens (i.e., pH, CO_2_, temperature, presence of serum, and other micronutrients) (19). In particular, dimorphic transition to hyphal forms is crucial in promoting fungal escape from phagocytes (20–22), by reducing phagocyte-mediated ingestion and intracellular killing, a process crucial in infection control being pivotal to the accomplishment of both innate and adaptive immunity. Moreover, it is noteworthy that, from an evolutionary standpoint, both yeast and hyphal cells are required to sustain pathogenicity and host invasion, in fact neither yeast cell blocked strains nor hyper-filamentous mutants are fully virulent in experimental systemic infections (23–25).

In this study, we investigated the genomic determinants of the phenotypic changes observed in two *C. albicans* clinical isolates from Crohn’s disease (CD) patients. We probed their adaptation to the host by means of an *in vitro* infection model that employed different host cells, such as epithelial cells, peripheral blood mononucleated cells (PBMCs), and tissue-derived macrophages, namely microglial cells, known to play a major role as anti-*Candida* effector cells *in vitro*, as well as *in vivo* in preventing the outcome of experimental meningoencephalitis by *C. albicans* (26–28). Finally, we explored the pathogenic potential of such isolates *in vivo* by a murine model of peritoneal infection.

## Materials and Methods

### Fungal Strains and Culture Conditions

The clinical isolates of *C. albicans* used in this study, named YL1 and YQ2, were obtained from fecal samples of two pediatric CD patients in clinical remission, but having mucosal inflammation. *C. albicans* reference strain SC5314 (ATCC^®^ MYA-2876™) was also used in some experiments, as a control. Long-term storage of the isolates was carried out in 20% glycerol at −80°C. For phenotypic characterization and extraction of genomic DNA for whole-genome sequencing, YL1 and YQ2 yeast cells were seeded in liquid culture [Yeast Peptone Dextrose (YPD), with 1% yeast extract, 2% peptone, 2% glucose] at 28°C. For host–fungal interaction studies, fungal cells were cultured overnight at 37°C in Saboraud dextrose agar (SDA, Oxoid, Hampshire, UK); then, yeast cells were harvested, washed twice with sterile phosphate-buffered saline (PBS) (EuroClone, Wetherby, UK), counted and suspended in RPMI 1640 medium at the desired concentration.

### Whole Genome Analysis

Whole genome sequencing was carried out using the Illumina Genome Analyzer IIx (GAIIx) platform, and HiSeq2000 sequencing instrument. The standard Illumina protocol with minor modifications was followed for the creation of short-insert paired-end libraries (Illumina, Inc.). In brief, 2.0 µg of genomic DNA was sheared on a Covaris™ E220. The ~500 bp fragmented DNA was end-repaired, adenylated, and ligated to Illumina specific paired-end adaptors. To obtain a library of very precise insert size (500 bp) with the size deviation of ±25 bp, the DNA with adaptor-modified ends was size selected and purified using the E-gel agarose electrophoresis system (Invitrogen). After size selection, the library was PCR amplified using 10 PCR cycles. To create another library, at the same protocol above were added also a step at 72°C before adaptor ligation and a rapid cooling down to 4°C. This procedure allowed us to improve the coverage of increased GC-content regions of the genome. Each library was run in a fraction of a GAIIx flowcell lane in paired end mode of 2 × 151 bp read length, using Sequencing kit v4, or on HiSeq2000 in 2 × 101 bp read length, using TruSeq SBS Kit v3, both according to standard Illumina operation procedures (Illumina, Inc.). Primary data analysis was carried out with the standard Illumina pipeline. The purity of the signal from each cluster was examined over the first 25 cycles and chastity = Highest_Intensity/(Highest_Intensity + Next_Highest_Intensity) was calculated for each cycle. To remove the least reliable data from the analysis, reads were filtered according to chastity >0.6, for all but one of the first 25 bases. If there were two bases, the read was subsequently removed.

### Illumina Quality Control and SNP Calling

Sequence data are available at http://www.ebi.ac.uk/ena/data/view/ERP002505, under the accession number ERP002505. Illumina reads were subjected to quality control (filtering and trimming) using QC Toolkit (29). Paired reads were filtered with parameters: −l 70 (cutOffReadLen4HQ) and −s 20 (cutOffQualScore). As a result, reads with a PHRED quality score of less than 20 for more than 30% of their length were discarded. Moreover, reads were trimmed at the 3′ end for bases with a PHRED quality score of less than 30. Paired reads were mapped to the reference genomes [*C. albicans SC5314*, REfSeq NZ_AACQ00000000.1 (Stanford University), and WO-1, RefSeq AAFO00000000.1 (Broad Institute)], using Burrows Wheeler Aligner (BWA).^1^ The Genome Analysis Toolkit (GATK) was used for base quality score recalibration, Indel realignment, duplicate removal, and to perform SNP and Indel (insertion or deletion) discovery (30).

### Variant Imposition

The coding sequences of *C. albicans* YL1 and YQ2 isolates were created using variant imposition (a.k.a. consensus calling) in order to create uniform sets of genes for further analysis. Briefly, this technique inserted variants (SNPs and Indels) produced by GATK and specific for each strain into the coding sequences of the reference strains (SC5314 and WO-1) to create accurate coding DNA sequence (CDS) useful for phylogenetic comparison across strains.

### Gene Loss Prediction and Gene Ontology Enrichment of Lost, Duplicate, Truncated Genes

For each isolate, the alignments (BWA, then optimized by GATK) of reads vs the reference genome were analyzed and CDSs (taken from the genomic coordinates of SC5314 reference strain and reassembled in case of multiple exons) evidencing a zero coverage in at least one position were called as “lost.” In fact, these genes were apparently non-functional, broken, or lacked evident similarity with the reference. We considered that only a minority of genes (around 10%) had some positions (<5) with a coverage equal to 0. Although possibly affected by technical errors, especially in genes belonging to regions with low complexity or repetitive sequences that could be difficult to align, we considered this approach a valuable source of information and that false positive genes should appear in the majority of the strains analyzed and could be either false positives or acquisitions of the reference genome. Gene Ontology Term Finder tool on Candida Genome Database (CGD)^2^ were used for Functional Enrichment Analysis, using default parameters (therefore, considering as valid results with a *p*-value <0.05).

### Cell Wall Extraction and Sugars Quantification

The sugar composition of cell walls was analyzed as previously described (31). After sample preparation, monosaccharide analysis and quantification was performed using high-performance anion-exchange chromatography coupled with pulsed electrochemical detection (HPAEC-PAD) according to Dallies et al. (32). Glucose (for glucan content determination), mannose (mannan content), and glucosammine (for chitin) were identified by comparison with standard compounds and quantified according to calibration curves obtained for each sugar.

### Transcriptional Analysis

*Candida albicans* YL1 and YQ2 strains have been plated at a concentration of 1 × 10^7^ cell/ml in complete RPMI in presence or absence of 10% fetal bovine serum (FBS). RNA was isolated at 1 and 24 h from collected cells by the hot acid phenol extraction protocol. cDNA was synthesized from 200 ng of total RNA by reverse transcription using the one color Agilent kit (Quick Amp Labeling, Agilent Technologies, Santa Clara, CA, USA). Custom gene expression microarrays were provided by Agilent (Design ID 065138). Each condition tested was analyzed in triplicate. Microarrays were scanned with the Agilent scanner and primary analysis was carried out using the Feature extraction analysis software (Agilent Technologies). The LIMMA Bioconductor package on R was subsequently used for the data analysis. Locally weighed linear regression analysis was performed to normalize within arrays, and quantile normalization was carried out among arrays.

Comparisons have been performed between the different genetic backgrounds at the same time point. Differentially expressed genes (DEGs) were calculated by fitting a linear model to the data, followed by empirical Bayes statistics. Genes with *p*-value lower than 0.05 were considered to be significantly differentially expressed compared to the different condition. Data were expressed as fold change in logarithmic scale. Microarray data are public available to the GEO repository, under the accession number GSE85138. DAVID (33) and FungiFun 2.0 tools (34) were used for Functional Enrichment Analysis on DEGs, using default parameters (therefore considering as valid results with a *p*-value <0.05). Heatmap visualization of differential expressed genes, supported by hierarchical clustering (obtained using distance metric selection by Pearson correlation and average linkage clustering methods) was performed by Multiple Experimental Viewer tool (35).

### Phenotypical Characterization

#### Hyphal Formation

Yeast cells (10^6^ cells/ml) were grown in liquid YPD and YNB (0.67% Yeast Nitrogen Base w/o amino acids and (NH_4_)_2_SO_4_ and 2% glucose) media, both at 28°C and 37°C. After 3 and 7 days of incubation, hyphal formation was microscopically evaluated.

#### Resistance to Oxidative Stress

Yeast cell resistance to oxidative stress was evaluated by measuring the inhibition halo, as detailed elsewhere (36); briefly, *C. albicans* YL1 and YQ2 yeast cells (10^7^ cell/ml) were exposed to *tert*-Butyl-hydroperoxide (*t*But-OOH) 1 M. After 5 days of incubation at 28°C, the inhibition halo diameters (Ø) were measured. The percentage of sensitivity to oxidative stress was evaluated comparing the Ø of the clinical isolates with those of the reference SC5314 *C. albicans* strain.

#### Invasive Growth Assay

The ability of YL1 and YQ2 isolates to penetrate solid standard medium (YPD) was tested, as detailed elsewhere (37). Briefly, yeast cells (10^4^ cells/ml) were spotted on solid YPD plates and incubated at 28°C and 37°C. After 5 days of growth, cultures were stained with Coomassie blue and invasive growth was visually evaluated as signs of penetration onto the blue colored agar. The SC5314 *C. albicans* strain was used as positive control.

#### Growth on Different Carbon Sources

*Candida albicans* YL1 and YQ2 yeast cells (10^6^ cells/ml) were grown in solid YP added with the following carbon sources (2%): glucose, galactose, glycerol, ethanol, sucrose, oleate; the survival was evaluated after 3 days by colony-forming units (CFU) assay.

#### Antifungal Drug Resistance

Antifungal drug resistance was performed evaluating the growth of yeast cells (10^6^ cells/ml) in solid YPD added with different concentration of fluconazole and ketoconazole (0.01, 0.1, 0.5, 1, and 5 mM for each drug). SC5314 was used as control. Colony formation was evaluated after 24, 48, and 72 h of growth at 28°C.

### Human Epithelial Cells

The human epithelial colorectal adenocarcinoma cell line Caco-2 (ATCC^®^ HTB-37™) was grown in 75-cm^2^ tissue-culture flasks (Nalgen Nunc International, Naperville, IL, USA), using Dulbecco’s Modified Eagle’s Medium (EuroClone, Milan, Italy) supplemented with 10% heat-inactivated FBS, 2 mM l-glutamine, and 50 mg/ml gentamycin. Just before confluence, Caco-2 cell cultures were split 1:3 by standard methods. The cell cultures were maintained at 37°C under 5% CO_2_. Cells were seeded at a concentration of 6.5 × 10^5^/ml for the adhesion experiments and at a concentration of 8 × 10^5^/ml for the secretion assay.

### Human Peripheral Blood Mononuclear Cells Isolation, Exposure to *Candida*, and Cytokine Detection

The experimental plan was approved by the local Ethical Committee of Azienda Universitaria Ospedaliera Careggi (AUOC, Careggi Hospital, Florence; Italy), and written informed consent was obtained from all donors (approval document n. 87/10). Peripheral Blood Mononuclear Cells (PBMCs) were isolated from buffy coat samples by Ficoll-Hypaque density gradient centrifugation (Biochrom AG). Cells (10^6^ cells/ml) were cultured in RPMI 1640 medium supplemented with 10% heat-inactivated FBS, 2 mM l-glutamine, at 37°C under 5% CO_2_. YL1 or YQ2 cells were added to PBMCs cells at a E:T ratio of 1:10.

Supernatants were collected 5 days later for cytokine detection and conserved at −20°C until assayed. Cytokine detection was performed using the Milliplex^®^ MAP human cytokine/chemokine kit (Millipore), according to the manufacturer’s instructions.

### Phagocytosis Assay

*In vitro* phagocytosis assay has been performed as previously performed (38) with slight modification, according to the microorganism used. Briefly, BV2 cells (2 × 10^6^/ml, 100 µl/well) were seeded on poly-l-lysinated Lab-Tek II chamber slides (Nalgen Nunc International), incubated for 15 min at 37°C under 5% CO_2_ and then exposed to *C. albicans* YL1 or YQ2 yeast cells (4 × 10^6^/ml, 100 µl/well), suspended in complete RPMI [effector to target (E:T) ratio of 1:2]. The BV2 cells were further incubated for 1.5 or 3 h at 37°C under 5% CO_2_, as previously performed. Fifteen minutes before the end of incubation, Uvitex 2B (40 µl/well, Polisciences, Inc., PA, USA) was added, as a fluorescent dye that binds to fungal cell chitin (39, 40). BV2 cells were then washed three times with PBS to remove extracellular yeasts and fixed for 30 min with 4% paraformaldehyde (PFA) (Sigma-Aldrich) in PBS. ProLong Gold Antifade Reagent (PLGAR, Molecular Probes, Invitrogen, St. Louis, MO, USA) has been used to suppress the photobleaching effect and preserve the signals of labeled target molecules. The blue fluorescent *Candida* bound to BV2 cells as well as the unlabeled internalized fungi were visualized by epifluorescence microscopy (Nikon Instruments). At least 200 microglial cells from each sample were examined and the percentage of phagocytic cells (cells with intracellular yeasts) was defined as the ratio between the number of BV2 cells containing one or more unlabeled *Candida* to the total number of cells considered.

### Microglial Cells

The previously established murine microglial cell line BV2 was maintained by biweekly passages, as previous described (41) in RPMI 1640 medium supplemented with 10% heat-inactivated FBS gentamicin (50 mg/ml; Bio Whittaker, Verviers, Belgium) and l-glutamine (2 mM; EuroClone, Milan, Italy), hereafter referred to as just “complete RPMI medium.” The day before each experiment, cells were detached by vigorous shaking and seeded in fresh complete RPMI medium, at a concentration of 5 × 10^5^/ml on the day before each experiment.

### Epifluorescence Microscopy

Epifluorescence and differential interference contrast (DIC) microscopy were performed, as previously described (38), by using a Nikon Eclipse 90i imaging system, equipped with Nomarski DIC optics (Nikon Instruments Inc., USA). At each time point, samples were photographed with a DS-2Mv Nikon digital camera, and the resulting microphotographs were analyzed by using the Nikon NIS ELEMENTS version D3.1 software.

### Phagolysosome Acidification Assay

Visualization of the acidic *Candida*-containing phagosomes was performed as described previously (42), using the acidotropic dye LysoTracker Red DND-99 (Molecular Probes, Invitrogen) at a final concentration of 5 mM. The internalized *Candida* cells (no fluorescence) were visualized by epifluorescence microscopy (as opposed to bound/un-ingested yeast cells that showed a blue fluorescence because of the Uvitek 2B labeling). Acidification of the *Candida* containing phagosomes was indicated by the simultaneous appearance of LysoTracker Red DND-99 (red fluorescence) and *Candida* cells within the phagosomes that resulted as red fluorescence when merging the images. For quantitative analysis, the percentage of acidic phagosomes per image was calculated as the number of red phagosomes within the phagocytic cells divided by the total number of internalized *Candida* cells (unlabeled yeasts).

### Intracellular Survival Assay

The assay has been performed as previously described (43), with some modifications. Briefly, BV2 cells (2 × 10^6^/ml, 5 × 10^6^/2.5 ml) were incubated in 25-cm^2^ tissue-culture flasks (Nalgen Nunc International, Naperville, IL, USA) for 1.5 h with YL1 or YQ2 yeast cells (4 × 10^6^/ml, 10^7^/2.5 ml) in complete RPMI medium, at an E:T ratio of 1:2. Cells were harvested, washed twice with PBS, suspended in complete RPMI (time 0), and then seeded in 24-well polystyrene plates (Nalgen Nunc International, Naperville, IL, USA). At 0, 3, 6, and 20 h postinfection, the BV2 cells were lysed with 0.2% (v/v) Triton X-100 for 15 s to release the intracellular *Candida*. Serial dilutions were then plated onto SDA medium and CFUs were counted after 24 h incubation. The results were expressed as survival index (SI) at different time points. The SI was calculated as the ratio between CFU counts detected at 3, 6, and 20 h and the CFU counts obtained at time 0. Control experiments were carried out to verify that the treatment with Triton X-100 was not toxic for the *Candida* (data not shown).

### Adhesion Assay

Caco-2 cells (1.3 × 10^5^/200 μl, 6.5 × 10^5^/ml, 200 µl/well) were seeded in Lab-Tek II chamber slides, grown at 37°C to confluence (24 h), and then infected with a suspension of *C. albicans* YL1 or YQ2 yeast cells (5.2 × 10^5^/200 μl, 2.6 × 10^6^/ml, 200 µl/well) in complete RPMI (E:T ratio of 1:2). Caco-2 cells were incubated for 1.5 or 3 h at 37°C under 5% CO_2_. Fifteen min before the end of the incubation, samples were treated with Uvitex 2B (1%, 40 µl/well), washed three times with PBS to remove unbound yeast cells, and fixed for 30 min with PBS-buffered 4% PFA. After washing with PBS, Caco-2 cells were treated with PLGAR. The adherent *C. albicans* cells (blue fluorescence) were visualized by epifluorescence microscopy. At least 200 epithelial cells from each sample were examined and the percentage of adhesion was defined as the ratio between the number of Caco-2 cells with one or more *C. albicans* and the total number of Caco-2 cells examined.

### Secretory Assay

Caco-2 cells were seeded in 24 well-plates and incubated for 24 h to reach confluency. Then, *C. albicans* YL1 and YQ2 yeast cells (E:T ratio of 1:100) were added and plates were further incubated for additional 6 and 24 h. Cell-free supernatants were then collected and the levels of β-2 defensin was assessed by means of an enzyme immunoassay [β-2-Defensin (Human), Phoenix Pharmaceuticals Inc., Karlsruhe, Germany].

### Biofilm Formation

*Candida albicans* YL1 and YQ2 yeast cells were grown overnight in SDA plates at 37°C. Cells were harvested, washed with PBS, and suspended at 1 × 10^5^ or 1 × 10^6^ yeast cells/ml in complete RPMI. Then, 100 µl aliquots of each yeast cell suspension were placed in 96-well polystyrene microplates and incubated at 37°C for 24 and 48 h. After incubation, biofilm formation was quantified by the crystal violet assay, according to previously described protocols (22). To evaluate fungal dispersal from the biofilm, at 48 h, 100 µl/well were harvested and then the number of both yeasts and budding yeast cells were directly counted by a Burker chamber.

### Biofilm Thickness Measurement

*Candida albicans* YL1 and YQ2 yeast cells were seeded in six well-plates (Nalgen Nunc International), at 10^6^ cells/ml in complete RPMI and incubated for 24 and 48 h (37°C, 5% CO_2_). Then, wells were washed with PBS at room temperature, fixed with 4% PFA for 30 min at 4°C, and delicately washed again with cold PBS to remove PFA. The plates were then placed upside down and allowed to dry for 1 h. The samples were finally visualized using an optical microscope (Nikon Instruments Inc., USA). To measure biofilm thickness, the microscope sample tray was regularly moved (2 µm steps up) along the vertical axis, starting from the biofilm bottom (well surface) to the top of the fungal biofilm structure. Microphotographs were taken at each focal plan by means of a DS-2Mv Nikon digital camera, and the resulting photographs were analyzed [Nikon NIS ELEMENTS (version D3.1) software]. By manually counting the analyzed steps, the biofilm size was evaluated (micrometer); furthermore, by sequentially pulling together the acquired optical sections, each biofilm sample could be visualized in a movie format.

### *Candida* Cell Morphology

*Candida albicans* morphology has been assessed as previously reported (44) with some modifications. Briefly, YL1 and YQ2 yeast cells were seeded (1 × 10^6^/ml) in 24-well polystyrene plates with plastic cover slips (Thermanox) for 24 h (37°C, 5% CO_2_). Then, the cells were washed three times with PBS and each isolate was fixed with 2.5% glutaraldehyde (Electron Microscopy Sciences, Hatfield, PA, USA) in 0.1 M phosphate buffer (pH 7.2) for 18 h at 4°C. The samples were then carefully washed with 0.1 M phosphate buffer (pH 7.2). Post-fixation treatment was carried out for 1 h at room temperature with 1% osmium tetroxide (Electron Microscopy Sciences). Initial dehydration was accomplished by placing samples in ethanol as follows: 30% (once for 3 min), 50% (once for 6 min), 70% (once for 10 min), 90% (twice for 8 min), and 100% (three times for 8 min). The samples were critical point dried (Critical Point Dryer, CPD0-10, Balzers, Lichtenstein) before being gold coated (Sputter coater K550, Emitech Ltd., Ashford, UK) and examined with a scanning electron microscope (Nova-NanoSEM 450, FEI Company, OR, USA).

### Murine Model of Peritonitis

All experiments involving animals were performed in agreement with the EU Directive 2010/63, the European Convention for the Protection of Vertebrate Animals used for Experimental and other Purpose, and the National Law 116/92. The protocol was approved by the Modena and Reggio Emilia University Ethics Committee for animal care and use (Organismo preposto al benessere degli animali, OPBA). All the animals were housed in the animal facility of the University of Modena and Reggio Emilia (Authorization number 268/2011A). Female, 6–8 weeks old, FVB/N inbred mice (Charles River) were injected intraperitoneally with 0.2 ml of 3.5 × 10^7^ CFU/ml *C. albicans* YL1 or YQ2 (7 × 10^6^ CFU total), as previously described (45, 46). Eighteen hours postinfection, mice were sacrificed and peritoneal cavity was lavaged by injection of 4 ml of RPMI-1640, followed by gentle massaging of the peritoneal cavity. The peritoneal lavage fluids were then centrifuged at 3,000 rpm for 5 min and cellular fractions were suspended in 1 ml of PBS. Aliquots were then used for: (a) total cellular counts (by hemocytometer camera), (b) microscopic analysis and phagocytosis assessment (by May Grunwald Giemsa staining), (c) CFU evaluation after treatment with Triton X-100 (final concentration = 0.2%), (d) cytofluorimetric analysis after staining with phycoerythrin (PE) monoclonal antibody to mouse GR-1, and (e) evaluation of cytokine/chemokine content in the peritoneal lavages by Quantikine ELISA (R&D Systems) in duplicate. Kidneys and spleens were also removed, weighed, and mechanically homogenized, as previously described, prior to CFU analysis (45, 46).

### Statistical Analysis

**Figure 1:**
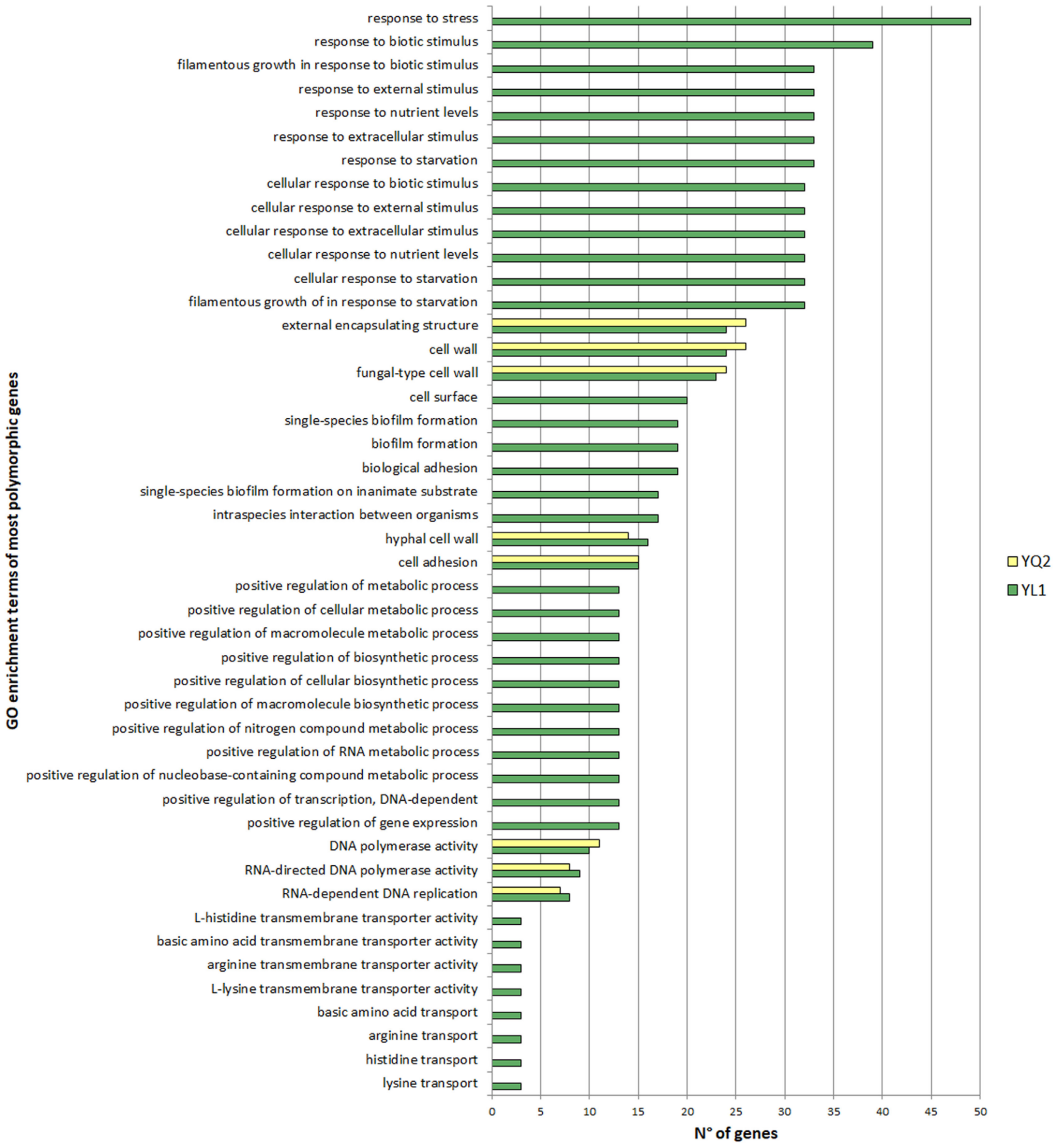
Gene ontologies enrichment analysis of the most polymorphic genes. Gene Ontology Term Finder tool on Candida Genome Database were used for functional enrichment analysis, using default parameters. Valid results were considered with a *p*-value <0.05.

To determine the statistical significance of the association of a particular Gene Ontology term with a list of genes, we referred to the indications in http://www.candidagenome.org. The Gene ontologies (GO) Term Finder tool on CGD calculates the *p*-value as the probability of having at least a number of genes in the list annotated to a particular GO term, given the proportion of genes in the whole genome that are annotated to that GO Term. Results with a *p*-value less than 0.05 were considered and included in the Figure 1 and Tables S1–S10 in Supplementary Material.

To define statistically significant differences in the sugar cell wall composition between strains, Kruskal–Wallis test with False Discovery Rate (FDR) correction was performed.

For *in vitro* and *in vivo* experiments, statistics was performed by one-way ANOVA with Bonferroni correction posttest or by the Student’s *t*-test, using GraphPad Prism (GraphPad Software Inc., La Jolla, CA, USA) with *p < 0.05; **p < 0.01; ***p < 0.001; ****p < 0.0001. In Tables 1 and 2, data are reported as mean ± SD (*N* = 3), in Table 3 as mean values ± SEM (*N* = 3/group).

**Table 1:** Biofilm thickness (expressed as µm) of *Candida albicans* isolates grown in RPMI in presence or absence of fetal bovine serum (FBS).

	24 h		48 h	
	RPMI + 10% FBS	RPMI	RPMI + 10% FBS	RPMI
*C. albicans YL1*	134	133.5*	182.6*	136.5
*C. albicans YQ2*	110	14	127	104.5

The asterisks indicate statistically significant differences (*p < 0.05).

**Table 2:** Biofilm structure and characteristics of *Candida albicans* isolates grown for 48 h in RPMI with fetal bovine serum.

	Thickness (μm)	Number of yeast cells released in the biofilm supernatants (×10^4^)	Number of budding yeast cells released in the biofilm supernatants (×10^4^)
*C. albicans* YL1	168*	19*	6.2*
*C. albicans* YQ2	96.6	5	1.3

The asterisks indicate statistically significant differences (*p < 0.05).

**Table 3:** Parameters assessed in the peritoneal cavity of YL1- and YQ2-challenged mice.

	Total peritoneal cells/ml (mean ± SEM)	PMN/ml (mean ± SEM)	IL-1β (pg/ml) (mean ± SEM)
*Candida albicans* YL1	5.66 × 10^6^ ± 0.27 × 10^6^	4.92 × 10^6^ ± 0.14 × 10^6^	33.2 ± 6.65*
*C. albicans* YQ2	3.36 × 10^6^ ± 1.18 × 10^6^	3.26 × 10^6^ ± 1.15 × 10^6^	11.1 ± 0.27

The asterisk indicates statistically significant difference comparing *C. albicans* YL1-infected mice vs *C. albicans* YQ2-infected mice (*p < 0.05).

## Results

### YL1 and YQ2 Clinical Isolates Showed Major Genetic Variations

A yeast collection of fecal isolates of pediatric CD patients was previously characterized phenotypically and genetically (Di Paola et al., submitted). In this study, the genome of the two of those clinical isolates, namely YL1 and YQ2, showing major genetic variations, was deeply investigated and compared to that of the reference *C. albicans* SC5314 strain. The whole genome-SNPs-based phylogenetic tree, including the principal fungal genera, identified these isolates as *C. albicans* species (Figure S1 in Supplementary Material). Analysis of genome-wide variation revealed a total of 178.336 SNPs/Indels and 168.981 SNPs/Indels for YL1 and YQ2 isolates, respectively, with nucleotide polymorphisms in CDS of 43–44%, as compared to the *C. albicans* SC5314 reference genome. Heterozygous variants ranged 55–65%, with respect to the SC5314 and WO-1 *C. albicans* references genomes (Table S1 in Supplementary Material). Specific homozygous regions for both YL1 and YQ2 were identified in chromosome 7 and in chromosome 2 and 4, respectively (Fig. S2).

Gene ontologies (GO) enrichment analysis showed that highly polymorphic genes in YL1 were those related to response to nutrients, external stimuli, starvation and genes, involved to filamentous growth, while highly mutated genes, common to both isolates, include those codifying for cell wall, adhesion, and hyphal cell wall categories (Figure 1).

For each sequenced genome, we identified genetic losses by comparison with the SC5314 reference genome [operationally, we defined an open reading frame (ORF) as “lost gene” with at least one region with zero coverage according to reads mapping location data; Table S2 in Supplementary Material and Section “Materials and Methods”]. We found 20 lost ORFs (7 of which were shared by YL1 and YQ2 and six were specific for YL1 and 7 for YQ2), which encoded for retro-transposable elements and factors involved in biofilm formation (among these, *HLA21* in both YL1 and YQ2, whereas the *ADH3* was present only in YQ2).

By comparing sequenced reads across the chromosomes, we found genomic regions with copy number variations. We identified duplication sites located especially in chromosome 2 and 6. Among the 11 duplicated ORFs present in both isolates, several were retro-elements; one of them, the *FGR24* gene, being a filamentous growth regulator. Other two members of *FGR* family (*FGR13* and *FGR14*) resulted duplicated in the YL1 genome, as well as a gene regulated by three iron-responsive transcriptional factors (Sef1p Sfu1p and Hap43p), known to be involved in *C. albicans* commensalism and pathogenesis (47). The YQ2 genome showed duplication of a member of telomere-associated (TLO) gene family (Table S3 in Supplementary Material), known for its variability in number and position in *C. albicans* (12). Furthermore, we searched for variants resulting in loss-of-function mutations, as SNPs producing internal stop and out-frame insertions or deletions. Across the YL1 and YQ2 genomes, we found 130 and 110 truncated ORFs, respectively (Tables S4 and S5 in Supplementary Material), while Indels resulted in out of-frame mutations that disrupted 22 and 16 genes, respectively in the two strains (Tables S6 and S7 in Supplementary Material). In the YL1 genome, we found prematurely stopped protein-coding genes involved in filamentous growth, cellular growth, and response to stimuli. We detected as disrupted, genes related to biofilm induction, such as *EFG1*, a key regulator of either the white/opaque switching or filamentous growth and virulence of *Candida*, whose loss of function has been related to increased commensal fitness (12), and *GPR1*, a G-protein-coupled receptor involved in filamentous growth and responsive to starvation and regulating *HWP1* (hyphal wall protein 1) and *ECE1* (endothelial converting enzyme 1) genes. It is noteworthy that among the 500 more polymorphic genes in our strains, *ECE1*, a gene recently indicated as the main determinant of *Candida* pathogenicity, is highly divergent from the reference sequence both in YL1 and YQ2. In particular, in *ECE1* gene, we found nucleotide polymorphisms generating 27 aminoacid (AA) changes in common to YL1 and YQ2 vs SC5314 and WO-1 *C albicans* reference strains, and 14 AA changes between YL1 andYQ2. The following genes associated with known functions (48), were found as truncated in YL1 and YQ2 isolates. In particular, the *PHR1* (pH responsive), *CYR1* (starvation and CO_2_ responsive), *SSK2* (responsive to osmotic stress), *RFG1* (repressor of filamentous growth), *FLO8* (transcription factor involved in hyphal formation and CO_2_ induced white-opaque switching), *ALS3* (cell wall adhesion induced and required for spider biofilm), *VRG4* (a GDP-mannose transporter), *SNT1* (a transposon mutation affecting filamentous growth), and *PDX1* (a spider biofilm-repressed gene).

### YL1 and YQ2 Clinical Isolates Exhibited Phenotypic Differences

A series of phenotypical peculiarities were assessed in YL1 and YQ2. First, the ability of the two isolates to form hyphae was evaluated under standard culture conditions (i.e., YPD and YNB without amino acids and supplemented with ammonium sulfate) at 28°C and 37°C. Unlike YL1, YQ2 showed hyphal formation after 3 and 7 days, at 37°C, both in YPD and YNB media (Figure S3A in Supplementary Material). Second, fungal growth was evaluated in the presence of different carbon sources. We found that both isolates showed a significant lower growth rate in media with non-fermentable carbon sources, namely, 2% of glycerol, ethanol, or oleate (Figure S3B in Supplementary Material). Third, we exposed YL1 and YQ2 to the oxidative stress induced by tert-butyl hydroperoxide treatment, as detailed in Section “Materials and Methods.” We found that the YL1 was more susceptible to the oxidative stress than YQ2 and SC5314 reference strain (Figure S3C in Supplementary Material). Fourth, we investigated the susceptibility to Ketoconazole and Fluconazole, two fungicidal agents known to interfere with the synthesis of fungal cell membrane. We found that both isolates showed resistance to Fluconazole, while only the YQ2 was resistant to Ketoconazole (Figure S4 in Supplementary Material).

Then, we tested the ability of the isolates to form biofilm in RPMI, in presence or absence of FBS and CO_2_, factors known to influence biofilm formation [(49, 50), p. 8 (51)]. Both YL1 and YQ2 isolates produced biofilm after 48 h of incubation, irrespectively of the presence of FBS; nevertheless, the thickness of YL1 biofilm was significantly higher than that produced by YQ2 (Table 1). Noteworthy, YQ2 did not produce biofilm at 24 h in the absence of FBS (Table 1); also CO_2_ markedly influenced biofilm formation by YQ2, which was indeed delayed in the absence of CO_2_ (data not shown). Furthermore, by investigating the morphological characteristics of the 48 h-old biofilms, we found marked differences in thickness mirrored by different cellular structures. As shown in Table 2, the number of both yeasts and budding yeast cells released by YL1 biofilm was significantly higher compared to that released by YQ2 biofilm.

**Figure 2:**
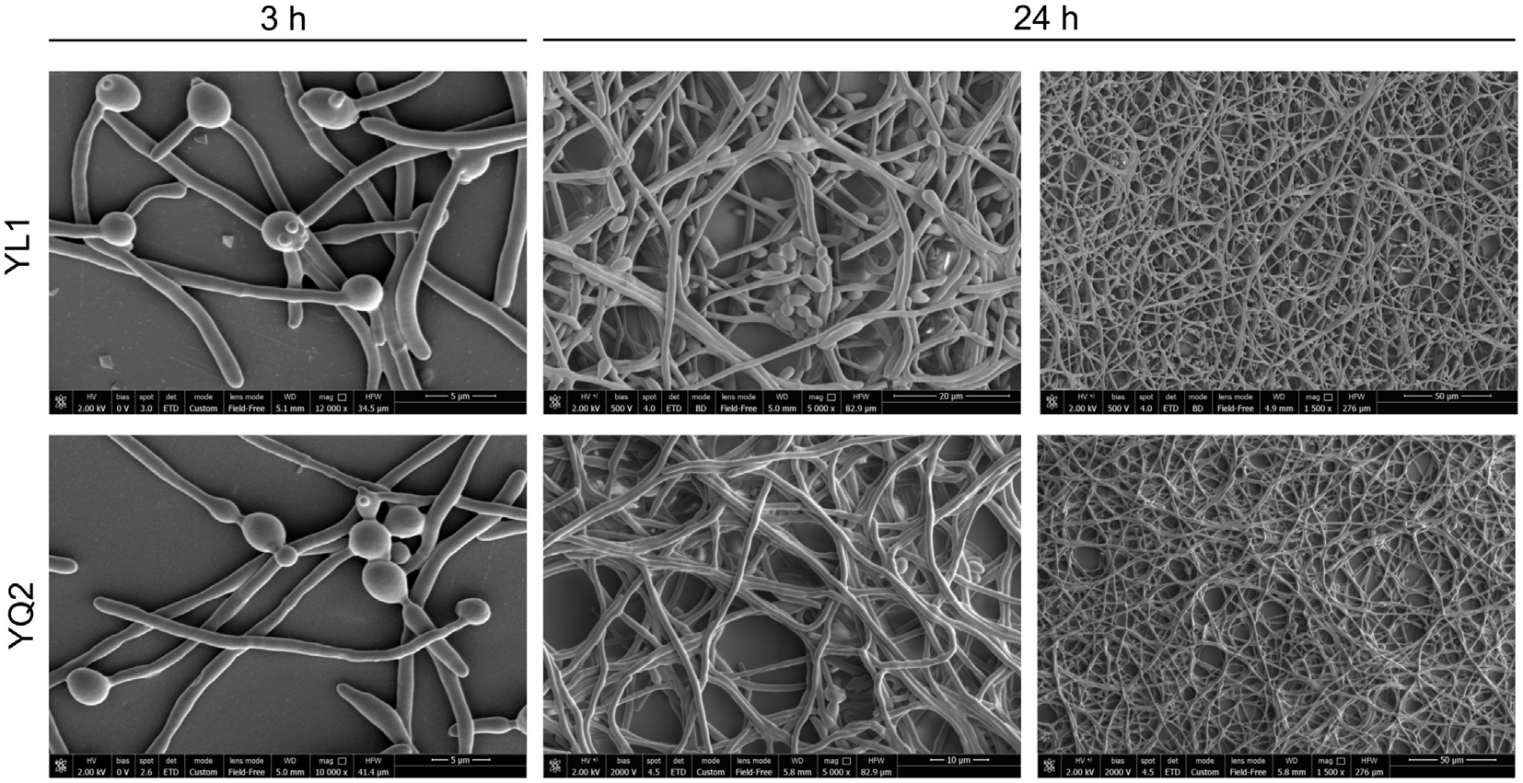
Kinetic analysis of biofilm morphology. To examine cell morphology, *Candida albicans* cells were seeded for 3 an 24 h at 37°C under 5% CO2. After fixation with 2.5% glutaraldehyde, cells were exposed for 1 h at room temperature with 1% osmium tetroxide. The samples were critical point dried before being gold coated and examined with a scanning electron microscope.

Finally, kinetic studies on the occurrence of dimorphic transition showed that after 3 h, both isolates formed long hyphae; interestingly, only the YL1 maintained also several budding yeast cells. At 24 h, the YL1 biofilm showed a particularly dense hyphal structure with interspersed germinating yeast cells that were not detectable in YQ2 biofilm (Figure 2).

### YL1 and YQ2 Differed in Cell Wall Sugar Components

**Figure 3:**
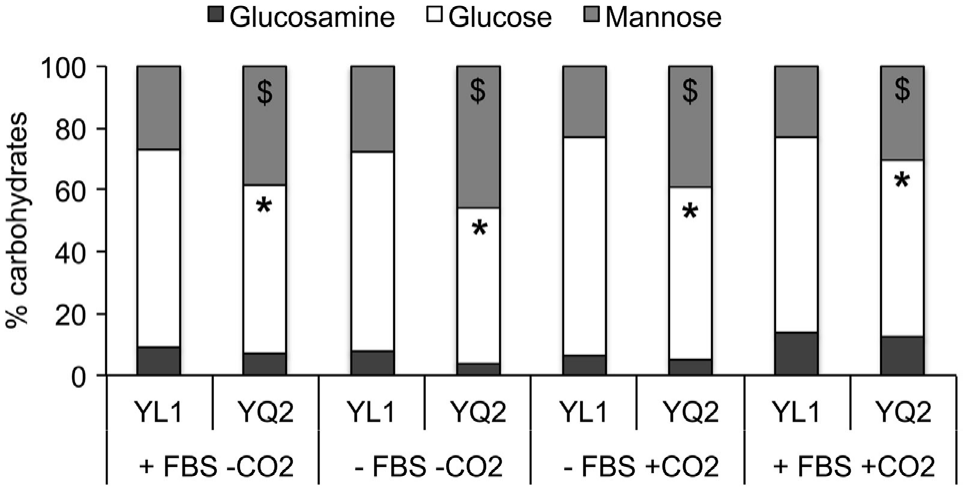
YL1 and YQ2 isolates showed different sugar cell wall composition. Sugars’ composition has been assessed on YL1 and YQ2 cell culture in RPMI in presence or absence of 10% fetal bovine serum and 5% CO_2_. Mean of three independent replicates is shown. *p < 0.05, YL1 glucose content vs YQ2 glucose content in the same cultural condition. **p < 0.01, YL1 mannose content vs YQ2 mannose content in the same culture conditions.

Sugars’ moieties and their exposure on fungal cell wall are determinant for *Candida* morphological switching and its ability to adhere to epithelial cells, escape immune system, and spread into host tissues (52). Therefore, we explored the YL1 and YQ2 isolates in terms of a possible diversity in cell wall composition. The cell wall of two isolates differed in terms of mannan and glucan content. In particular, YQ2 strain had higher mannan levels, while YL1 had more glucan in their cell wall. These differences were maintained in the different growth conditions, irrespectively of the fact that cultures had been performed with or without FBS and CO_2_ (Figure 3).

### Comparative Transcriptional Analysis on YL1 and YQ2 Isolates during Biofilm Formation

**Figure 4:**
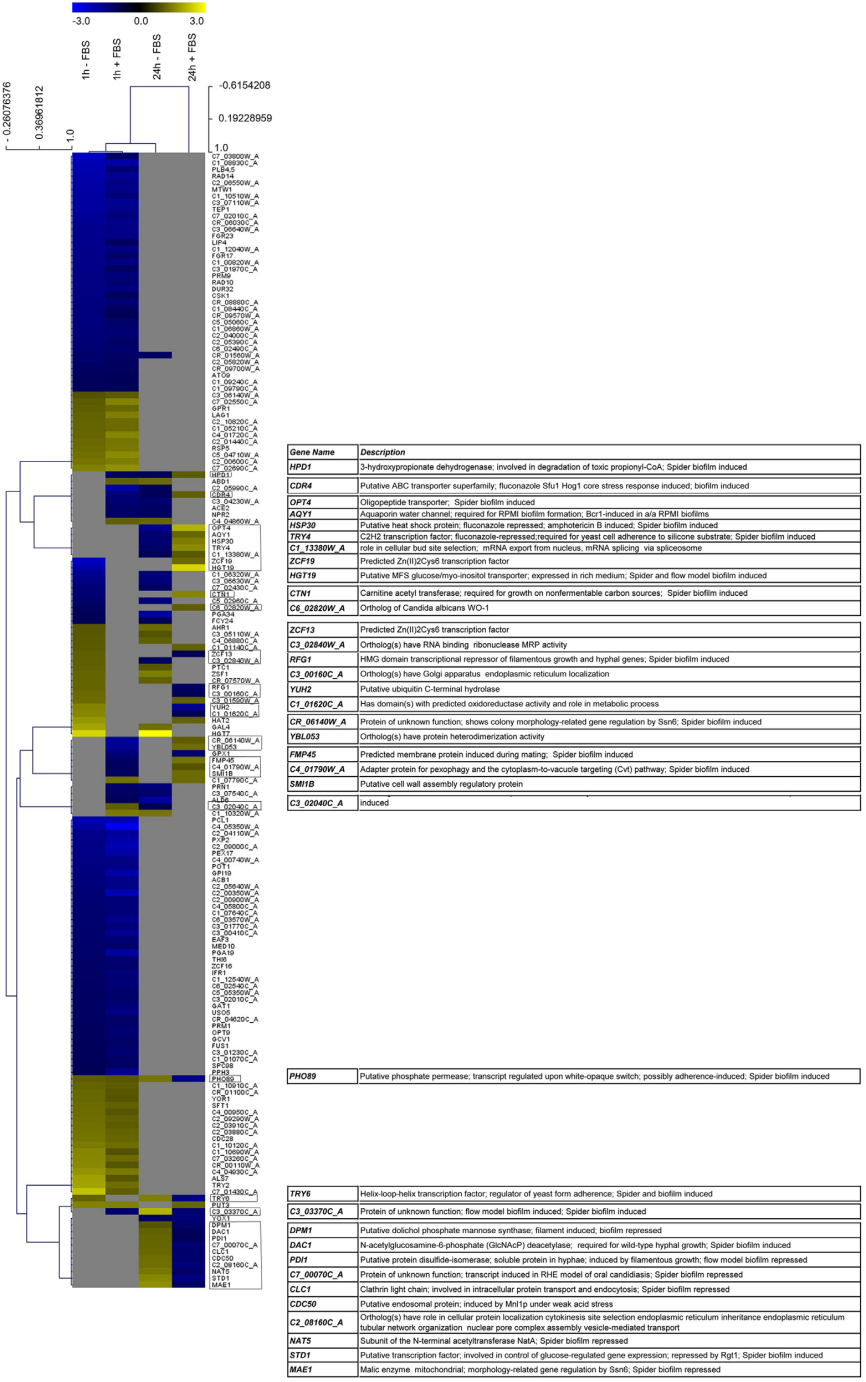
YL1 and YQ2 isolates showed different transcriptional regulation during biofilm formation. Heatmap visualization supported by hierarchical clustering (obtained using distance metric selection by Pearson correlation and average linkage clustering methods) of the gene differentially expressed in at least two condition. The complete list of differentially expressed genes is present as Table S8 in Supplementary Material.

With the aim to investigate the transcriptional reprogramming in the two isolates, we performed a transcriptional analysis on YL1 and YQ2 cultured at 37°C in RPMI both in presence and absence of 10% FBS (Figure 4). Operationally, we collected cells after 1 and 24 h of incubation. Since the analysis showed that several DEGs differentiated YL1 from YQ2 irrespectively of the presence of FBS, we herein describe only this comparison, but the complete analysis is reported in Tables S8–S10 and Figure S5 in Supplementary Material. We found 414 DEGs (approx. 10% of *Candida* genome) differencing YL1 and YQ2 transcriptional program after 1 h (151 up, 263 down; logFC < −1, logFC > 1, FDR < 0.05) (Tables S8 and Figure S5 in Supplementary Material). Interestingly, a lower number of genes were differentially expressed after 24 h; out of 273 DEGs, 110 were upregulated and 163 downregulated (Table S8 in Supplementary Material). It is noteworthy that seven genes, involved to some degree in biofilm formation, were downregulated after 1 h and upregulated after 24 h (Table S8 in Supplementary Material, YL1 vs YQ2).

Looking at specific DEGs, YL1 upregulated the *CDC28* gene (logFC = 1.23; Table S8 in Supplementary Material), which is known to be involved in cell morphology and biofilm formation (53, 54). Moreover, even if YL1 had a stop codon mutation in *GPR1*, its transcript resulted upregulated at 1 h (logFC = 1.24) (Table S8 in Supplementary Material), differential expression of the *ALS* genes (55, 56), known to be involved in cell–cell or host cell adhesion and invasive growth, was also observed. Compared to YQ2, YL1 upregulated *ALS7* gene (logFC = 1.03; Table S8 in Supplementary Material), while this gene was not differentially expressed later on, suggesting that adhesion and biofilm formation are the initial stages of adaptation.

Biofilm formation is known to be induced by stress genes (i.e., *CDR* genes) (55, 56); in our hands, YL1 showed downregulation of an important member of this family, *CDR4* after 1 h (log FC = −1.44) and its overexpression at 24 h (log FC = 1.14).

Increased production and secretion of proteins with agglutination properties may favor the cell cohesion within the biofilm. The transcriptional analysis showed that despite an initial upregulation of some components of the secretion machinery (*SEC3* and *SEC6*, Table S8 in Supplementary Material), which could account for the higher thickness of YL1 biofilm, YQ2 upregulated other genes involved in biofilm development, *SEC53, SEC21, SEC22, SEC61, SEC32, SEC17, SEC24*, at 24 h. Also lipid metabolism, membrane trafficking, and production of secretory vesicles from Golgi to endoplasmic reticulum (and *vice versa*), seemed to be differently affected in the two isolates (Tables S9 and S10 in Supplementary Material). Based on the DEG lists, we performed a functional enrichment over GO biological process categories and over FunCat categories and KEGG pathways, using DAVID tool (33) and the fungal specific FungiFun 2.0 tool (34). The comparison of the biological function enrichments allowed to identify processes differently affected during biofilm formation by YL1 and YQ2 isolates (Tables S9 and S10 in Supplementary Material). In particular, at 1 h, response to stress, organelle organization, as well as hyphal development, were functional enriched in the YL1 isolate with respect to YQ2. After 1 h, YL1 downregulated part of the amino acid biosynthesis genes, some involved in sulfur amino acid biosynthetic pathways (Table S10 in Supplementary Material, FunCat enrichment, *p* = 0.0035), known to affect the biofilm formation, as well as the metabolism of branched amino acids, like glutamate and aspartate (Table S10 in Supplementary Material, KEGG pathway enrichment, *p* = 0.03). Interestingly, YL1 showed a downregulation of cell wall biogenesis (*p* = 0.0060, Table S9 in Supplementary Material) and assembly (*p* = 0.00029, Table S9 in Supplementary Material), as soon as 1 h of incubation while after 24 h it showed a downregulation of cell wall mannoprotein biosynthesis pathways (*p* = 0.016, Table S10 in Supplementary Material), and an increased regulation of β-glucan biosynthetic processes (*p* = 0.0075, Table S10 in Supplementary Material), further corroborating the differences in cell wall composition observed between YL1 and YQ2. Interestingly, after 24 h, YL1 upregulated carnitine (Table S9 in Supplementary Material, *p* = 0.031) and acetyl-CoA (Table S10 in Supplementary Material, *p* = 0.049) metabolism, a process well known for promoting biofilm formation and fungal pathogenesis (57).

At 24 h, YQ2 upregulated genes involved in RNA processing and transcription regulation, as well as several genes involved in biosynthesis or salvage of nucleosides and translation (Table S9 in Supplementary Material). These cellular activities might be used by YQ2 in biofilm progression, for the production of extracellular matrix, which was not present in the bouquet-like structures of YL1 biofilm, which showed presence of budding and planktonic cells also at 24 h.

### YL1 and YQ2 Differently Interacted with Human Epithelial Cells

**Figure 5:**
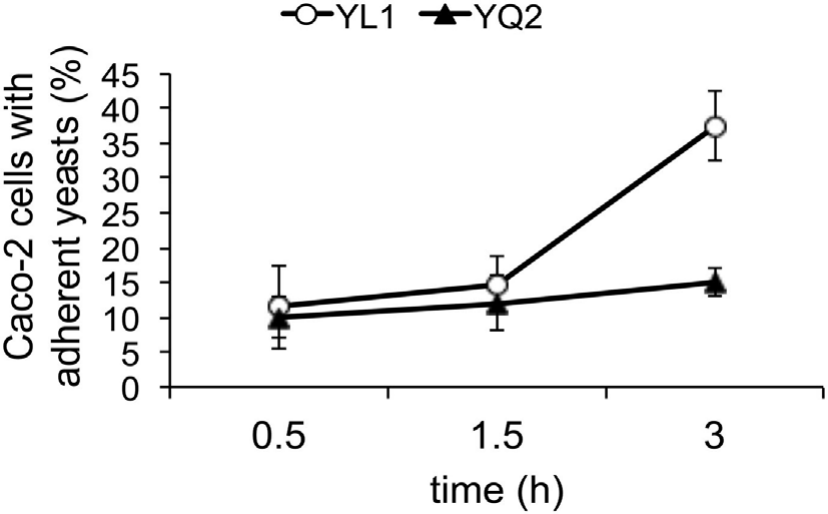
YL1 and YQ2 isolates differed in their ability to adhere to epithelial cells. Caco-2 cells were seeded in Lab-Tek II chamber slides, grown at 37°C to confluence (24 h) and then infected with a suspension of *Candida* in complete RPMI for 1.5 or 3 h at 37°C under 5% CO_2_. Uvitex 2B fluorescence of bound *C. albicans* was visualized by epifluorescence microscopy. At least 200 epithelial cells from each sample were examined and the percentage of Caco-2 cells with adherent yeasts was defined as the ratio of the number of Caco-2 cells with one or more *C. albicans* to the total number of Caco-2 cells examined. Data are represented as mean ± SD (*N* = 3). *p < 0.05.

We investigated the capacity of the two isolates to adhere to and to affect secretory response of human epithelial cells, by using the intestinal Caco-2 cell line. We found that the percentage adhesion was significantly higher with YL1 compared to YQ2 (Figure 5). Moreover, experiments were performed to determine whether Caco-2 cells challenged for 24 h with YL1 and YQ2 were affected in their human beta-defensin 2 (HBD-2) production; as shown in Figures 5, HBD-2 levels were significantly lower in Caco-2 cells exposed to YL1 that to YQ2 (47 vs 67 pg/ml, respectively). These results indicate that, although having a higher ability to bind to intestinal epithelial cells, YL1 induced lower HBD-2 response, compared to YQ2, thus further arguing on relevant differences between these two isolates in terms of pathogenic potential.

### YL1 and YQ2 Elicited Different Responses by Human PBMC

**Figure 6:**
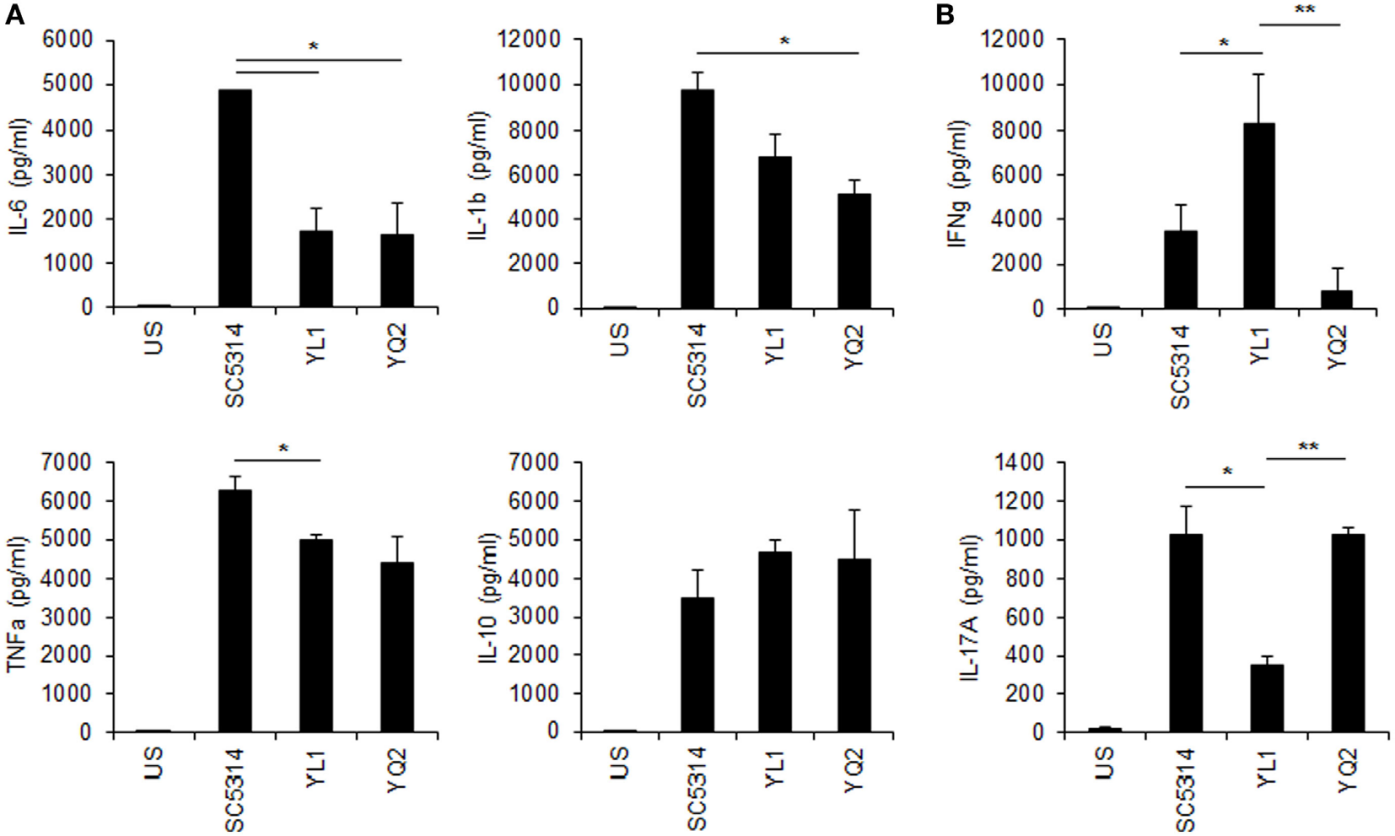
Peripheral blood mononucleated cells (PBMCs)’ cytokine response to YL1 and YQ2 infection. PBMC were exposed to *Candida* YL1 and YQ2 isolates (E:T = 1:10) for 5 days. Then, supernatants were harvested and assessed for innate **(A)** and adaptive **(B)** cytokine levels, as detailed above. Data are represented as mean ± SD. *p < 0.05, YL1 vs YQ2; **p < 0.01, YL1 vs YQ2. US indicates unstimulated cells.

Human PBMC were tested for cytokine response to YL1 and YQ2 *Candida* isolates, as described in Section “Materials and Methods.” Both clinical isolates induced a pro-inflammatory response (Figure 6A), but they differed in inducing cytokines related to the adaptive immune response. In particular, while YL1 induced a strong production of IFN-γ, YQ2-challenged PBMC responded to the fungus by releasing IL-17 (Figure 6B). These findings underlined the diversity between YL1 and YQ2 also in their interaction with host first line defenses, such as PBMC.

### YL1 and YQ2 Differential Susceptibility to Phagocyte-Mediated Host Defenses *In Vitro* and *In Vivo*

**Figure 7:**
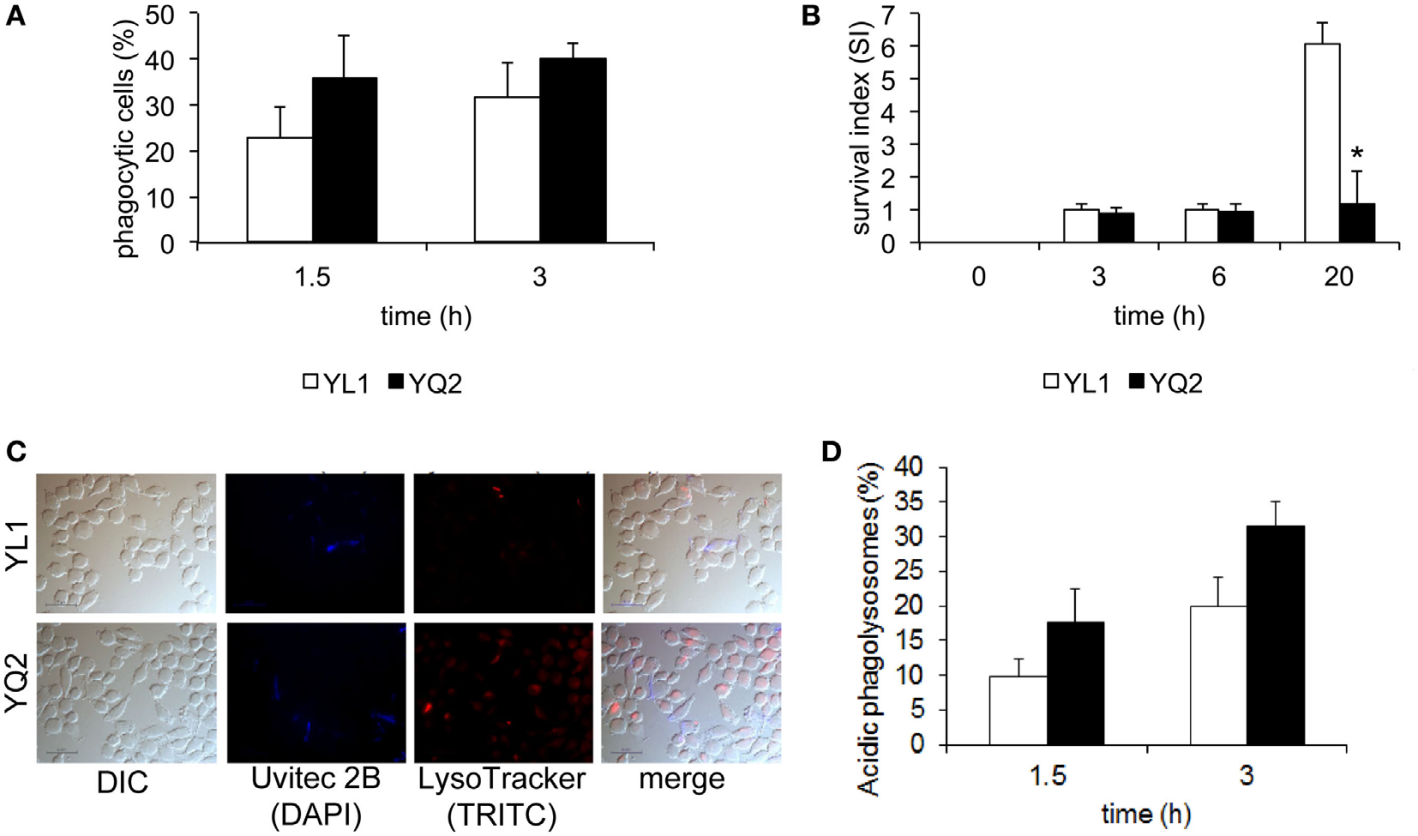
YL1 and YQ2 isolates differently resisted to microglial cell-mediated antifungal activity. **(A)** Susceptibility of the YL1 and YQ2 isolates to phagocytosis was assessed by epifluorescence microscopy using BV2 microglial cells. Oregon green 488 prelabelled yeast cells were exposed to BV2 cells (E:T = 1:5) for 1.5 and 3 h. At each end point, Uvitex 2B was added for 15 min; the cultures were then washed, fixed, and analyzed by epifluorescence microscopy. The percent of phagocytic cells was calculated as detailed in Section “Materials and Methods.” Data are shown as mean ± SD (*N* = 3). **(B)** Susceptibility of YL1 and YQ2 isolates to antifungal activity by microglia. Yeast cells were exposed to BV2 cells at E:T = 10:1. After 3, 6, and 20 h, the percent of antifungal activity was determined as detailed in Section “Materials and methods.” Data are shown as mean ± SD (*N* = 43). *p < 0.05, YL1 vs YQ2. **(C)** Acidification of phagolysosomes containing YL1 and YQ2 cells. Oregon green 488 prelabelled yeast cells were exposed to BV2 cells (E:T = 1:5); then, LysoTraker dye was added. After counterstaining with Uvitex 2B, samples were fixed and then visualized by epifluorescence microscopy. Representative images of acidification of phagolysosomes by means of epifluorescence are shown. **(D)** Percent of acidic phagolysosomes, containing YL1 or YQ2 cells. The values were calculated by evaluating the number of red-stained vacuoles among 200 yeast-containing vacuoles. Data are shown as mean ± SD (*N* = 3). *p < 0.05, YL1 vs YQ2.

We performed *in vitro* studies to characterize the interaction of YL1 and YQ2 clinical isolates with tissue derived phagocytes; particularly, the murine microglial cell line BV2 was chosen as an efficacious prototype of brain macrophages (26–28, 38, 41, 42). Using a previously established fluorescence-based assay (42), we measured YL1 and YQ2 susceptibility to phagocytosis, by evaluating number of BV2 cells that had ingested the yeast cells, at 1.5 and 3 h postinfection. As shown in Figure 7A, both isolates were efficiently engulfed by phagocytes, in a similar and time-dependent manner.

Next, we examined the ability of the two isolates to survive within phagocytes. To this aim, a modified protection assay was performed (42). BV2 cells were challenged with YL1 or YQ2 yeast cells for 1.5 h and then the non-ingested yeast cells were removed. Subsequently, the residual fungal load was determined immediately (time 0) or 3, 6, and 20 h later. We found that up to 6 h, the number of CFU recovered from BV2 cells remained stable by the time and was similar in the two isolates. In contrast, at 24 h, the intracellular CFU of YL1 greatly increased, reaching more than threefold higher levels than those observed with YQ2 (*p* < 0.05) (Figure 7B). These data indicated a relevant difference between YL1 and YQ2, in terms of susceptibility to phagocyte-mediated defenses.

To further investigate the events occurring after YL1 and YQ2 yeast cell ingestion, acidification of fungal cell-containing phagosomes was assessed, as detailed elsewhere (58). Briefly, YL1 and YQ2 yeast cells were incubated for 1 and 3 h with BV2 cells in the presence of LysoTracker, a marker of phagosome acidification (see Materials and Methods). Then, fluorescence microscopical examination was performed. Overlapping pictures obtained with different fluorescence channels were used to evaluate the successful acidification of *Candida*-containing phagosomes [simultaneous appearance of LysoTracker (red fluorescence) and unlabeled yeasts within the phagosomes] (Figure 7C). Then, we calculated the percentage of acidic phagosomes by counting the *Candida*-containing phagosomes within each BV2 cell. As shown in Figure 7D, after 1.5 h, the percentage of acidic YQ2-harboring phagosomes was higher than that of YL1-containing phagosomes. Indeed, about 18% of the YQ2 were associated with acidic phagolysosomes, while this percentage remained around 9% in the case of YL1 yeast cells; similar results were also observed after 3 h. In both cases, the differences between YQ2 and YL1 were statistically significant (*p* < 0.05). Taken together, this evidence implies that YL1 resists to phagocyte-mediated intracellular killing by inhibiting phagosome maturation much better than YQ2.

Finally, to investigate the *in vivo* virulence of the two isolates, a comparative study was performed by a mouse model of intraperitoneal infection. As detailed above, 7 × 10^6^ *Candida*/mouse were injected in the peritoneal cavity of mice, at time 0; 18 h later, the animals were sacrificed, and several parameters assessed. As depicted in Table 3, the number of host cells recovered in the peritoneal cavity of YL1-challenged mice was higher than that obtained from the YQ2-infected counterpart; also, the number of polymorphonuclear leukocyte (PMNs; GR-1 positive cells) followed the same trend. The levels of IL-1β detected in the peritoneal lavage of YL1-infected mice were significantly higher than those observed in YQ2-infected counterpart (Table 3), while little or no differences were found in terms of TNF-α and MCP1 (data not shown). Furthermore, significant differences were observed when assessing the ability of peritoneal exudate cells to ingest *Candida*, by microscopic analysis; a sixfold higher phagocytic activity was observed in YQ2-infected animals, with respect to YL1-infected animals. When evaluating the CFU in the organs, no statistically significant differences were observed between the two groups of mice; yet, YL1 CFU were slightly less detected in the peritoneal lavages and more in the spleens (data not shown).

## Discussion

Host defense against disseminated *Candida* infection primarily relies on the ability of professional phagocytes to ingest and rapidly destroy fungal cells, *via* phago-lysosome fusion, by cytocidal factors within an acidic environment that fulfill intracellular killing (a process known as “phagosome maturation”) (59). Nevertheless, *C. albicans* has evolved several strategies to escape such first line host-mediated defenses (60, 61). In particular, the morphogenetic status of the fungus finely affects macrophage-mediated effector and secretory response to the infection (62–64). Even more, fungal cells ensure their persistence and propagation within phagocytes, by affecting cytokine response and altering recruitment of late endocytic/lysosomal compartments (65, 66). Yet, the pathogenic mechanisms and relative attributes involved in *C. albicans* infection and colonization are still not completely understood. It is generally recognized that *C. albicans* virulence is a multifactorial peculiarity driven by genome plasticity (13–15, 40), namely its ability to efficiently adapt to different environments and deftly coordinate the expression of certain virulence genes (45–48, 67, 68). As an example, while disseminated candidiasis is mainly induced by the yeast form, mucosal diseases are attributed mostly to filamentous forms, like invasive hyphae and pseudohyphae (66).

In the present work, we show that two distinct *C. albicans* clinical isolates, obtained from CD patients, differ significantly from the reference genome, and also from each other. The major genetic variants differentiating the two strains are in genes related to biofilm production and susceptibility to first line host barriers, and correlate significantly to isolate-specific phenotypic differences. In line with the present findings, we have previously provided initial evidence that *C. albicans* isolates with distinct genotypic profiles (b and c karyotypes) exhibit different virulence profile (69); specifically, differences between b and c isolates have been observed in terms of ability to undergo morphological transition and to resist to macrophage-mediated intracellular killing (70). Here, the whole genome sequencing of two clinical isolates from CD patients (YL1 and YQ2) reveals a great number of highly polymorphic genes in comparison with the reference SC5314 *C. albicans* strain. Interestingly, polymorphic genes code for factors influencing fungal external structure, cell wall composition, fungal-type cell wall, hyphal cell wall determinants, and cell adhesion, suggesting increased variability in morphogenetic pathways. Furthermore, we identified variants resulting in gain- or loss-of-function mutations. Among such genes, we have found several regulators induced in response to environmental signals, like the response to pH, O_2_/CO_2_, as well as some repressors of yeast filamentation (48).

By assessing YL1 and YQ2 ability to undergo transition from yeast to hyphal form, we found that the former produces biofilm more rapidly than the latter. The structural and morphological analysis of their biofilms highlights substantial differences, in that, YQ2 biofilm is composed primarily of hyphal cells, while YL1 biofilm is characterized by a high-density hyphal structures with several interspersed germinating yeasts. It is well established that biofilm formation allows invading pathogens to create a safe sanctuary, in which sessile cells remain in a protected environment (2, 71, 72). Conversely, biofilm embedded-cells may be even limited by adverse conditions, such as a reduced nutrient availability; because of this, dispersion/detachment of cells would be beneficial to microbial survival. In addition, the release of cells from biofilm allows metastatic spreading and generation of novel communities at new locations [a mechanism reviewed in Ref. (73)]. This mechanism is particularly relevant in the case of *C. albicans*, since spreading cells, in yeast form, are responsible for candidemia and disseminated invasive candidiasis (74–77). In our hands, the number of both yeasts and budding yeast cells released by YL1 biofilm is significantly higher compared to that of YQ2 biofilm; this *in vitro* finding suggests that YL1 may have an increased virulence potential compared to YQ2 isolate.

Strikingly, the genomic and phenotypic differences between YL1 and YQ2 are corroborated by differences in gene expression. The causal relation between gene expression changes and the genetic differences cannot be determined based on our results, yet, the significant difference in gene expression are observed at early times (1 h), thus making unlikely that these changes are caused by differences in the structure of the hyphal mat or nutrient availability, which would be expected at later time points. Several examples support the driving role of genetic differences in explaining differential gene expression associated to yeast-to-hyphal form transition and biofilm formation. In one hand, at the pathway level, YL1 downregulates amino acid biosynthesis genes, in particular, sulfur amino acid biosynthetic pathways, known to induce the biofilm formation, as well as the metabolism of branched amino acids, like glutamate and aspartate. YL1 shows a downregulation of cell wall biogenesis as soon as after 1 h of incubation, while after 24 h a downregulation of cell wall mannoprotein biosynthesis pathways occurs, together with an increased regulation of β-glucan biosynthetic processes, further corroborating the differences in cell wall composition observed between YL1 and YQ2, especially in terms of mannan and glucan content. On the other hand, at the single gene level, YL1 shows a significantly increased expression of *GPR*1, despite having a stop codon mutation in the ORF of the gene; this finding suggests that the interrupted gene is expressed but is then translated as a sub-functional protein, thus interfering as a negative feedback loop in the control of its own expression (a condition likely not occurring in YQ2). Notoriously, *GPR1* encodes a plasma membrane G-protein-coupled receptor of the cAMP-PKA pathway, which triggers the signaling pathway that in turn regulates β-glucan masking, immune evasion, and hyphal growth through interaction with *GPA2* and regulation of *HWP1* and *ECE1* (78). Moreover, *ECE1* encodes for the hyphal borne toxin named candidalysin, known to directly damage epithelial membranes, *via* a danger response signaling pathway and epithelial cell activation. As a counterpart, *C. albicans* strains lacking this toxin do not activate or damage epithelial cells, and are avirulent in animal models of mucosal infection (79). In our hands, YL1 and YQ2 isolates show a significant divergence in candidalysin gene, further suggesting that different strategies of adaptation to the host have indeed occurred in the two isolates (likely also *via* occurrence of ECE1/candidalysis variants). Recent evidence highlighted that *C. albicans* isolates showing diverse pathogenicity in an oral infection model does not show differences in *ECE1* expression when exposed to keratinocytes (9), yet, this cannot exclude that *ECE1* driven differences in pathogenicity are associated to differences in protein function, thus suggesting the importance to obtain protein sequence in addition to gene expression information.

Of course, transcriptional differences observed at 24 h could be due to the specific features of the biofilms and different availability of nutrients and oxygen; as an example, YL1 upregulates carnitine and acetyl-CoA metabolism, a process well known for promoting biofilm formation and fungal pathogenesis (57).

These findings are in line with the diversity in yeast cell:hyphal cell ratio observed in their respective biofilm structures and correlate with the different adhesion ability of the two isolates.

The higher ability of YL1 to adhere to human intestinal cells *in vitro* further argues on the enhanced pathogenic potential of this clinical isolate, in agreement with previous findings showing that short germ tubes are more adherent than hyphae (79). Additionally, YL1 isolate induces lower levels of HBD-2; since this defensin exerts potent anti-*C. albicans* effects (80–82), we may interpret the minor YL1-induced secretory response as an additional mechanism to locally limit host innate immune reaction and in turn *Candida* pathogenicity.

The *in vivo* data provide initial evidence that indeed YL1 evokes a more pronounced inflammatory response than YQ2, as indicated by the higher peritoneal cell recruitment, PMN influx and local IL-1β response. Nevertheless, the efficacy of peritoneal cells to ingest *Candida* appears to be significantly lower in YL1 than in YQ2-infected mice, thus strongly suggesting that the former has the capacity to promptly evade first line host defenses. The CFU/organ measured at 18 h do not allow revealing significant differences between the two isolates in terms of dissemination through the host. Certainly, more in depth and dedicated studies are necessary to fully understand the *in vivo* pathogenic profile of the two isolates. In conclusion, our results suggest that *C. albicans* has a complex system of chronologically arranged processes by which morphological changes and adaptation to the host are driven; as expected, in agreement with previous findings (49, 83), serum is a potent stressor for *Candida*. Our observations indicate that the genetic features of YL1 allow it to faster adapt to environmental changes with respect to YQ2. On these bases, YL1 biofilm, particularly rich in yeast and budding cells, may be interpreted as a condition to better disseminate into the host. Further, the finding that the improvement of the YL1 phenotype requires inactivation of morphogenetic switch pathways suggests that the morphogenetic switch is a mechanism required for commensalism rather than pathogenesis. We, therefore, hypothesize that evolution shapes *Candida* genomic adaptation to the invasive phenotype by hampering important controllers of the morphogenetic switch; the functional effects of the concerted set of mutations discovered is in fact reflected by transcriptomic differences significantly affecting pathways involved in cell wall composition and filamentation. These mutations apparently negatively influence the ability of professional phagocytes to ingest and clear the engulfed yeast cells. The latter observation is supported by the fact that, albeit similarly internalized, the YL1 isolate is able to resist to intracellular killing better that YQ2, by inhibiting phagosome maturation within phagocytes *in vitro*; even more, YL1 can survive and replicate inside microglial cells, thus potentially taking further pathogenic advantage by the so called “Troy horse” phenomenon.

Overall, we demonstrate that phagocyte response to *C. albicans* is an isolate-dependent reaction; in particular, genomic background and adaptation to host environment deeply affect not only susceptibility to phagocytic defenses but also fungal cell wall composition as well as biofilm morphology. The present findings add further insights on the knowledge that virulence phenotype of *C. albicans* has to be multifactorial, possibly due to numerous, putative virulence tracts that may synergistically and/or differentially contribute to the multistep pathogen–host interplay, ultimately affecting the outcome of the infection. Whether these *in vitro* data will have an in patients counterpart has yet to be established. We may envisage that the different ability of the two isolates, YL1 and YQ2, to switch between yeast-to-hyphal morphologies, interact with epithelial cells and resist to phagocytes might have played a role in the onset and progression of the inflammatory process commonly present in bowel disease. The preliminary data, observed in the peritoneal infection model in mice, strongly argue on the different virulence of the two isolates. As detailed above, these isolates derived from CD patients, in which a pathologic interplay between host immune system and commensal organisms occurs. Accordingly, several studies indicate that *C. albicans* infections originate from commensal strains and that virulence-related traits may emerge as consequence of an impaired host immunity. Notoriously, the pathogenesis of CD is based on a genetically determined loss of immune tolerance to luminal antigens, in turn initiating an uncontrolled inflammatory process (84). Among the wide range of microbial antigens supporting an abnormal immune response to CD, yeast antigens have been described (85), as well as anti-*Saccharomyces cerevisiae* antibodies are detected as markers of CD; we cannot exclude that, because of common antigens, also *Candida* may act as endogenous immunogen, participating to the aberrant immune response in CD (86). We thus conclude that the co-occurrence of stop codon and missense mutations in pathways driving dimorphic transition and host infection reflects a selective balance between traits favoring yeast form dissemination and mucosal penetration, which in turn may depend on gut barrier permeability and immune system of the host environmental niches where the strain has evolved. Thus, to better understand the impact of fungi on host’s immune system, it is of increasing importance to investigate both the phenotypic and genomic characteristics at single isolate level, in multiple isolates, as well as to unravel the fine mechanism driving host cell–pathogen interaction.

## Author Contributions

DC, MDP, LR, EB, EP, and SaPe participated in study planning and designed the experiments. MDP, LB, IG, MG, JB, and MK carried out the genomic and data analyses. LR and MDP carried out the transcriptional analysis. MDP, LR, AA, SiPa, BC, and CDF performed experiments and aggregated data concerning phenotypical characterization of the isolates. AA, BC, SiPa, EP, EB, SP, and LR, performed *in vitro* and *in vivo* experiments. NT performed chemical analysis. CDF and PL provided the strains. DC, MDP, LR, EB, SaPe, and CDF discussed the data and wrote the manuscript. All authors read and approved the final manuscript.

## Conflict of Interest Statement

The authors declare that the research was conducted in the absence of any commercial or financial relationships that could be construed as a potential conflict of interest.

## Footnotes

^1^http://bio-bwa.sourceforge.net/.

^2^http://www.candidagenome.org/.
