# Supplementary material for: Genomic and Phenotypic Variation in Morphogenetic Networks of Two Candida albicans Isolates Subtends Their Different Pathogenic Potential
Source: Front Immunol. 2018 Jan 19;8:1997. doi: 10.3389/fimmu.2017.01997 (PMC5780349; doi:10.3389/fimmu.2017.01997)
Supplement: Supplementary file 1 [file data_sheet_1.docx]

Supplementary Material

**Genomic and phenotypic variation in morphogenetic networks**

**of two *Candida albicans* isolates subtends their different pathogenic potential**

Duccio Cavalieri^*^, Monica Di Paola, Lisa Rizzetto, Noemi Tocci, Carlotta De Filippo, Paolo Lionetti, Andrea Ardizzoni, Bruna Colombari, Simona Paulone, Ivo G. Gut, Luisa Berná, Marta Gut, Julie Blanc, Misha Kapushesky, Eva Pericolini^5^, Elisabetta Blasi and Samuele Peppoloni^*^

*** Correspondence:**

For the Biological section: Duccio Cavalieri, duccio.cavalieri@unifi.it

For the Immunological section: Samuele Peppoloni, [samuele.peppoloni@unimore.it](mailto:samuele.peppoloni@unimore.it)

# Supplementary Figures

**
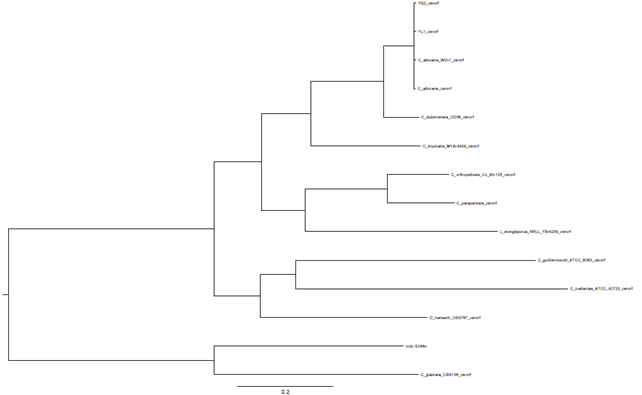
**

**Supplementary Figure 1. Phylogenetic relationships of *Candida spp.* with the principal genera of fungi.**

**
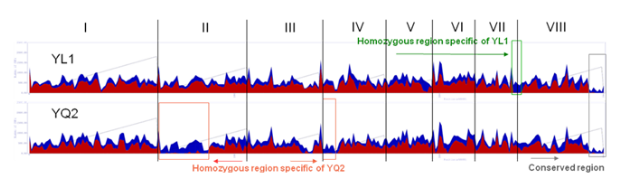
**

**Supplementary Figure 2. Analysis of heterozygosity at genome level.** Heterozygosity plot shows the number of SNPs in walking windows of 5000pb for each chromosome, including both heterozygous (in red) and homozygous (in blue) variants relative to the SC5314 reference, for each sequenced strains. In green and orange rectangles are indicated the specific homozygous region respectively for YL1 and for YQ2.

**
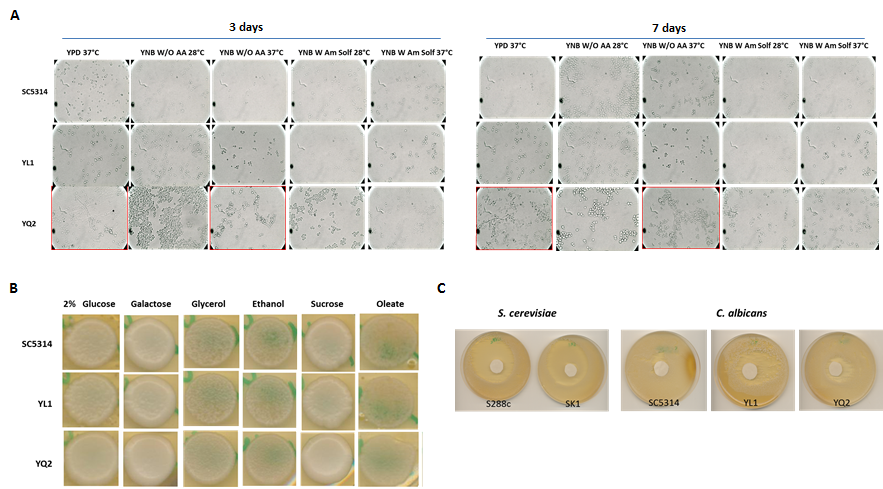
**

**Supplementary Figure 3. Phenotype analysis of *C. albicans* strains in standard laboratory condition. A.** Morphotype switching of YL1, YQ2 and SC5314 was observed in standard laboratory liquid culture condition, as YPD and YNB (without amino acids and with Ammonia sulfate) at 28°C and 37°C. **B.** Ability of growing in different carbon sources has been assessed by plating 104 cells on solid medium added by 2% of Glucose, Galactose, Glycerol, Ethanol or Oleate. Colony formation has been obsewrved after 3 days of growth at 28°C. **C.** Survival to oxidative stress evaluating the inhibition halo after 5 days of incubation with *tert*-Butyl-hydroperoxide (1 mM)

**
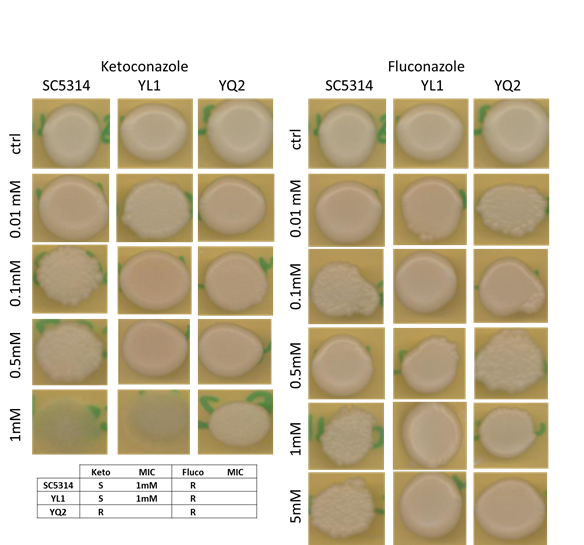
**

**Supplementary Figure 4. Resistance to ketoconazole and fluconazole antifungal treatment.** Antifungal drugs susceptibility was performed evaluating the growth of yeast cells (10^6^ cells/ml) in solid YPD added with different concentration of fluconazole and ketoconazole (0.01 mM, 0.1 mM, 0.5 mM, 1 mM and 5 mM for each drug). SC5314 was used as control. Colony formation was evaluated after 24 h, 48 h and 72 h of growth at 28°C.

**
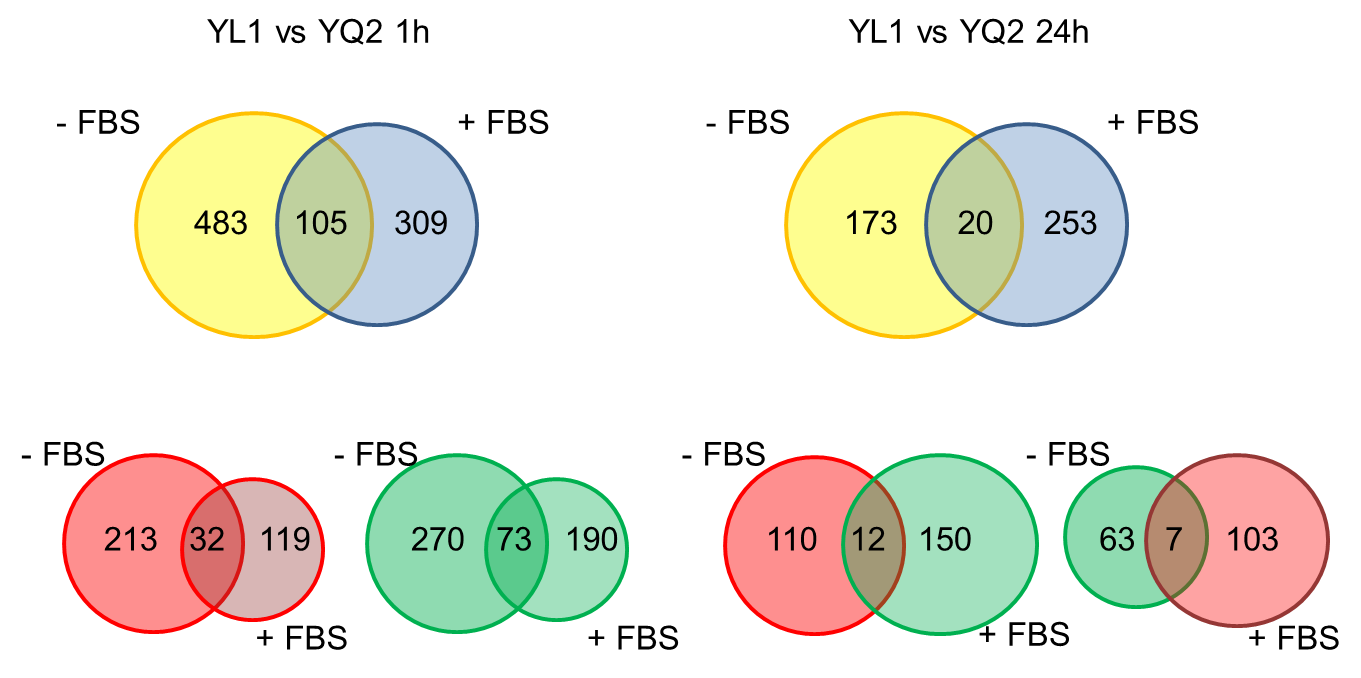
**

**Supplementary Figure 5. Representative scheme of the comparison done using transcriptional analysis.** Yellow circles indicate the total number of DEGs altered by the absence of FBS in YL1 compared to YQ2. Blue circles indicate the number of DEGs altered by the presence of FBS in YL1 compared to YQ2. Red circles indicate up-regulated genes in YL1 vs YQ2, while green circles downregulated genes at 1 hr and 24 hr in presence or absence of FBS.

# Supplementary Tables

**Supplementary Table 1. Alignment statistics of YL1 and YQ2 sequenced isolates against the reference genomes of *C. albicans* SC5314 and WO-1.** For each strain the table provides the amount of reads obtained after filtering and trimming process, the mapping and coverage information, the total number of SNPs/indels with respect to the references, and the fraction of homozygous and heterozygous polymorphisms.

| **Sequencing analysis** | | | | | | | | | | | | | |  |
| --- | --- | --- | --- | --- | --- | --- | --- | --- | --- | --- | --- | --- | --- | --- |
|  | | | | C. albicans SC5314 | | | | | C. albicans WO-1 | | | |  | |
| **ID strain** | | # filtered reads | # mapped | | # not mapped | % mapped | | # mapped | | # not mapped | % mapped | % coverage | |  |
| **YL1** | | 5151204 | 4959656 | | 191548 | 96,3 | | 4830749 | | 320455 | 93,8 | 34,6 | |  |
| **YQ2** | | 9870920 | 9507165 | | 363755 | 96,3 | | 9203880 | | 667040 | 93,2 | 66,2 | |  |
| **SNP/Indels vs the references** | | | | | | | | | | | | | |  |
|  | C. albicans SC5314 | | | | | | C. albicans WO-1 | | | | | | |  |
| **ID strain** | | # total        SNP/Indels | # total            homozygous | | # total         heterozygous | %          heterozygous | | # total              SNP/Indels | | # total            homozygous | # total         heterozygous | %        heterozygous | |  |
| **YL1** | | 178336 | 62260 | | 116376 | 65,1 | | 177193 | | 61542 | 115635 | 65,3 | |  |
| **YQ2** | | 168981 | 73904 | | 95450 | 56,4 | | 171152 | | 76188 | 94948 | 55,5 | |  |
| **Nucleotide polymorphisms (vs *C. albicans* SC5314 reference)** | | | | | | | | | | | | | |  |
|  | SNP/Indel | | | | | | heterozygous in CDS | | | | | | |  |
| **ID strain** | | # total | in non coding DNA sequence | | in CDS | % in CDS | | # total | | % | SNP | Indels | |  |
| **YL1** | | 178336 | 99806 | | 78530 | 44,0 | | 50784 | | 64,7 | 48278 | 2506 | |  |
| **YQ2** | | 168981 | 95615 | | 73366 | 43,4 | | 40833 | | 55,7 | 38796 | 2037 | |  |

**Supplementary Table 2. Summary of lost genes in the whole genome sequenced isolates.** **A.** Information about lost genes, including ORF number, systematic and standard name and description, as well as the percentage of ORF length with no coverage are reported. **B.** Gene Ontology of lost genes for each isolate by Gene Ontology Term Finder tool on Candida Genome Database (CGD). Significant at p<0.05.

|  | Systematic name | Standard name | Description | % ORF length with no coverage | |
| --- | --- | --- | --- | --- | --- |
|  |  |  |  | YL1 | YQ2 |
| orf19.6078.1 | C1_00280C_A | *RFC52* | Putative replication factor C complex protein; periodic mRNA expression, peak at cell-cycle G1/S phase; overlaps orf19.6079 | 100 | 100 |
| orf19.6078 | C1_00290W_A | POL93 | Predicted ORF in retrotransposon Tca8 with similarity to the Pol region of retrotransposons encoding reverse transcriptase, protease and integrase; downregulated in response to ciclopirox olamine; F-12/CO2 early biofilm induced | 100 | 100 |
| orf19.4070 | C2_09120C_A |  | Ortholog of Candida dubliniensis CD36 CD36_23270 | 100 | 100 |
| orf19.6079 | C1_00270W_A |  | Predicted ORF in retrotransposon Tca8 with similarity to the Gag region encoding nucleocapsid-like protein; repressed by ciclopirox olamine; filament induced; regulated by Rfg1, Tup1; overlaps orf19.6078.1 | 97.4 | 97.4 |
| orf19.7301 | CR_09050C_A |  | Has domain(s) with predicted DNA binding activity | 96 | 91.8 |
| orf19.4069 | C2_09110C_A |  | Protein of unknown function; repressed by alpha pheromone in SpiderM medium | 95.3 | 95.3 |
| orf19.99 | C6_00970C_A | *HAL21* | Putative phosphoadenosine-5'-phosphate or 3'-phosphoadenosine 5'-phosphosulfate phosphatase; possible role in sulfur recycling; ortholog of S. cerevisiae Met22; predicted Kex2 substrate; F-12/CO2 biofilm induced | 55 | 55 |
| orf19.2371 | C7_02610C_A |  | Putative Gag protein of retrotransposon Tca2; separated by a stop codon from Pol protein orf19.2372; likely translated as single polyprotein that includes Gag, reverse transcriptase, protease, and integrase; rat catheter biofilm repressed | 100 | - |
| orf19.7278 | C1_14630C_A |  | Similar to a region of the Tca2 (pCal) retrotransposon, which is present in strain hOG1042 as 50 to 100 copies of a linear dsDNA; rat catheter biofilm repressed | 100 | - |
| orf19.103 | C6_01010W_A | *KAR5* | Ortholog of S. cerevisiae Kar5; involved in nuclear membrane fusion during karyogamy; induced by alpha factor | 100 | - |
| orf19.2372 | C7_02600C_A |  | Pol protein of retrotransposon Tca2; separated by a stop codon from Gag protein orf19.2371; likely translated as single polyprotein with Gag, reverse transcriptase, protease, and integrase; rat catheter biofilm repressed | 86 | - |
| orf19.6465 | C7_02430C_A |  | Ortholog of *C. dubliniensis CD36* : Cd36_83910, *Candida tropicalis MYA-3404* : CTRG_05087 and *Candida albicans WO-1* : CAWG_00654 | 70.7 | - |
| orf19.6469 | C7_02420C_A |  | Predicted ORF in retrotransposon Tca11 with similarity to the Gag-Pol region of retrotransposons, which encodes nucleocapsid-like protein, reverse transcriptase, protease, and integrase | 36 | - |
| orf19.2669 | C4_03230C_A |  | ORF in retrotransposon Tca4; similar to Pol region of retrotransposons encoding reverse transcriptase, protease, integrase; downstream of RHD2, similar to the Gag region encoding nucleocapsid-like protein; rat catheter biofilm induced | - | 100 |
| orf19.2668 | C4_03250C_A | *RHD2* | Predicted ORF in retrotransposon Tca4 with similarity to the Gag region encoding nucleocapsid-like protein; overlaps blocked reading frame orf19.2668.1; yeast-enriched transcript; rat catheter biofilm induced | - | 100 |
| orf19.2219 | C2_08240W_A | *ORF298* | Predicted ORF in retrotransposon Tca3; similar to Gag-Pol; a-specific transcript, alpha-factor induced; rat catheter biofilm repressed; mRNA binds She3 | - | 100 |
| orf19.133 | C6_01250W_A |  | Protein of unknown function; induced by nitric oxide independent of Yhb1p | - | 100 |
| orf19.104 | C6_01020W_A |  | Protein of unknown function; induced by alpha pheromone in SpiderM medium | - | 92.7 |
| orf19.4919 | C1_12890W_A |  | Has domain(s) with predicted DNA binding, nucleic acid binding activity | - | 98.1 |
| orf19.4505 | C2_04470W_A | ADH3 | Putative NAD-dependent (R,R)-butanediol dehydrogenase; regulated by white-opaque switch; induced by nitric oxide independent of Yhb1; Spider biofilm induced | - | 82.9 |

**Supplementary Table 3. Summary of duplicated genes in the whole genome sequenced isolates.** **A.** Information about duplicated genes, including ORF number, systematic and standard name and description, as well as comparison between ORF position in the *C. albicans* SC5314 reference strain and position in each isolate, are reported. **B**. Gene Ontology of duplicated genes for each isolate by Gene Ontology Term Finder tool on Candida Genome Database (CGD). Significant at p < 0.05.

1. Information about duplicated genes

| ORF | Systematic name | Gene name | ORF position (chr) in SC5314 reference strain | duplication site (chr) in the isolate |  |  | Description |
| --- | --- | --- | --- | --- | --- | --- | --- |
| orf19.6115 | C1_00010W_A |  | 1 | 6 | YL1 | YQ2 | orf_classification=Dubious |
| orf19.6834.10 | CR_08820C_A | TAR1 | 8 | 6 | YL1 | YQ2 | Ortholog of *S. cerevisiae* Tar1p; Transcript Antisense to Ribosomal RNA; encoded within the 25S rRNA gene on the opposite strand; induced by Tbf1 |
| orf19.7271 | C1_14530W_A |  | 1 | 6 | YL1 | YQ2 | Predicted ORF in Assemblies 19 20 and 21 transcription detected in high-resolution tiling array experiments |
| orf19.7272 | C1_14550C_A |  | 1 | 6 | YL1 | YQ2 | Predicted ORF in Assemblies 19 20 and 21 transcription detected in high-resolution tiling array experiments |
| orf19.7274 | C1_14560C_A |  | 1 | 6 | YL1 | YQ2 | Predicted ORF in retrotransposon Zorro2 with similarity to retroviral reverse transcriptase proteins |
| orf19.7275 | C1_14570C_A | FGR24 | 1 | 6 | YL1 | YQ2 | Filamentous Growth Regulator; Protein encoded in retrotransposon Zorro2 with similarity to retroviral endonuclease-reverse transcriptase proteins; lacks an ortholog in S. cerevisiae; transposon mutation affects filamentous growth |
| orf19.7277 | C1_14580C_A |  | 1 | 6 | YL1 | YQ2 | Predicted ORF in retrotransposon Zorro2 with similarity to zinc finger-containing retroviral nucleocapsid proteins un-merged from orf19.7275 in a revision of Assembly 21 based on comparative genome analysis and new sequence data |
| orf19.7270 | C1_14520W_A |  | 1 | 5 | YL1 | YQ2 | Protein of unknown function; Spider biofilm repressed; Ortholog of C. parapsilosis CDC317 CPAR2_808740 Candida tenuis NRRL Y-1498 CANTEDRAFT_95761 Debaryomyces hansenii CBS767 DEHA2G11946g and Candida dubliniensis CD36 CD36_70220 |
| orf19.5469 | C3_00030C_A |  | 3 | 4 | YL1 | YQ2 | Protein with a predicted DEAD-like DNA/RNA helicase domain; shows colony morphology-related gene regulation by Ssn6; overlaps orf19.5472; Spider biofilm repressed; Predicted ORF in Assemblies 19 20 and 21 shows colony morphology-related gene regulation by Ssn6p overlaps orf19.5472 |
| orf19.5472 | C3_00040W_A |  | 3 | 4 | YL1 | YQ2 | ORF Predicted by Annotation Working Group overlaps orf19.5469 |
| orf19.5474 | C3_00010C_A |  | 3 | 3 | YL1 | YQ2 | Predicted ORF in Assemblies 19 20 and 21 transcriptionally activated by Mnl1p under weak acid stress transcription detected in high-resolution tiling array experiments; Spider biofilm repressed |
|  |  |  |  |  |  |  |  |
| orf19.5475 | C3_00020W_A |  | 3 | 2 | YL1 |  | Sef1p- Sfu1p- and Hap43p-regulated gene |
| orf19.559 | CR_05240C_A | FGR14 | 8 | 2 | YL1 |  | filamentous growth influenzing gene; Protein encoded in retrotransposon Zorro3 with similarity to retroviral endonuclease-reverse transcriptase proteins; lacks an ortholog in S. cerevisiae; transposon mutation affects filamentous growth |
| orf19.562 | CR_05250C_A | FGR13 | 8 | 2 | YL1 |  | filamentous growth influenzing gene; Protein encoded in retrotransposon Zorro3 with a potential zinc finger; lacks an ortholog in S. cerevisiae; transposon mutation affects filamentous growth |
| orf19.6114 | C1_00020C_A |  | 1 | 3 | YL1 |  | Protein of unknown function; transcript detected on high-resolution tiling arrays |
|  |  |  |  |  |  |  |  |
| orf19.6337 | C6_00030W_A | TLO13 | 6 | 2 |  | YQ2 | Member of a family of telomere-proximal genes of unknown function; may be spliced in vivo; overlaps orf19.6337.1, which is a region annotated as blocked reading frame |
| orf19.6338 | C6_00020W_A |  | 6 | 2 |  | YQ2 | Ortholog of *Candida albicans* WO-1 CAWG_05352 |
| orf19.7545 | CR_00010C_A |  | 8 | 2 |  | YQ2 | Protein similarity to mutator-like element (MULE) transposase |

1. Gene Ontology of duplicated genes for each isolate by Gene Ontology Term Finder tool on Candida Genome Database (CGD)

| *GO ENRICHMENT by Gene Ontology Term Finder tool (CGD)* | | | |  |  |  |
| --- | --- | --- | --- | --- | --- | --- |
|  | GO term | | Adj P-value | FDR | Genes annotated to the term |  |
| *IN COMMON* | TAR1 | molecular function unknown | |  |  |  |
|  | FGR24 |  |  |  |  |  |
| *ALL YL1* | FGR14 | filamentous growth of a population of unicellular organisms in response to starvation | 0.02549 | 0.00% | FGR13. FGR14 |  |
|  | FGR13 | cellular response to biotic stimulus | 0.03308 | 2.00% | FGR13. FGR14 |  |
|  |  | filamentous growth of a population of unicellular organisms in response to biotic stimulus | 0.03555 | 1.33% | FGR13. FGR14 |  |
|  |  | response to biotic stimulus | 0.04761 | 1.00% | FGR13. FGR14 |  |
|  |  | cellular response to starvation | 0.05293 | 0.80% | FGR13. FGR14 |  |
|  |  | response to starvation | 0.0571 | 0.67% | FGR13. FGR14 |  |
|  |  | cellular response to nutrient levels | 0.06553 | 0.57% | FGR13. FGR14 |  |
|  |  | cellular response to extracellular stimulus | 0.06706 | 0.50% | FGR13. FGR14 |  |
|  |  | cellular response to external stimulus | 0.06899 | 0.44% | FGR13. FGR14 |  |
|  |  | response to nutrient levels | 0.07095 | 0.40% | FGR13. FGR14 |  |
|  |  | response to extracellular stimulus | 0.07214 | 0.36% | FGR13. FGR14 |  |
|  |  | response to external stimulus | 0.08674 | 0.33% | FGR13. FGR14 |  |
| *ALL YQ2* | TLO13 | zinc ion binding | 0.04857 | 0.25% | TLO13 |  |

**Supplementary Table 4. Summary of stopped genes in the whole genome sequenced YL1 isolate.** **A**. Information about stopped genes, including ORF number, systematic and standard name and description, as well as ORF position in the *C. albicans* SC5314 reference strain are reported. **B**. Gene Ontology of stopped genes by Gene Ontology Term Finder tool on Candida Genome Database (CGD). Significant at p < 0.05.

1. Information about stopped genes

| ORF | parent ORF | Gene | ORF position (chr) in SC5314 reference strain | | | Description | | Note | | Alias | |  |
| --- | --- | --- | --- | --- | --- | --- | --- | --- | --- | --- | --- | --- |
| orf19.2483 | orf19.2483 | RIM1 | chr1 | 1189582 | 1190013 | | Putative single-stranded DNA-binding protein B protein level decreases in stationary phase cultures | | orf_classification=Uncharacterized | | orf19.10019,IPF24873.1,IPF12405.2,Contig4-2966_0015,orf6.6164,CA3804,CaO19.10019,CaO19.2483,CAWG_00837 | |
| orf19.1232 | orf19.1232 | VRG4 | chr1 | 1674241 | 1675356 | | GDP-mannose transporter B essential B required for glycosylation C hyphal growth B functional homolog of S. cerevisiae Vrg4p C which imports GDP-mannose from cytoplasm to Golgi for protein and lipid mannosylation B no mammalian homolog | |  | | GOG5,orf19.8817,IPF26079.1,IPF13284.2,Contig4-2208_0006,orf6.5085,CA2980,CaO19.1232,CaO19.8817,CAWG_00647 | |
| orf19.4753 | orf19.4753 | PFK26 | chr1 | 1948259 | 1951699 | | Putative 6-phosphofructo-2-kinase B protein repressed during the mating process B planktonic growth-induced gene | | orf_classification=Uncharacterized | | orf19.12217,IPF17562.3,IPF9943.2,Contig4-1843_0004,orf6.1050,orf6.4023,CA2215,CaO19.4753,CAWG_00532 | |
| orf19.3288 | orf19.3288 | NMA111 | chr1 | 199322 | 202255 | | Putative serine protease and general molecular chaperone B macrophage-induced gene B downregulated in core stress response B fungal-specific 8no human or murine homolog 9 B merged with orf19.3288.1 in Assembly 21 | | orf_classification=Uncharacterized | | orf19.10798,IPF23911.1,IPF12606.1,orf6.1778,CA0693,orf19.6008.1,orf19.3288.1,IPF12606.5eoc | |
| orf19.992 | orf19.992 | LKH1 | chr1 | 2321637 | 2323508 | | Putative leukotriene A 84 9 hydrolase B repressed during the mating process B Hog1p-induced | | orf_classification=Uncharacterized | | orf19.8607,IPF26288.1,IPF6778.2,Contig4-2932_0021,orf6.5806,LKH1.3,CA3524,CaO19.8607,CaO19.992,LKH1.3f,CAWG_00384 | |
| orf19.5241 | orf19.5241 | SNT1 | chr1 | 2669672 | 2672677 | | Protein similar to S. cerevisiae Snt1p C which is an NAD-independent histone deacetylase B not essential for viability B transposon mutation affects filamentous growth | |  | | orf19.12706,IPF12584,IPF2015.3,IPF12584.2,Contig4-3067_0022,orf6.768,orf6.7701,CA5006,CaO19.5241,CAWG_00219 | |
| orf19.4956 | orf19.4956 | RPN1 | chr1 | 2898458 | 2901442 | | Putative 19S regulatory particle of the 26S proteasome B regulated by Gcn2p and Gcn4p | | orf_classification=Uncharacterized | | orf19.12421,IPF9623.2,IPF19458.2,Contig4-1464_0001,Contig4-1464_0002,Contig4-2724_0012,orf6.2660,orf6.52,CA1252,CaO19.4956,CAWG_00120 | |
| orf19.5021 | orf19.5021 | PDX1 | chr1 | 3039105 | 3040358 | | Pyruvate dehydrogenase complex protein X C an essential component of the mitochondrial pyruvate dehydrogenase complex which is involved in the respiratory pathway B protein present in exponential and stationary growth phase yeast cultures | |  | | orf19.12488,IPF22263.1,IPF3038.1,Contig4-3075_0007,orf6.7988,CA5242,CaO19.12488,CaO19.5021,CAWG_00069 | |
| orf19.3685 | orf19.3685 | PSY2 | chr1 | 478661 | 481603 | | Putative protein phosphatase PP4 complex subunit B macrophage-induced gene | |  | | orf19.11169,IPF18751.2,IPF13744.2,orf6.2494,orf6.3881,CA2114,CaO19.3685,IPF13744,CAWG_01155 | |
| orf19.2173 | orf19.2173 | MAF1 | chr2 | 1646206 | 1647533 | | Putative negative regulator of RNA polymerase III B decreased expression in hyphae compared to yeast-form cells B caspofungin repressed | | orf_classification=Uncharacterized | | orf19.9719,IPF25133.1,IPF10146.2,Contig4-3057_0024,orf6.6090,CA3745,CAWG_05903 | |
| orf19.2174 | orf19.2174 | RAD57 | chr2 | 1644238 | 1645773 | | Putative DNA recombination and repair protein B transcriptionally induced by interaction with macrophage B transcription is regulated by Nrg1p C Mig1p C and Tup1p B essential protein B S. cerevisiae ortholog is essential | | orf_classification=Uncharacterized | | orf19.9720,IPF25120.1,IPF10144.2,Contig4-3057_0023,orf6.6089,CA3744,CaO19.9720,CaO19.2174,CAWG_05902 | |
| orf19.218 | orf19.218 | BUD20 | chr2 | 1807304 | 1807645 | | Protein similar to S. cerevisiae Bud20p C which affects bud site selection B transposon mutation affects filamentous growth | |  | | orf19.7850,IPF27125.1,IPF18392.1,orf6.5155,CaO19.7850,CaO19.218,CAWG_05971 | |
| orf19.1513 | orf19.1513 | FAB1 | chr2 | 377966 | 385840 | | Phosphatidylinositol 3-phosphate 5-kinase B required for hyphal growth on solid media C and for wild-type vacuolar morphology and acidification B not required for wild-type virulence in mouse systemic infection or for adherence to HeLa cells | |  | | orf19.9088,IPF9288.2,IPF29418.1,Contig4-2754_0006,Contig4-2754_0007,Contig4-2754_0009,orf6.3964,CA2179,CaO19.1513,CAWG_03967 | |
| orf19.1555 | orf19.1555 | SAC3 | chr2 | 464538 | 468209 | | Putative nuclear pore-associated protein C required for small ribosomal subunit biogenesis B possibly an essential gene C disruptants not obtained by UAU1 method | | orf_classification=Uncharacterized | | orf19.9129,IPF7130.2,IPF29415.1,IPF18010.3,Contig4-2824_0019,Contig4-2824_0021,orf6.355,orf6.6413,CA6258,CA3982,orf19.1556,SAC3.3f,CAWG_04003 | |
| orf19.1586 | orf19.1586 | FGR22 | chr2 | 525603 | 526490 | | Putative phosphatidylinositol-specific phospholipase C 8PI-PLC 9 B predicted type 2 membrane protein B no S. cerevisiae ortholog B role in C and regulated by C filamentation C Hap43p B almost identical to orf19.5797 | |  | | PLC3,PLC2,PLC22,orf19.9159,IPF25798.1,IPF13130.2,Contig4-3005_0005,orf6.5334,CA3159,CaO19.9159,CaO19.1586,CAWG_04028 | |
| orf19.227 | orf19.227 | COX7 | chr3 | 526524 | 526853 | | Putative cytochrome c oxidase B flucytosine induced B repressed by nitric oxide | |  | | orf19.7857,IPF27081.1,IPF15250.2,Contig4-2074_0001,orf6.215,CA0031,CaO19.227,CaO19.7857,CAWG_02564 | |
| orf19.5430 | orf19.5430 | BUD21 | chr3 | 52683 | 53342 | | Small-subunit processome component B decreased expression in response to prostaglandins | |  | | orf19.12885,IPF21749.1,IPF1128.1,Contig4-2896_0013,orf6.8666,CA5783,CaO19.5430,CaO19.12885,IPF1128,CAWG_02372 | |
| orf19.736 | orf19.736 | SRB8 | chr4 | 1086617 | 1091860 | | Putative RNA polymerase II mediator complex subunit B early-stage biofilm-induced gene B ortholog of S. cerevisiae SRB8 | | orf_classification=Uncharacterized | | MED12,orf19.11252,orf19.8355,IPF23494.1,IPF13479.2,IPF26554.1,IPF16695.2,Contig4-1880_0002,orf6.526,orf6.2285,CA1018,CA0102,orf19.3768,IPF16695.5eoc,IPF16695,CAWG_03306 | |
| orf19.2906 | orf19.2906 | PGA41 | chr4 | 1402492 | 1403382 | | Putative GPI-anchored protein B adhesin-like protein | | orf_classification=Uncharacterized | | orf19.10424,IPF19507.2,IPF13070.2,Contig4-2946_0014,orf6.6116,orf6.259,CA3763,CaO19.2906,IPF13070,CAWG_03195 | |
| orf19.4697 | orf19.4697 | MDN1 | chr4 | 176061 | 191174 | | Putative midasin C a very large dynein-related AAA-type ATPase B induced during the mating process | | orf_classification=Uncharacterized | | orf19.12167,IPF22610.1,IPF11189.3,Contig4-1657_0002,Contig4-1657_0003,Contig4-2496_0003,orf6.1058,orf6.1684,orf6.1709,orf6.759,CA0283,CaO19.4697,IPF18034,IPF18034.2,CAWG_03685.1 | |
| orf19.2665 | orf19.2665 | MSN5 | chr4 | 682600 | 686337 | | Ortholog 8s 9 have importin-alpha export receptor activity C role in protein export from nucleus C tRNA re-export from nucleus and cytosol C nuclear envelope localization | | orf_classification=Uncharacterized | | orf19.10182,orf19.10183,IPF18929.1,IPF24579.1,IPF29005.1,IPF12426.2,Contig4-3083_0029,orf6.1628,orf6.1629,CA0601,CA0600,orf19.2666,MSN5.3f,CAWG_03476 | |
| orf19.3829 | orf19.3829 | PHR1 | chr4 | 970615 | 972261 | | Glycosidase of cell surface B may act on cell-wall beta-1 C3-glucan prior to beta-1 C6-glucan linkage B E169 and E270 at active site B role in systemic but not vaginal virulence 8neutral C not low pH 9 B high pH or filamentation induced | |  | | orf19.11310,orf6.7524,IPF23455.1,IPF15932.2,Contig4-2187_0001,Contig4-3060_0001,CA4857,CaO19.3829,CaO19.11310,CAWG_03358 | |
| orf19.3986 | orf19.3986 | PPR1 | chr5 | 1091736 | 1094318 | | Putative transcription factor with zinc cluster DNA-binding motif B has similarity to S. cerevisiae Ppr1p C which is a transcription factor involved in the regulation of uracil biosynthesis genes | |  | | orf19.11469,IPF19144.2,IPF9661.2,Contig4-2740_0001,orf6.7399,orf6.793,CA4758,CaO19.3986,CAWG_04863 | |
| orf19.4266 | orf19.4266 | SPR28 | chr5 | 566673 | 568047 | | Septin B similar to S. cerevisiae meiotic Fsporulation septin B mutant has no obvious phenotype B two introns with noncanonical branch site and 5 7 splice site C respectively B splicing inhibited upon exposure to alpha-factor | | orf_classification=Uncharacterized | | orf19.11742,orf19.11743,IPF23010.1,IPF22929.1,IPF23006.1,IPF9524.1,IPF9523.1,IPF9522.1,Contig4-2550_0005,Contig4-2550_0008,Contig4-2550_0009,orf6.3491,orf6.3490,CA1823,orf19.4267,CA1824,IPF9522.53f,CAWG_04636 | |
| orf19.4288 | orf19.4288 | CTA7 | chr5 | 607840 | 610344 | | Predicted zinc-finger protein B activates transcription in 1-hybrid assay in S. cerevisiae B has similarity to S. cerevisiae Stb4p | |  | | orf19.11764,IPF2963.1,IPF28450.1,Contig4-3019_0010,orf6.5198,CA3060,CaO19.4288,CaO19.11764,CAWG_04653 | |
| orf19.4318 | orf19.4318 | MIG1 | chr5 | 651946 | 653658 | | Transcriptional repressor B regulates genes for carbon source utilization B Tup1p-dependent C -independent functions B upregulated in biofilm and planktonic cultures B hyphal downregulated B Hap43p and caspofungin repressed | |  | | orf19.11793,IPF22963.1,IPF11048.2,Contig4-2700_0004,orf6.3170,CA1593,CaO19.11793,CaO19.4318,CAWG_04675 | |
| orf19.1108 | orf19.1108 | HAM1 | chr5 | 846299 | 846907 | | Putative deoxyribonucleoside triphosphate pyrophosphohydrolase B caspofungin repressed B regulated by Gcn2p and Gcn4p | | orf_classification=Uncharacterized | | orf19.8705,IPF26200.1,IPF6339.1,Contig4-2815_0019,orf6.2984,CA1460,CaO19.1108,CaO19.8705,CAWG_04758 | |
| orf19.5530 | orf19.5530 | NAB3 | chr6 | 529703 | 532213 | | Putative nuclear polyadenylated RNA-binding protein B flucytosine repressed | | orf_classification=Uncharacterized | | orf19.12976,IPF4338.2,IPF17959.3,Contig4-1760_0001,Contig4-1760_0002,orf6.1545,orf6.4471,orf6.452,CA0084,CaO19.5530,CAWG_05108 | |
| orf19.5551 | orf19.5551 | MIF2 | chr6 | 571522 | 573084 | | Centromere-associated protein B similar to CENP-C proteins B Cse4p and Mif2p colocalize at C. albicans centromeres | |  | | orf19.12997,IPF21589.1,IPF12632.1,Contig4-2462_0009,Contig4-2462_0010,orf6.4121,CA2284,CaO19.12997,CaO19.5551,CAWG_05073 | |
| orf19.6336 | orf19.6336 | PGA25 | chr6 | 7282 | 9900 | | Putative GPI-anchored adhesin-like protein B fluconazole-downregulated B induced in oralpharyngeal candidasis | | orf_classification=Uncharacterized | | orf19.7542,IPF5723.1,IPF5720.1,Contig4-3048_0011,orf6.5522,orf6.5529,CA3295,CaO19.6336,IPF5723,CA3296,IPF5723.exon2,IPF5723.exon1,CAWG_05350 | |
| orf19.4213 | orf19.4213 | FET31 | chr6 | 80231 | 82105 | | Putative iron transport multicopper oxidase precursor B flucytosine induced B caspofungin repressed | |  | | FET33,IPF17035.2,IPF11903.2,orf6.789,CA2922,CaO19.4213,orf6.5008,CAWG_05312 | |
| orf19.7030 | orf19.7030 | SSR1 | chr7 | 171720 | 172424 | | Beta-glucan associated ser Fthr rich cell-wall protein with a role in cell wall structure B GPI anchor B similar mRNA abundance in yeast-form and germ tubes B detected at germ tube plasma membrane B repressed in cells treated with Congo Red | |  | | CCW14,IPF3054.1,Contig4-2889_0002,orf6.7956,CA5213,CaJ7.0101,CaO19_7030,CaJ7_0101,CaO19.7030,CAWG_05443 | |
| orf19.3699 | orf19.3699 | TEP1 | chr7 | 565324 | 566484 | | Putative protein phosphatase of the PTP family 8tyrosine-specific 9 C similar to S. cerevisiae Tep1p | | orf_classification=Uncharacterized | | orf19.11183,IPF23574.1,IPF19913.1,Contig4-2159_0001,Contig4-2159_0002,orf6.2878,CA1398,CaJ7.0306,CaO19.11183,CaO19.3699,CaJ7_0306,CaO19_3699,IPF19913,CAWG_05604 | |
| orf19.5148 | orf19.5148 | CYR1 | chr7 | 662738 | 667810 | | Class III adenylyl cyclase B mutant lacks cAMP B mutant hyphal growth defect rescued by exogenous cAMP B required for mouse mucosal or systemic infection B role in macrophage sensitivity C apoptosis B downstream of Ras1p and CO2 signaling | |  | | CDC35,orf19.12615,orf19.12617,IPF12336.2,IPF12339.2,IPF12335.3,IPF28127.1,IPF28142.1,Contig4-3092_0006,Contig4-3092_0007,orf6.481,orf6.5946,orf6.5948,CA6295,CA6309,CA3625,CaJ7.0349,orf19.5150,orf19.5151,CYR1.5f,CAWG_05641 | |
| orf19.5505 | orf19.5505 | HIS7 | chr7 | 828024 | 829907 | | Putative imidazole glycerol phosphate synthase B histidine biosynthesis B no human Fmurine homolog B transcription induced by histidine starvation B regulated by Gcn2p and Gcn4p B higher protein level in stationary phase | | orf_classification=Uncharacterized | | IPF2583.2,Contig4-2718_0002,Contig4-2718_0003,orf6.4353,CA2447,CaJ7.0427,CaO19.5505,CaO19_5505,CaJ7_0427,CAWG_05703 | |
| orf19.651 | orf19.651 | LYP1 | chrR | 1084370 | 1085959 | | Putative permease B amphotericin B induced B flucytosine repressed B possibly an essential gene C disruptants not obtained by UAU1 method | | orf_classification=Uncharacterized | | orf19.8266,IPF19150.2,IPF16499.2,Contig4-2448_0003,orf6.3531,orf6.763,CA1853,CaO19.651,CAWG_01836 | |
| orf19.1816 | orf19.1816 | ALS3 | chrR | 1532346 | 1535813 | | ALS family cell wall adhesin B role in epithelial adhesion C endothelial invasiveness B allelic variation in adhesiveness B immunoprotective in mice B promotes biofilm formation by binding to SspB adhesin of S. gordonii B fluconazole-repressed | |  | | ALS8,ALS2,ALS10,orf19.9891,orf19.9379,IPF25511.1,IPF12561.3,IPF24952.1,IPF12997.2,orf6.2760,ALS3-1,CA0591,CA0448,orf19.2355,ALS3.5eoc,orf6.1614,IPF12561.2,CAWG_02005.1 | |
| orf19.2823 | orf19.2823 | RFG1 | chrR | 600293 | 602095 | | Transcriptional regulator of filamentous growth and hyphal genes B acts in Tup1p-dependent and -independent pathways B binds DNA B has HMG domain B not transcriptionally regulated by oxygen or serum B not responsible for hypoxic repression | |  | | ROX1,orf19.10341,orf6.1681,IPF14334.2,orf6.1166,orf6.1680,CA6270,CA0346,orf19.2824,RFG1.5f,CAWG_01604 | |
| orf19.2831 | orf19.2831 | RPC31 | chrR | 622663 | 623532 | | Putative RNA polymerase III subunit C31 B repressed by nitric oxide B induced during infection of murine kidney C compared to growth in vitro B has murine homolog | | orf_classification=Uncharacterized | | MDN1,orf19.10349,IPF14908.2,IPF15034.1,Contig4-2090_0003,orf6.4492,orf6.759,CA2546,CA0283,CaO19.10349,CaO19.2831,CA0024,RPC31.5eoc,IPF14908.1,CA0174,IPF11182.3eoc,IPF11182.2,CAWG_01611 | |
| orf19.473 | orf19.473 | TPO4 | chrR | 888821 | 890929 | | Putative sperimidine transporter B fungal-specific 8no human or murine homolog 9 | | orf_classification=Uncharacterized | | orf19.8104,IPF26767.1,IPF11142.2,Contig4-2684_0006,orf6.2283,CA1016,CaO19.8104,CaO19.473,IPF11142,CAWG_01729 | |
| orf19.6110 | orf19.6110 |  | chr1 | 11648 | 11995 | | Ortholog of Candida albicans WO-1 A CAWG_01361 | | orf_classification=Dubious | | orf19.13529,IPF21110.1,IPF10780.2,Contig4-1846_0001,orf6.6231,CaO19.13529,CaO19.6110,CAWG_01361 | |
| orf19.2476 | orf19.2476 |  | chr1 | 1198403 | 1203457 | | Ortholog 8s 9 have histone demethylase activity 8H3-trimethyl-K4 specific 9 activity | | orf_classification=Uncharacterized | | orf19.10013,IPF24871.1,IPF7201.2,Contig4-2471_0005,orf6.4959,orf6.6159,CA2884,CaO19.2476,IPF7201,CAWG_00831 | |
| orf19.2468 | orf19.2468 |  | chr1 | 1213982 | 1214902 | | Ortholog 8s 9 have trans-aconitate 3-methyltransferase activity and cytosol C nucleus localization | | orf_classification=Uncharacterized | | TMT1,orf19.10005,IPF6685.3,IPF13626.2,Contig4-2954_0008,orf6.250,orf6.4967,CA2890,CaO19.2468,IPF13626,CAWG_00824 | |
| orf19.2445 | orf19.2445 |  | chr1 | 1261730 | 1263376 | | Putative dicarboxylic amino acid permease B fungal-specific 8no human or murine homolog 9 B transcription is induced in response to alpha pheromone in SpiderM medium | | orf_classification=Uncharacterized | | DIP51,DIP5,DIP52,orf19.9981,IPF24835.1,IPF7474.3,Contig4-2783_0004,orf6.1918,orf6.3623,CA1922,CaO19.2445,IPF7474.2,CAWG_00807 | |
| orf19.2797 | orf19.2797 |  | chr1 | 1619388 | 1621259 | | Has domain 8s 9 with predicted ATP binding C DNA binding C helicase activity | | orf_classification=Uncharacterized | | orf19.10315,IPF24549.1,IPF11802.2,Contig4-2693_0005,orf6.3726,CA2005,CaO19.2797,CaO19.10315,IPF11802,CAWG_00670 | |
| orf19.2788 | orf19.2788 |  | chr1 | 1637927 | 1639135 | | Has domain 8s 9 with predicted RNA binding C pseudouridine synthase activity and role in pseudouridine synthesis | | orf_classification=Uncharacterized | | RIB21,orf19.10304,IPF24552.1,IPF7263.2,Contig4-2384_0005,Contig4-2384_0007,orf6.3605,orf6.976,CA0247,CaO19.2788,CAWG_00662 | |
| orf19.2786 | orf19.2786 |  | chr1 | 1642067 | 1645192 | | Has domain 8s 9 with predicted protein transporter activity C role in intracellular protein transport C vesicle-mediated transport and clathrin adaptor complex localization | | orf_classification=Uncharacterized | | APL3,orf19.10302,IPF24565.1,IPF7268.2,Contig4-2533_0009,orf6.3603,CA1908,CaO19.2786,CaO19.10302,CAWG_00660 | |
| orf19.5057 | orf19.5057 |  | chr1 | 1710213 | 1711466 | | Ortholog of Candida dubliniensis CD36 A CD36_07380 and Candida albicans WO-1 A CAWG_00634 | | orf_classification=Uncharacterized | | orf19.12524,IPF19487.2,IPF12213.2,Contig4-2407_0007,orf6.16,orf6.3379,CA1741,CaO19.5057,IPF12213,CAWG_00634 | |
| orf19.4846 | orf19.4846 |  | chr1 | 2148256 | 2149497 | | GlcNAc-induced protein | | orf_classification=Uncharacterized | | orf19.12309,IPF4500,IPF22439.1,IPF4500.1,Contig4-3078_0008,orf6.7365,CA4738,CaO19.12309,CaO19.4846,CAWG_00449 | |
| orf19.4908 | orf19.4908 |  | chr1 | 2295578 | 2295913 | | Ortholog of Candida dubliniensis CD36 A CD36_09765 and Candida albicans WO-1 A CAWG_00396 | | orf_classification=Dubious | | orf19.12374,IPF22515.1,IPF9610.2,Contig4-2018_0002,orf6.3755,CaO19.12374,CaO19.4908,CAWG_00396 | |
| orf19.3307 | orf19.3307 |  | chr1 | 241122 | 242513 | | Ortholog 8s 9 have N CN-dimethylaniline monooxygenase activity C role in protein folding and endoplasmic reticulum membrane C mitochondrion localization | | orf_classification=Uncharacterized | | orf19.10817,IPF23937.1,IPF5761.2,Contig4-2978_0001,orf6.7202,CA4616,CaO19.3307,CaO19.10817,IPF5761,CAWG_01256 | |
| orf19.660 | orf19.660 |  | chr1 | 2506221 | 2508167 | | Putative protein of unknown function B mRNA binds to She3p | | orf_classification=Uncharacterized | | orf19.8276,IPF26622.1,IPF4002.2,Contig4-2788_0013,orf6.6553,CA4095,CaO19.660,CaO19.8276,IPF4002,CAWG_00293 | |
| orf19.3320 | orf19.3320 |  | chr1 | 259001 | 259354 | | Ortholog of Candida albicans WO-1 A CAWG_01245 | | orf_classification=Dubious | | orf19.10830,IPF23974.1,IPF28816.1,Contig4-2978_0017,orf6.7215,CaO19.10830,CaO19.3320,CAWG_01245 | |
| orf19.5221 | orf19.5221 |  | chr1 | 2704703 | 2707654 | | Ortholog 8s 9 have phosphatidylinositol-4 C5-bisphosphate binding activity | | orf_classification=Uncharacterized | | orf19.12688,IPF19391.2,IPF10863.2,orf6.3682,orf6.210,orf6.3683,CA1969,CA1970,orf19.5222,IPF18579.3f,CAWG_00198 | |
| orf19.6353 | orf19.6353 |  | chr1 | 2767835 | 2769913 | | Ortholog of Candida albicans WO-1 A CAWG_00172 | | orf_classification=Uncharacterized | | LPF40,IFA5,orf19.13710,IPF20797.1,IPF11374.2,orf6.5782,CA3506,CaO19.6353,CaO19.13710,CAWG_00172 | |
| orf19.7223 | orf19.7223 |  | chr1 | 3121139 | 3122893 | | Ortholog 8s 9 have protein-lysine N-methyltransferase activity C role in peptidyl-lysine monomethylation and cytosol C nucleus localization | | orf_classification=Uncharacterized | | IPF863.1,Contig4-3043_0010,orf6.8123,CA5348,CaO19.7223,IPF863,CAWG_00038 | |
| orf19.7271 | orf19.7271 |  | chr1 | 3181309 | 3181629 | | Predicted ORF in Assemblies 19 C 20 and 21 B transcription detected in high-resolution tiling array experiments | | orf_classification=Uncharacterized | | IPF5214.1,Contig4-2986_0030,orf6.8171,CaO19.7271,CAWG_00003 | |
| orf19.6084 | orf19.6084 |  | chr1 | 32680 | 33318 | | Biofilm-induced gene | | orf_classification=Uncharacterized | | orf19.13503,IPF21095.1,IPF4608.1,Contig4-2998_0016,orf6.6257,CA3865,CaO19.6084,CaO19.13503,IPF4608,CAWG_01346 | |
| orf19.4538 | orf19.4538 |  | chr1 | 382776 | 383606 | | Ortholog 8s 9 have mRNA binding C protein heterodimerization activity C role in mRNA cleavage C mRNA polyadenylation and mRNA cleavage factor complex localization | | orf_classification=Uncharacterized | | RNA15,orf19.12013,IPF22721.1,IPF8057.2,Contig4-2727_0003,orf6.6684,CA4197,CaO19.12013,CaO19.4538,IPF8057,CAWG_01195 | |
| orf19.6075 | orf19.6075 |  | chr1 | 49296 | 49844 | | Hap43p-repressed gene B ortholog of S. cerevisiae CDC36 C a CCR4-NOT complex component | | orf_classification=Uncharacterized | | CDC36,orf19.13496,IPF21103.1,IPF8371.1,Contig4-2396_0006,orf6.7619,CA4934,CaO19.13496,CaO19.6075,CAWG_01339 | |
| orf19.2959 | orf19.2959 |  | chr1 | 562417 | 562719 | | Predicted ORF from Assembly 19 B removed from Assembly 20 B subsequently reinstated in Assembly 21 based on comparative genome analysis | | orf_classification=Uncharacterized | | orf19.10476,orf6.6657,CaO19.2959,CaO19.10476,CAWG_01114 | |
| orf19.4472 | orf19.4472 |  | chr1 | 837520 | 837837 | | Ortholog of Candida albicans WO-1 A CAWG_00996 | | orf_classification=Dubious | | orf19.11952,IPF22785.1,IPF28386.1,orf6.4315,CaO19.4472,CaO19.11952,CAWG_00996 | |
| orf19.3565 | orf19.3565 |  | chr2 | 1099547 | 1100407 | | Ortholog of C. parapsilosis CDC317 A CPAR2_206470 C Candida tenuis NRRL Y-1498 A CANTEDRAFT_113899 C Debaryomyces hansenii CBS767 A DEHA2C05676g and Candida dubliniensis CD36 A CD36_19830 | | orf_classification=Uncharacterized | | orf19.11049,IPF23737.1,IPF3192.1,Contig4-3023_0022,orf6.5764,CA3490,CaO19.11049,CaO19.3565,IPF3192,CAWG_04285 | |
| orf19.5210 | orf19.5210 |  | chr2 | 1198925 | 1200658 | | Hap43p-repressed gene B possibly an essential gene C disruptants not obtained by UAU1 method | | orf_classification=Uncharacterized | | XBP1,orf19.12677,IPF22005.1,IPF11259.2,Contig4-3052_0003,orf6.3453,CA1796,CaO19.5210,CaO19.12677,IPF11259,CAWG_04332 | |
| orf19.1296 | orf19.1296 |  | chr2 | 1354671 | 1356380 | | Ortholog 8s 9 have role in spliceosomal snRNP assembly and U4 FU6 x U5 tri-snRNP complex C mitochondrion localization | | orf_classification=Uncharacterized | | PRP31,orf19.8876,IPF26039.1,IPF13982.2,orf6.922,CA0224,orf19.82,CaO19.8876,CaO19.1296,CAWG_04399 | |
| orf19.2040 | orf19.2040 |  | chr2 | 142787 | 144556 | | Ortholog 8s 9 have oxidoreductase activity C acting on NADH or NADPH activity C role in iron-sulfur cluster assembly C oxidation-reduction process and mitochondrion localization | | orf_classification=Uncharacterized | | TAH18,orf19.9588,IPF25245.1,IPF4132.2,Contig4-2863_0024,orf6.5979,CA3649,CaO19.9588,CaO19.2040,IPF4132,CAWG_03863 | |
| orf19.2258 | orf19.2258 |  | chr2 | 1438852 | 1440036 | | Ortholog of C. parapsilosis CDC317 A CPAR2_406650 C Candida tenuis NRRL Y-1498 A CANTEDRAFT_130046 C Debaryomyces hansenii CBS767 A DEHA2F24134g and Candida dubliniensis CD36 A CD36_21310 | | orf_classification=Uncharacterized | | orf19.9798,IPF25059.1,IPF15466.2,Contig4-2665_0001,orf6.5888,CA3582,CaO19.9798,CaO19.2258,IPF15466,CAWG_05800 | |
| orf19.2025 | orf19.2025 |  | chr2 | 161361 | 161705 | | Ortholog of Candida albicans WO-1 A CAWG_03877 | | orf_classification=Dubious | | IPF29232.1,orf6.470,CaO19.2025,CAWG_03877 | |
| orf19.2181 | orf19.2181 |  | chr2 | 1632310 | 1632840 | | ORF Predicted by Annotation Working Group B overlaps orf19.2180 | | orf_classification=Uncharacterized | | IPF25147.1,IPF5525.1,orf6.6082,CaO19.2181,CaO19.9727,CAWG_05895 | |
| orf19.3604 | orf19.3604 |  | chr2 | 1772272 | 1773420 | | Ortholog 8s 9 have nucleus localization | | orf_classification=Uncharacterized | | orf19.11087,IPF18810.1,IPF17166.2,orf6.2460,orf6.2223,CA0977,CaO19.3604,IPF18810,CAWG_05956 | |
| orf19.4068 | orf19.4068 |  | chr2 | 1860281 | 1862569 | | Biofilm-induced gene | | orf_classification=Uncharacterized | | orf19.11551,IPF17322.2,IPF9462.2,orf6.2486,orf6.604,CA1140,CaO19.4068,IPF17322.3f,CAWG_05991 | |
| orf19.1456 | orf19.1456 |  | chr2 | 270261 | 270695 | | Ortholog of Candida albicans WO-1 A CAWG_03922 | | orf_classification=Uncharacterized | | IPF25833.1,IPF29424.1,orf6.7117,CaO19.1456,CaO19.9031,CAWG_03922 | |
| orf19.1536 | orf19.1536 |  | chr2 | 433195 | 434631 | | Putative vacuolar transporter B Hap43p-induced gene C required for normal filamentous growth B mRNA binds to She3p and is localized to hyphal tips | |  | | orf19.9111,IPF19060.1,Contig4-2088_0003,orf6.1061,orf6.1062,orf6.2308,CA6257,CA1030,orf19.1537,ZRC.5f,CAWG_03987.1 | |
| orf19.1548 | orf19.1548 |  | chr2 | 459139 | 459870 | | Ortholog 8s 9 have role in mRNA splicing C via spliceosome and U4 FU6 x U5 tri-snRNP complex localization | | orf_classification=Uncharacterized | | SNU23,orf19.9122,IPF25806.1,IPF15310.2,orf6.6406,CA3976,CaO19.1548,CaO19.9122,CAWG_03998 | |
| orf19.1574 | orf19.1574 |  | chr2 | 501240 | 503006 | | Predicted ORF in Assemblies 19 C 20 and 21 B possibly an essential gene C disruptants not obtained by UAU1 method | | orf_classification=Uncharacterized | | TAF7,TAF67,orf19.9147,IPF15686.2,IPF29407.1,Contig4-2925_0020,orf6.1886,CA0762,CaO19.1574,CAWG_04018 | |
| orf19.855 | orf19.855 |  | chr2 | 768528 | 769277 | | Ortholog of Candida tenuis NRRL Y-1498 A CANTEDRAFT_124631 C Debaryomyces hansenii CBS767 A DEHA2D04356g C Candida dubliniensis CD36 A CD36_18340 and Pichia stipitis Pignal A PICST_34839 | | orf_classification=Uncharacterized | | orf19.8475,IPF26450.1,IPF11974.1,Contig4-2541_0010,orf6.1904,CA0774,CaO19.855,IPF11974,CAWG_04128 | |
| orf19.2074 | orf19.2074 |  | chr2 | 84527 | 84835 | | Ortholog of Candida albicans WO-1 A CAWG_03833 | | orf_classification=Dubious | | orf19.9621,IPF25336.1,IPF29235.1,orf6.7481,CaO19.2074,CaO19.9621,CAWG_03833 | |
| orf19.5966 | orf19.5966 |  | chr3 | 1072430 | 1072771 | | Ortholog of Candida albicans WO-1 A CAWG_02810 | | orf_classification=Dubious | | orf19.13387,IPF21294.1,IPF27869.1,orf6.9071,CaO19.5966,CaO19.13387,CAWG_02810 | |
| orf19.5391 | orf19.5391 |  | chr3 | 109085 | 112744 | | Hap43p-induced gene | | orf_classification=Uncharacterized | | SAP130,orf19.12846,IPF21794.1,IPF1063.2,Contig4-3071_0027,Contig4-3071_0028,orf6.8627,CA5755,CaO19.5391,CaO19.12846,IPF1063,CAWG_02402 | |
| orf19.7441 | orf19.7441 |  | chr3 | 1490992 | 1491918 | | Ortholog 8s 9 have role in cellular lipid metabolic process C mRNA export from nucleus C protein export from nucleus and nuclear envelope localization | | orf_classification=Uncharacterized | | IPF2870.2,Contig4-2076_0005,orf6.8601,CA5731,CaO19.7441,IPF2870,CAWG_02962 | |
| orf19.6164 | orf19.6164 |  | chr3 | 174381 | 174707 | | Ortholog of Candida albicans WO-1 A CAWG_02424 | | orf_classification=Dubious | | IPF27451.1,Contig4-2276_0003,orf6.5325,CaO19.6164,CAWG_02424 | |
| orf19.6179 | orf19.6179 |  | chr3 | 1776231 | 1776923 | | ORF Predicted by Annotation Working Group B overlaps FBP1 Forf19.6178 | | orf_classification=Uncharacterized | | IPF3434.1,orf6.5400,CAWG_03090 | |
| orf19.2512 | orf19.2512 |  | chr3 | 222909 | 223568 | | Ortholog of C. parapsilosis CDC317 A CPAR2_103050 C Candida tenuis NRRL Y-1498 A CANTEDRAFT_116326 C Debaryomyces hansenii CBS767 A DEHA2G01958g and Candida dubliniensis CD36 A CD36_81000 | | orf_classification=Uncharacterized | | orf19.10048,IPF12803.2,IPF29085.1,Contig4-1978_0003,orf6.3917,CA2143,CaO19.2512,CaO19.10048,IPF12803,CAWG_02444 | |
| orf19.3156 | orf19.3156 |  | chr3 | 240982 | 244245 | | Predicted ORF in Assemblies 19 C 20 and 21 B transcriptionally activated by Mnl1p under weak acid stress | | orf_classification=Uncharacterized | | MDS1,orf19.10665,IPF13504.2,IPF310.3,Contig4-2595_0003,Contig4-2595_0004,Contig4-2595_0005,orf6.1663,orf6.2693,CA1270,CaO19.3156,IPF13504,CAWG_02452 | |
| orf19.257 | orf19.257 |  | chr3 | 572936 | 573262 | | Transcription is negatively regulated by Sfu1p B repressed by nitric oxide | | orf_classification=Dubious | | orf19.7889,orf6.694,orf6.1190,IPF27080.1,IPF29899.1,CaO19.257,CAWG_02588 | |
| orf19.5472 | orf19.5472 |  | chr3 | 5971 | 7002 | | ORF Predicted by Annotation Working Group B overlaps orf19.5469 | | orf_classification=Dubious | | IPF20006.2,IPF2686.1,orf6.6374,orf6.8708 | |
| orf19.5894 | orf19.5894 |  | chr3 | 923827 | 925531 | | Ortholog of S. cerevisiae A YEL023C C C. glabrata CBS138 A CAGL0I03168g C Candida tenuis NRRL Y-1498 A CANTEDRAFT_101635 C Candida dubliniensis CD36 A CD36_84380 and Pichia stipitis Pignal A PICST_19384 | | orf_classification=Uncharacterized | | orf19.13315,orf19.13316,IPF21290.1,IPF21225.1,IPF1862.1,IPF1861.2,Contig4-3084_0047,Contig4-3084_0048,orf6.9143,orf6.9142,CA6148,CA6147,orf19.5895,IPF1862.3f,CAWG_02756 | |
| orf19.4170 | orf19.4170 |  | chr4 | 124050 | 124874 | | Hap43p-repressed gene | | orf_classification=Uncharacterized | | CIS309,IFL3,orf19.11646,IPF6188.1,IPF28505.1,Contig4-2878_0006,orf6.6064,CA3720,CaO19.11646,CaO19.4170,CAWG_03709 | |
| orf19.1257 | orf19.1257 |  | chr4 | 1262779 | 1263111 | | Ortholog of Candida albicans WO-1 A CAWG_03251 | | orf_classification=Dubious | | IPF29533.1,orf6.5724,CaO19.1257,CAWG_03251 | |
| orf19.3113 | orf19.3113 |  | chr4 | 1544911 | 1545339 | | Ortholog of Debaryomyces hansenii CBS767 A DEHA2B15840g C Candida dubliniensis CD36 A CD36_46430 C Pichia stipitis Pignal A PICST_37097 and Candida tropicalis MYA-3404 A CTRG_03751 | | orf_classification=Uncharacterized | | orf19.10625,IPF24211.1,IPF14368.2,Contig4-2739_0004,orf6.6932,CaO19.3113,CaO19.10625,CAWG_03132 | |
| orf19.4702 | orf19.4702 |  | chr4 | 166705 | 167607 | | Biofilm-induced gene B expression regulated during planktonic growth B similarity to mutator-like element 8MULE 9 transposase | | orf_classification=Uncharacterized | | orf19.12172,IPF14519.1,IPF16377.2,Contig4-2960_0008,orf6.3519,orf6.1755,CA0683,CaO19.4702,IPF14519.5f,IPF14519,CAWG_03689 | |
| orf19.4690 | orf19.4690 |  | chr4 | 195209 | 196942 | | Biofilm-induced gene B induced by nitric oxide independent of Yhb1p | | orf_classification=Uncharacterized | | SMF11,SMF12,orf19.12159,IPF17437.2,IPF14122.2,Contig4-2733_0001,orf6.1056,orf6.1770,orf6.460,CA0087,CaO19.4690,CAWG_03681 | |
| orf19.4685 | orf19.4685 |  | chr4 | 206797 | 207114 | | Ortholog of Candida albicans WO-1 A CAWG_03677 | | orf_classification=Dubious | | orf19.12154,IPF22669.1,IPF28327.1,Contig4-2733_0016,orf6.5451,CaO19.4685,CaO19.12154,CAWG_03677 | |
| orf19.4657 | orf19.4657 |  | chr4 | 263911 | 265413 | | Ortholog 8s 9 have phosphoprotein phosphatase activity C role in negative regulation of phospholipid biosynthetic process C nuclear envelope organization and Nem1-Spo7 phosphatase complex C integral to membrane C mitochondrion localization | | orf_classification=Uncharacterized | | NEM1,orf19.12127,IPF22586.1,IPF12196.2,orf6.4708,CA2705,CaO19.4657,CaO19.12127,CAWG_03654 | |
| orf19.4643 | orf19.4643 |  | chr4 | 294807 | 301958 | | Ortholog of Candida dubliniensis CD36 A CD36_41430 C Candida tropicalis MYA-3404 A CTRG_00187 and Candida albicans WO-1 A CAWG_03642 | | orf_classification=Uncharacterized | | orf19.12113,IPF19720.2,IPF28308.1,Contig4-2362_0003,Contig4-2596_0007,Contig4-2596_0008,orf6.1491,orf6.2365,CA1061,CaO19.4643,IPF19720,IPF19720.3eoc,IPF19720.1,CAWG_03642 | |
| orf19.3362 | orf19.3362 |  | chr4 | 702777 | 704120 | | Has domain 8s 9 with predicted flavin adenine dinucleotide binding C oxidoreductase activity and role in oxidation-reduction process | | orf_classification=Uncharacterized | | orf19.10870,IPF23891.1,IPF15758.2,Contig4-2353_0001,orf6.1783,CaO19.10870,CaO19.3362,CAWG_03481 | |
| orf19.5290 | orf19.5290 |  | chr4 | 897198 | 898541 | | Hap43p-induced gene B transcription is negatively regulated by Sfu1p | | orf_classification=Uncharacterized | | orf19.12748,orf6.3944,IPF21848.1,IPF17022.3,CaO19.5290,CaO19.12748 | |
| orf19.1430 | orf19.1430 |  | chr4 | 897198 | 899744 | | Ortholog of C. parapsilosis CDC317 A CPAR2_402120 C Lodderomyces elongisporus NRLL YB-4239 A LELG_04437 C Candida dubliniensis CD36 A CD36_43870 and Candida orthopsilosis Co 90-125 A CORT0E02170 | | orf_classification=Uncharacterized | | orf19.12773,orf19.9006,IPF25911.1,IPF9379.3,IPF19810.1,IPF28064.1,Contig4-2598_0008,Contig4-2598_0009,orf6.6399,orf6.8302,CA3969,CA5481,orf19.5313,IPF9379.5eoc,IPF9379,IPF9379.2,CAWG_03407 | |
| orf19.4187 | orf19.4187 |  | chr4 | 97574 | 98893 | | Ortholog 8s 9 have role in mitochondrial genome maintenance C phospholipid transport C protein import into mitochondrial outer membrane and ERMES complex C integral to endoplasmic reticulum membrane localization | | orf_classification=Uncharacterized | | MMM1,orf19.11664,IPF23092.1,IPF8006.2,Contig4-2846_0008,orf6.3644,CA1937,CaO19.4187,CaO19.11664,CAWG_03718 | |
| orf19.4055 | orf19.4055 |  | chr5 | 1188806 | 1189117 | | Protein similar to S. cerevisiae Ybr075wp B transposon mutation affects filamentous growth B clade-associated gene expression | |  | | orf19.11537,IPF23360.1,IPF3468.2,Contig4-2210_0004,orf6.4421,YBR075W,CA2496,CaO19.4055,CaO19.11537,IPF3468,CAWG_04916 | |
| orf19.3196 | orf19.3196 |  | chr5 | 402485 | 402787 | | Ortholog of Candida albicans WO-1 A CAWG_04564 | | orf_classification=Dubious | | IPF24136.1,IPF28871.1,orf6.1879,CaO19.3196,CAWG_04564 | |
| orf19.4245 | orf19.4245 |  | chr5 | 525711 | 527057 | | Hap43p-repressed gene | | orf_classification=Uncharacterized | | orf19.11720,IPF20009.1,IPF19942.1,Contig4-1884_0003,Contig4-1884_0004,orf6.3979,orf6.6600,CA4130,CaO19.4245,IPF20009,CAWG_04620 | |
| orf19.4270 | orf19.4270 |  | chr5 | 573196 | 575493 | | Putative mannosyltransferase B regulated by Sef1p- C Sfu1p- C and Hap43p | | orf_classification=Uncharacterized | | orf19.11746,IPF22942.1,IPF9529.2,orf6.3487,CA1820,CaO19.11746,CaO19.4270,IPF9529,CAWG_04639 | |
| orf19.4273 | orf19.4273 |  | chr5 | 576587 | 579346 | | Hap43p-induced gene | | orf_classification=Uncharacterized | | orf19.11749,IPF22931.1,IPF16549.2,Contig4-2872_0001,orf6.3484,CA1819,CaO19.11749,CaO19.4273,IPF16549,CAWG_04641 | |
| orf19.4305 | orf19.4305 |  | chr5 | 628397 | 629119 | | Ortholog of C. parapsilosis CDC317 A CPAR2_100565 C Debaryomyces hansenii CBS767 A DEHA2G06908g C Candida dubliniensis CD36 A CD36_52590 and Candida guilliermondii ATCC 6260 A PGUG_02858 | | orf_classification=Uncharacterized | | orf19.11781,IPF23000.1,IPF2932.1,Contig4-2902_0001,orf6.4610,CA2639,CaO19.11781,CaO19.4305,IPF2932,CAWG_04663 | |
| orf19.1123 | orf19.1123 |  | chr5 | 823514 | 823867 | | Ortholog of Candida albicans WO-1 A CAWG_04744 | | orf_classification=Dubious | | orf19.8721,IPF26211.1,IPF29575.1,orf6.1198,CaO19.8721,CaO19.1123,CAWG_04744 | |
| orf19.3214 | orf19.3214 |  | chr5 | 873713 | 875197 | | Membrane-localized protein | |  | | AES7,IFJ6,orf19.10726,IPF24098.1,IPF12814.2,Contig4-2443_0006,orf6.3196,CA1612,CaO19.10726,CaO19.3214,CAWG_04769 | |
| orf19.133 | orf19.133 |  | chr6 | 242064 | 242387 | | Protein of unknown function B induced by nitric oxide independent of Yhb1p | | orf_classification=Dubious | | IPF29964.1,Contig4-2900_0011,orf6.3481,CaO19.133 | |
| orf19.134 | orf19.134 |  | chr6 | 244274 | 244684 | | Predicted ORF in Assemblies 19 C 20 and 21 B ciclopirox olamine induced | | orf_classification=Dubious | | IPF7023.2,Contig4-2900_0005,orf6.3480,CA1818,CaO19.134,IPF7023.3f,IPF7023.3 | |
| orf19.5620 | orf19.5620 |  | chr6 | 696408 | 697337 | | Stationary phase enriched protein B Gcn4p-regulated B induced by amino acid starvation 83-AT 9 B increased transcription by benomyl treatment or in an azole-resistant strain that overexpresses MDR1 B biofilm-induced B overlaps orf19.5621 | | orf_classification=Uncharacterized | | PHZ1,orf19.13065,IPF18418.1,IPF12436.3,Contig4-2504_0003,orf6.212,orf6.4779,CA2756,CaO19.5620,IPF18418,CAWG_05038 | |
| orf19.5775 | orf19.5775 |  | chr6 | 863241 | 864779 | | Predicted ORF in Assemblies 19 C 20 and 21 B member of a family encoded by FGR6-related genes in the RB2 repeat sequence | | orf_classification=Uncharacterized | | IFV7,orf19.13197,IPF21457.1,IPF20146.1,Contig4-2914_0008,orf6.6183,CA3817,CaO19.5775,CaO19.13197,CAWG_04977 | |
| orf19.1087 | orf19.1087 |  | chr6 | 950736 | 953156 | | Ortholog of C. parapsilosis CDC317 A CPAR2_601130 C Lodderomyces elongisporus NRLL YB-4239 A LELG_04885 C Candida dubliniensis CD36 A CD36_64620 and Candida orthopsilosis Co 90-125 A CORT0F02130 | | orf_classification=Uncharacterized | | orf19.8688,IPF19579.1,IPF19578.1,orf6.1224,orf6.350,CA0371,CaO19.1087,IPF19578,CAWG_04949 | |
| orf19.7027 | orf19.7027 |  | chr7 | 176562 | 179318 | | Ortholog of C. parapsilosis CDC317 A CPAR2_301430 C Candida tenuis NRRL Y-1498 A CANTEDRAFT_132919 C Debaryomyces hansenii CBS767 A DEHA2F22528g and Candida dubliniensis CD36 A CD36_70820 | | orf_classification=Uncharacterized | | IPF2373.2,IPF18125.1,orf6.7953,orf6.7954,CA5210,CA5211,CaJ7.0104,CaJ7.0105,orf19.7028,IPF2373 | |
| orf19.6911 | orf19.6911 |  | chr7 | 264746 | 265075 | | Ortholog of Candida albicans WO-1 A CAWG_05482 | | orf_classification=Dubious | | IPF27390.1,orf6.7175,CaJ7.0145,CaO19_6911,CaJ7_0145,CaO19.6911,CAWG_05482 | |
| orf19.6919 | orf19.6919 |  | chr7 | 281377 | 282504 | | Ortholog of C. parapsilosis CDC317 A CPAR2_702710 C Candida tenuis NRRL Y-1498 A CANTEDRAFT_116256 C Debaryomyces hansenii CBS767 A DEHA2E17732g and Candida dubliniensis CD36 A CD36_71210 | | orf_classification=Uncharacterized | | IPF2199.2,orf6.7183,CA4600,CaJ7.0153,CaJ7_0153,CaO19_6919,CaO19.6919,IPF2199,CAWG_05489 | |
| orf19.6557 | orf19.6557 |  | chr7 | 359158 | 360897 | | Predicted ORF in Assemblies 19 C 20 and 21 B transcriptionally activated by Mnl1p under weak acid stress | | orf_classification=Uncharacterized | | AMD23,orf19.13910,IPF20579.1,IPF1680.2,orf6.7545,CA4871,CaJ7.0188,CaJ7_0188,CaO19_6557,CaO19.13910,CaO19.6557,IPF1680,CAWG_05518 | |
| orf19.6499 | orf19.6499 |  | chr7 | 472190 | 475846 | | Has domain 8s 9 with predicted DNA binding C DNA-directed RNA polymerase activity and role in transcription C DNA-dependent | | orf_classification=Uncharacterized | | orf19.13852,IPF20679.1,IPF8437.2,Contig4-2822_0008,orf6.3364,CA1731,CaJ7.0251,CaO19.13852,CaJ7_0251,CaO19_6499,CaO19.6499,IPF8437,CAWG_05566 | |
| orf19.6488 | orf19.6488 |  | chr7 | 489299 | 491008 | | Ortholog of C. parapsilosis CDC317 A CPAR2_301140 C Candida tenuis NRRL Y-1498 A CANTEDRAFT_135055 C Debaryomyces hansenii CBS767 A DEHA2E07678g and Candida dubliniensis CD36 A CD36_72050 | | orf_classification=Uncharacterized | | orf19.13841,IPF19855.1,IPF19854.1,Contig4-2725_0013,Contig4-2725_0014,orf6.1018,orf6.1073,CA0292,CaJ7.0262,CaJ7_0262,CaO19_6488,CaO19.6488,IPF19855,CAWG_05574 | |
| orf19.5190 | orf19.5190 |  | chr7 | 590363 | 590788 | | Ortholog of Candida albicans WO-1 A CAWG_05610 | | orf_classification=Dubious | | orf19.12657,IPF22082.1,IPF17727.1,Contig4-1794_0007,Contig4-2965_0002,Contig4-3084_0003,orf6.3501,CA1832,CaJ7.0312,CaO19.12657,CaO19.5190,IPF17727,CAWG_05610 | |
| orf19.1330 | orf19.1330 |  | chr7 | 762348 | 764168 | | Ortholog of Candida albicans WO-1 A CAWG_05678 | | orf_classification=Uncharacterized | | LPF15,IFB3,orf19.8910,IPF25994.1,IPF15703.1,orf6.4357,orf6.959,orf6.6812,CA0240,CaJ7.0397,CaO19.8910,CaO19.1330,CAWG_05678 | |
| orf19.7173 | orf19.7173 |  | chr7 | 881593 | 883314 | | Ortholog of C. parapsilosis CDC317 A CPAR2_702310 C Lodderomyces elongisporus NRLL YB-4239 A LELG_04029 C Candida dubliniensis CD36 A CD36_73730 and Candida orthopsilosis Co 90-125 A CORT0G02490 | | orf_classification=Uncharacterized | | IPF2178.1,Contig4-3097_0018,orf6.8073,CA5304,CaJ7.0466,CaJ7_0466,CaO19_7173,CaO19.7173,IPF2178,CAWG_05735 | |
| orf19.7164 | orf19.7164 |  | chr7 | 890363 | 891457 | | Ortholog 8s 9 have role in vacuolar protein processing and Golgi apparatus C endoplasmic reticulum C fungal-type vacuole membrane localization | | orf_classification=Uncharacterized | | IPF2190.1,Contig4-3097_0002,orf6.8064,CA5299,CaJ7.0475,CaJ7_0475,CaO19_7164,CaO19.7164,IPF2190,CAWG_05740 | |
| orf19.1008 | orf19.1008 |  | chrR | 1136826 | 1137140 | | Ortholog of Candida albicans WO-1 A CAWG_01856 | | orf_classification=Dubious | | IPF29604.1,Contig4-3028_0025,orf6.612,CaO19.1008,CAWG_01856 | |
| orf19.3512 | orf19.3512 |  | chrR | 1160503 | 1161567 | | Ortholog of Candida dubliniensis CD36 A CD36_30750 and Candida albicans WO-1 A CAWG_01867 | | orf_classification=Uncharacterized | | CIS310,IFL4,IPF12574.2,IPF27468.1,Contig4-2428_0001,orf6.1945,CA0803,orf19.5283,CaO19.3512,CAWG_01867 | |
| orf19.6641 | orf19.6641 |  | chrR | 1211934 | 1212791 | | Ortholog of Candida albicans WO-1 A CAWG_01892 | | orf_classification=Uncharacterized | | LPF41,orf19.13962,IPF20520.1,IPF5015.2,Contig4-3035_0006,orf6.6538,CA4085,CaO19.13962,CaO19.6641,IPF5015,CAWG_01892 | |
| orf19.3886 | orf19.3886 |  | chrR | 1340001 | 1341404 | | Ortholog of C. parapsilosis CDC317 A CPAR2_205050 C Debaryomyces hansenii CBS767 A DEHA2G10736g C Candida dubliniensis CD36 A CD36_31820 and Pichia stipitis Pignal A PICST_32993 | | orf_classification=Uncharacterized | | orf19.11367,IPF23392.1,IPF7763.2,Contig4-2958_0019,orf6.5857,CA3557,CaO19.11367,CaO19.3886,IPF7763,CAWG_01941 | |
| orf19.7343 | orf19.7343 |  | chrR | 2021942 | 2023318 | | Ortholog 8s 9 have role in mRNA splicing C via spliceosome and U4 FU6 x U5 tri-snRNP complex localization | | orf_classification=Uncharacterized | | PRP4,IPF12538.2,Contig4-2379_0002,orf6.8424,CA5576,CaO19.7343,CAWG_02215 | |
| orf19.3724 | orf19.3724 |  | chrR | 538767 | 539576 | | Ortholog 8s 9 have role in endonucleolytic cleavage in 5 7-ETS of tricistronic rRNA transcript 8SSU-rRNA C 5.8S rRNA and LSU-rRNA 9 C more | | orf_classification=Uncharacterized | | orf19.11208,IPF7334.1,IPF28659.1,Contig4-3070_0036,orf6.1835,CA0730,CaO19.3724,IPF7334,CAWG_01584 | |
| orf19.2853 | orf19.2853 |  | chrR | 681673 | 682536 | | Ortholog of C. parapsilosis CDC317 A CPAR2_801070 C Candida tenuis NRRL Y-1498 A CANTEDRAFT_112987 C Debaryomyces hansenii CBS767 A DEHA2E09152g and Candida dubliniensis CD36 A CD36_28040 | | orf_classification=Uncharacterized | | orf19.10372,IPF24495.1,IPF11396.1,Contig4-2764_0006,orf6.3277,CA1668,CaO19.2853,CaO19.10372,IPF11396,CAWG_01632 | |
| orf19.4394 | orf19.4394 |  | chrR | 780330 | 782039 | | Predicted ORF in Assemblies 19 C 20 and 21 B transcription is induced in response to alpha pheromone in SpiderM medium | | orf_classification=Uncharacterized | | orf19.11872,IPF22876.1,IPF3301.2,Contig4-3077_0008,orf6.6313,CA3908,CaO19.11872,CaO19.4394,IPF3301,CAWG_01686 | |

**B.** Gene Ontology of stopped genes by Gene Ontology Term Finder tool on Candida Genome Database (CGD).

| GO_ID | GO_term | Cluster frequency | | | Adj pvalue | FDR | Gene(s) annotated to the term | Directly Annotated GOID List |  |
| --- | --- | --- | --- | --- | --- | --- | --- | --- | --- |
| 30447 | filamentous growth | | 11 out of 40 genes, 27.5% | 0.03956 | | 10.00% | VRG4:SNT1:PDX1:FAB1:FGR22:BUD20:PHR1:PPR1:CYR1:RFG1:ALS3 | 1900430:44117:44406:1900239:7033:44182:30448:1900439:19933:1900445:36180:43709:7155:36164:20012:1900443:33215:70887:44114:44409:70786:43708:7265:30447:1900429:31589:6355:15784:43710:1402:43065:1900231:6486:71216:9267:11:1900241:44011:122:7035:6171:71467:6897:70783:1900436:31505:36171:16337:36170:6357:45892:71244:9405:44407:35690:36166:30260 | |
| 50896 | response to stimulus | | 13 out of 40 genes, 32.5% | 0.04091 | | 5.00% | PSY2:SNT1:FAB1:FGR22:BUD20:PHR1:MIG1:PPR1:FET31:SSR1:CYR1:RFG1:TPO4 | 1900430:44117:44406:1900239:7033:6974:44182:1900439:19933:1900445:36180:43709:36164:20012:1900443:70887:44114:44409:70786:7265:1900429:30447:31589:6355:1402:43065:1900231:11:9267:71216:1900241:6470:44011:122:7035:6171:71467:15893:70783:1900436:31505:36171:15976:36170:6357:71244:45892:35690:44407:9405:36166 | |
| 40007 | growth | | 11 out of 40 genes, 27.5% | 0.04502 | | 3.33% | VRG4:SNT1:PDX1:FAB1:FGR22:BUD20:PHR1:PPR1:CYR1:RFG1:ALS3 | 1900430:44117:44406:1900239:7033:44182:30448:1900439:19933:1900445:36180:43709:7155:36164:20012:1900443:33215:70887:44114:44409:70786:43708:7265:1900429:30447:31589:6355:15784:43710:1402:43065:1900231:6486:11:9267:71216:1900241:44011:122:7035:6171:71467:6897:70783:1900436:31505:36171:16337:36170:6357:45892:71244:35690:44407:9405:36166:30260 | |
| 51716 | cellular response to stimulus | | 12 out of 40 genes, 30.0% | 0.04866 | | 2.50% | PSY2:SNT1:FAB1:FGR22:BUD20:PHR1:MIG1:PPR1:FET31:SSR1:CYR1:RFG1 | 1900430:44117:44406:1900239:7033:6974:44182:1900439:19933:1900445:36180:43709:36164:20012:1900443:70887:44114:44409:70786:7265:1900429:30447:31589:6355:1402:43065:1900231:11:9267:71216:1900241:6470:44011:122:7035:6171:71467:70783:1900436:31505:36171:15976:36170:6357:71244:45892:35690:44407:9405:36166 | |
| 71216 | cellular response to biotic stimulus | | 7 out of 40 genes, 17.5% | 0.08106 | | 5.60% | SNT1:FAB1:FGR22:BUD20:PPR1:CYR1:RFG1 | 1900430:1900239:7033:44182:1900439:19933:1900445:36180:43709:36164:20012:1900443:70887:70786:7265:1900429:30447:6355:1402:43065:1900231:11:9267:71216:1900241:44011:122:7035:6171:70783:1900436:36171:36170:6357:45892:71244:35690:9405:36166 | |

**Supplementary Table 5. Summary of stopped genes in the whole genome sequenced YQ2 isolate.** **A**. Information about stopped genes, including ORF number, systematic and standard name and description, as well as ORF position in the *C. albicans* SC5314 reference strain are reported. **B.** Gene Ontology of stopped genes by Gene Ontology Term Finder tool on Candida Genome Database (CGD). Significant at p < 0.05.

1. Information about stopped genes

| ORF | Parent ORF | Gene | ORF position (chr) in SC5314 reference strain | | | Description | Note | Alias |
| --- | --- | --- | --- | --- | --- | --- | --- | --- |
| orf19.2474 | orf19.2474 | *PRC3* | chr1 | 1206098 | 1207750 | Putative carboxypeptidase Y precursor B transcription is regulated by Nrg1p and Mig1p B regulated by Gcn2p and Gcn4p | orf_classification=Uncharacterized | CPY2,orf19.10011,IPF7205.2,IPF29082.1,Contig4-2954_0018,orf6.230,orf6.4961,CA0035,CaO19.2474,CAWG_00829 |
| orf19.6249 | orf19.6249 | *HAK1* | chr1 | 1413089 | 1415515 | Putative potassium transporter B similar to Schwanniomyces occidentalis Hak1p B amphotericin B induced B transcriptionally induced upon phagocytosis by macrophage B Hap43p-repressed gene | orf_classification=Uncharacterized | orf19.13627,IPF20886.1,IPF9136.3,Contig4-2748_0001,Contig4-2748_0010,orf6.1565,orf6.2201,CA0966,IPF9136,IPF9136.5eoc,IPF9136.2,CAWG_00751 |
| orf19.6193 | orf19.6193 | *TAF145* | chr1 | 1518264 | 1522049 | Protein similar to S. cerevisiae Taf145p C a component of RNA polymerase II transcription factor TFIID B flucytosine repressed B likely to be essential for growth C based on an insertional mutagenesis strategy | orf_classification=Uncharacterized | TAF1,orf19.8354,orf19.13573,IPF20901.1,IPF10000.2,IPF26567.1,IPF16558.2,orf6.996,orf6.5431,CA3221,CA0255,orf19.735,IPF10000.5eoc,CAWG_00705.1 |
| orf19.1233 | orf19.1233 | *ADE4* | chr1 | 1677134 | 1678759 | Putative phosphoribosylpyrophosphate amidotransferase B flucytosine induced | orf_classification=Uncharacterized | orf19.8818,IPF26073.1,IPF13283.2,Contig4-1515_0003,orf6.5084,CA2979,CaO19.1233,CaO19.8818,CAWG_00646 |
| orf19.4757 | orf19.4757 | *NAR1* | chr1 | 1962576 | 1964405 | Putative cytosolic iron-sulfur 8FeS 9 protein assembly machinery protein B induced by nitric oxide B oxidative stress-induced via Cap1p | orf_classification=Uncharacterized | orf19.12221,IPF22395.1,IPF14388.2,Contig4-2557_0009,Contig4-2557_0010,orf6.4598,CA2632,CaO19.12221,CaO19.4757,CAWG_00528 |
| orf19.5094 | orf19.5094 | *BUL1* | chr1 | 1780738 | 1782963 | Protein not essential for viability B macrophage Fpseudohyphal-induced B similar to S. cerevisiae Bul1p C which may be involved in selection of substrates for ubiquitination | orf_classification=Uncharacterized | BUL5,orf19.12560,IPF22119.1,IPF12963.2,Contig4-2641_0004,Contig4-2641_0005,orf6.6282,CA3886,CaO19.12560,CaO19.5094,IPF12963,CAWG_00606 |
| orf19.4822 | orf19.4822 | *LIP10* | chr1 | 2111781 | 2113178 | Secreted lipase C member of a lipase gene family whose members are expressed differentially in response to carbon source and during infection B may have a role in nutrition and For in creating an acidic microenvironment |  | orf19.12285,IPF22423.1,IPF11790.1,Contig4-2746_0010,orf6.7389,CA4757,CaO19.4822,CaO19.12285,CAWG_00471 |
| orf19.4890 | orf19.4890 | *CLA4* | chr1 | 2249543 | 2252473 | Ste20p family Ser FThr kinase required for wild-type filamentous growth C organ colonization and virulence in mouse systemic infection B role in chlamydospore formation B functional homolog of S. cerevisiae Cla4p B mutant caspofungin sensitive |  | orf19.12355,IPF15972.3,IPF16297.2,Contig4-2934_0017,orf6.3334,orf6.53,CA1710,CaO19.4890,CAWG_00412 |
| orf19.3756 | orf19.3756 | *CHR1* | chr1 | 2747753 | 2749675 | Predicted DEAD-box ATP-dependent RNA helicase C functional homolog of S. cerevisiae Rok1p B Hap43p-induced gene | orf_classification=Uncharacterized | orf19.11240,IPF23503.1,IPF8903.2,Contig4-2804_0019,orf6.2262,CA1002,CaO19.3756,CaO19.11240,ROK1.3f,ROK1.3,CAWG_00182 |
| orf19.3693 | orf19.3693 | *PGA5* | chr1 | 490289 | 492214 | Putative GPI-anchored beta-1 C3-glucanosyltransferase with similarity to the A. fumigatus GEL family B fungal-specific 8no human or murine homolog 9 | orf_classification=Uncharacterized | GAS12,orf19.11177,IPF23587.1,IPF10714.2,Contig4-2582_0001,orf6.3873,CA2106,CaO19.11177,CaO19.3693,IPF10714,CAWG_01148 |
| orf19.2941 | orf19.2941 | *SCW4* | chr1 | 530226 | 531962 | Putative cell wall protein B exogenously expressed protein is a substrate for Kex2p processing in vitro B expression is regulated upon white-opaque switching B alkaline downregulated B possibly essential gene 8UAU1 method 9 B biofilm-induced | orf_classification=Uncharacterized | CMP66,orf19.10458,IPF24282.1,IPF8122.1,Contig4-2841_0015,orf6.4005,CA2202,CaO19.10458,CaO19.2941,CAWG_01129 |
| orf19.1223 | orf19.1223 | *DBF2* | chr2 | 1357902 | 1360034 | Essential serine Fthreonine protein kinase involved in mitotic spindle formation and cytokinesis B required for septum formation C exit from mitosis C and normal hyphal morphogenesis B virulence-group-correlated expression | orf_classification=Verified | orf19.8809,IPF26112.1,IPF13853.2,Contig4-1569_0001,orf6.851,CA0205,CaO19.8809,CaO19.1223,CAWG_04401 |
| orf19.2238 | orf19.2238 | *LTE1* | chr2 | 1407349 | 1412304 | Protein similar to S. cerevisiae Lte1p B transcription is repressed in response to alpha pheromone in SpiderM medium | orf_classification=Uncharacterized | LTE99,orf19.9780,orf19.9781,IPF25036.1,IPF13694.2,IPF25034.1,IPF19721.1,Contig4-2781_0024,Contig4-2838_0001,Contig4-2838_0002,orf6.2394,orf6.2393,CA1077,CA1076,orf19.2239,CaO19.9780,CaO19.2238,IPF13694,CAWG_05785.1 |
| orf19.2241 | orf19.2241 | *PST1* | chr2 | 1412670 | 1413266 | Putative 1 C4-benzoquinone reductase B biofilm induced B hyphal-induced expression C regulated by Cyr1p C Ras1p C Efg1p C Nrg1p C Rfg1p C Tup1p B Hap43p-induced gene | orf_classification=Uncharacterized | IPF17954.2,IPF18769.1,Contig4-2561_0001,orf6.1034,orf6.2391,CA6202,IPF18769,CAWG_05786 |
| orf19.4084 | orf19.4084 | *KIS1* | chr2 | 1891108 | 1892346 | Snf1p complex scaffold protein B similar to S. cerevisiae Gal83p and Sip2p with regions of similarity to Sip1p 8ASC and KIS domain 9 B interacts with Snf4p B mutants are hypersensitive to caspofungin and hydrogen peroxide B Hap43p-repressed gene |  | GAL83,orf19.11565,IPF2078.2,IPF2079.2,Contig4-3095_0014,orf6.6852,CA4335,CaO19.4084,CaO19.11565,CAWG_06000 |
| orf19.5967 | orf19.5967 | *FGR44* | chr3 | 1073190 | 1074422 | Protein lacking an ortholog in S. cerevisiae B transposon mutation affects filamentous growth |  | orf19.13388,IPF21226.1,IPF44.1,Contig4-3042_0010,orf6.9070,CA6090,CaO19.5967,CaO19.13388,IPF44,CAWG_02811 |
| orf19.5389 | orf19.5389 | *FKH2* | chr3 | 113160 | 115299 | Forkhead transcription factor B morphogenesis regulator B required for wild-type hyphal transcription C cell separation C and for virulence in cell culture B mutant lacks true hyphae C is constitutively pseudohyphal B upregulated in RHE model | orf_classification=Verified | FKH1,orf19.12844,orf6.8625,IPF21713.1,IPF1059.2,IPF1060.2,Contig4-3071_0032,orf6.8626,CA5753,CaO19.12844,CaO19.5389,CA5754,IPF1060,CAWG_02404 |
| orf19.7394 | orf19.7394 | *GDA1* | chr3 | 1393822 | 1395621 | Golgi membrane GDPase C required for wild-type O-mannosylation C not N-glycosylation B required for wild-type hyphal induction C cell wall C and cell surface charge B not required for HeLa cell adherence B functional homolog of S. cerevisiae Gda1p |  | IPF3313.1,Contig4-3036_0003,orf6.8554,CA5693,CaO19.7394,CAWG_02921 |
| orf19.6760 | orf19.6760 | *MDS3* | chr3 | 1683073 | 1687224 | TOR signaling pathway component required for growth and hyphal formation at pH 9 C for full virulence in a mouse model of systemic infection and for biofilm formation B plays a role in chlamydospore formation |  | orf19.14051,orf19.14052,IPF20325.1,IPF3482.2,IPF20324.1,IPF2784.2,Contig4-2141_0007,Contig4-3030_0020,orf6.8872,orf6.8871,CA5938,CA5937,CaO19.14052,CaO19.6760,CaO19.6759,CaO19.14051,IPF2784,IPF3481,orf19.6759,CAWG_03040 |
| orf19.1704 | orf19.1704 | *FOX3* | chr3 | 311546 | 312778 | Putative peroxisomal 3-oxoacyl CoA thiolase B expression is regulated upon white-opaque switching | orf_classification=Uncharacterized | POT2,POT12,POT1,orf19.9271,IPF17552.3,IPF16087.1,POT1-1,Contig4-2869_0003,orf6.3123,CA1561,CaO19.9271,CaO19.1704,CA1505,IPF17552.2,CAWG_02477 |
| orf19.262 | orf19.262 | *SMC3* | chr3 | 583230 | 586943 | Protein similar to S. cerevisiae Smc3p C which is an ATPase involved in sister chromatid cohesion B likely to be essential for growth C based on an insertional mutagenesis strategy | orf_classification=Uncharacterized | orf19.7895,IPF12682.2,IPF18780.2,IPF18779.1,Contig4-2723_0011,orf6.2013,orf6.2357,orf6.2358,CA0846,CA6315,orf19.263,SMC3.5f,CAWG_02593 |
| orf19.3796 | orf19.3796 | *DCR1* | chr4 | 1037525 | 1039360 | Putative Dicer RNAse involved in RNA interference C similar to S. cerevisiae Rnt1p but orthologous to S. castellii Dcr1p C which is not conserved in S. cerevisiae |  | RNT1,orf19.11277,IPF14250.2,IPF6904.1,Contig4-2569_0003,Contig4-2569_0004,orf6.2914,orf6.3069,CA1420,CaO19.3796,CAWG_03333 |
| orf19.1291 | orf19.1291 | *ABZ1* | chr4 | 1151753 | 1154221 | Ortholog 8s 9 have 4-amino-4-deoxychorismate synthase activity C role in para-aminobenzoic acid biosynthetic process and cytosol C nucleus localization | orf_classification=Uncharacterized | orf19.9352,orf19.9353,IPF10474.2,Contig4-2660_0010,Contig4-2660_0012,orf6.1155,orf6.910,orf6.1154,CA0220,orf19.1786,orf19.1787,CaO19.1291,CAWG_03285 |
| orf19.4719 | orf19.4719 | *CWH41* | chr4 | 1355471 | 1357963 | Processing alpha glucosidase I C involved in N-linked protein glycosylation and assembly of cell wall beta 1 C6 glucan |  | GLS1,PRB1,orf19.11899,IPF22852.1,IPF15907.2,IPF8229.2,Contig4-2812_0012,orf6.4218,orf6.3155,CA2350,CA1581,orf19.4421,CaO19.4719,CWH41.3eoc,CAWG_03215 |
| orf19.2884 | orf19.2884 | *CDC68* | chr4 | 1444317 | 1447499 | Functional homolog of S. cerevisiae Cdc68p C which is a transcription elongation factor B essential B possible drug target |  | SPT16,orf19.10402,IPF24469.1,IPF10512.3,Contig4-2689_0011,orf6.2896,CA1409,CaO19.2884,CaO19.10402,IPF10512.2,CAWG_03177 |
| orf19.4683 | orf19.4683 | *MLP1* | chr4 | 209114 | 214612 | Ortholog 8s 9 have ribonucleoprotein complex binding activity | orf_classification=Uncharacterized | orf19.12152,IPF22637.1,IPF10559.2,Contig4-1629_0001,orf6.5453,orf6.68,CA3240,CaO19.4683,IPF10559,CAWG_03675 |
| orf19.2758 | orf19.2758 | *PGA38* | chr4 | 493613 | 495160 | Putative GPI-anchored protein B adhesin-like protein B repressed during cell wall regeneration B possibly an essential gene C disruptants not obtained by UAU1 method | orf_classification=Uncharacterized | orf19.10273,orf19.10272,IPF11998.3,IPF17815.2,orf6.1331,orf6.3591,orf6.1332,orf6.3590,CA1898,orf19.2759,IPF11998,IPF11998.2,CAWG_03551 |
| orf19.2748 | orf19.2748 | *ARG83* | chr4 | 517410 | 520334 | Zinc-finger protein B clade-associated gene expression B null mutant shows abnormal regulation of invasive colony growth and is unable to utilize proline as a nitrogen source B late-stage biofilm-induced |  | orf19.10262,IPF24568.1,IPF13264.2,Contig4-2217_0009,Contig4-2945_0026,orf6.2536,CA1171,CaO19.10262,CaO19.2748,IPF13264,CAWG_03542 |
| orf19.1311 | orf19.1311 | *SPO75* | chr4 | 782925 | 785510 | Ortholog 8s 9 have role in ascospore wall assembly | orf_classification=Uncharacterized | orf19.8891,IPF6671.2,IPF29492.1,Contig4-2805_0006,orf6.3102,CA1546,CaO19.8891,CaO19.1311,IPF6671,CAWG_03446 |
| orf19.789 | orf19.789 | *PYC2* | chr4 | 837806 | 841339 | Putative pyruvate carboxylase C binds to biotin cofactor B up-regulated in mutant lacking the Ssk1p response regulator protein C upon benomyl treatment C or in an azole-resistant strain overexpressing MDR1 B stationary phase enriched protein | orf_classification=Uncharacterized | orf19.8408,orf6.2989,IPF26382.1,IPF26504.1,IPF15163.2,IPF15167.2,Contig4-3027_0007,CA1464,PYC2.3f,PYC2.exon2,CAWG_03418 |
| orf19.4045 | orf19.4045 | *EST1* | chr5 | 1176415 | 1178253 | Telomerase subunit B allosteric activator of catalytic activity C but not required for catalytic activity B has TPR domain | orf_classification=Verified | orf19.11527,orf6.4411,IPF23224.1,IPF7224.2,Contig4-2715_0009,CA2492,CaO19.11527,CaO19.4045,IPF7224,CAWG_04912 |
| orf19.4145 | orf19.4145 | *ZCF20* | chr5 | 333155 | 336412 | Predicted zinc-finger protein of unknown function B regulated by Sef1p C Sfu1p C and Hap43p B Hap43p-induced | orf_classification=Uncharacterized | orf19.11621,IPF9826.2,IPF19172.2,Contig4-2294_0005,Contig4-2911_0001,orf6.236,orf6.5234,orf6.720,CA3088,CaO19.4145,IPF9826,CAWG_04537 |
| orf19.3190 | orf19.3190 | *HAL9* | chr5 | 411974 | 415006 | Protein with Zn 82 9-Cys 86 9 binuclear cluster B gene in zinc cluster region of Chr. 5 B transcriptionally activated by Mnl1p in weak acid B similar to S. cerevisiae Hal9p C which is a putative transcription factor involved in salt tolerance |  | ZNC3,HAL93,orf19.10701,IPF9196.1,IPF16067.2,Contig4-1956_0001,Contig4-1956_0002,orf6.4487,CA2542,CaO19.3190,IPF16067,CAWG_04568 |
| orf19.939 | orf19.939 | *NAM7* | chr5 | 95043 | 98102 | Putative role in nonsense-mediated mRNA decay B similar to S. cerevisiae Nam7p B gene induced by ciclopirox olamine treatment | orf_classification=Uncharacterized | orf19.8554,IPF26374.1,IPF9662.2,Contig4-2612_0009,orf6.8188,CA5396,CaO19.939,CaO19.8554,CAWG_04444 |
| orf19.2158 | orf19.2158 | *NAG3* | chr6 | 1023473 | 1025158 | Putative transporter of the major facilitator superfamily 8MFS 9 B similar to Nag4p B required for wild-type mouse virulence and cycloheximide resistance B in gene cluster that includes genes encoding enzymes of GlcNAc catabolism |  | MDR97,TMP1,orf6.683,orf6.682,IPF2706.2,IPF2714.2,Contig4-2515_0003,orf6.2472,CA6268,CA1132,orf19.2159,IFY2.3f,CAWG_04920 |
| orf19.5557 | orf19.5557 | *MNN4-4* | chr6 | 583682 | 586300 | Mannosyltransferase B transcription is upregulated in a mutant lacking the Ssk1p response regulator proteinor in a nik1 homozygous null mutant C but not in chk1 or sln1 null mutants B pheromone induced B planktonic growth-induced |  | MNT4,MNN4,MNN45,orf19.13003,orf6.4127,IPF21572.1,IPF5376.2,Contig4-3002_0015,CA2288,CaO19.13003,CaO19.5557,IPF5376,CAWG_05079 |
| orf19.6510 | orf19.6510 | *GRX1* | chr7 | 461000 | 461371 | Putative glutaredoxin B transcriptionally regulated by iron B expression greater in low iron | orf_classification=Uncharacterized | orf19.13863,orf6.7650,IPF20674.1,IPF3919.2,Contig4-3089_0003,CA4963,CaJ7.0239,CaO19.6510,CaJ7_0239,CaO19_6510,CaO19.13863,IPF3919,CAWG_05557 |
| orf19.5172 | orf19.5172 | *LIP9* | chr7 | 622142 | 623503 | Secreted lipase C member of a lipase gene family whose members are expressed differentially in response to carbon source and during infection B may have a role in nutrition and For in creating an acidic microenvironment | orf_classification=Uncharacterized | orf19.12639,orf19.12640,IPF22071.1,IPF22065.1,IPF5407.2,IPF5406.2,Contig4-3092_0045,orf6.6964,orf6.6963,CA4423,CA4422,CaJ7.0329,orf19.5173,LIP9.5f,LIP9.exon1,CAWG_05624 |
| orf19.1805 | orf19.1805 | *PEX14* | chrR | 1066201 | 1067559 | Ortholog 8s 9 have protein binding C bridging activity C role in protein import into peroxisome matrix C docking and peroxisomal membrane localization | orf_classification=Uncharacterized | orf19.9371,IPF15186.2,IPF14862.2,Contig4-2476_0006,orf6.1160,orf6.1578,CA0573,CAWG_01827 |
| orf19.695 | orf19.695 | *RGS2* | chrR | 1409092 | 1410390 | Protein of RGS superfamily B not essential for viability | orf_classification=Uncharacterized | orf19.8314,IPF18512,IPF26603.1,IPF18512.1,orf6.4013,CA2209,CaO19.8314,CaO19.695 |
| orf19.7472 | orf19.7472 | *IFF4* | chrR | 145031 | 149611 | Adhesin-like cell surface protein B putative GPI-anchor B null mutant germ tubes show decreased adhesion to plastic substrate B not essential for viability B Hap43p-repressed gene |  | IPF2507.1,Contig4-3027_0015,orf6.8724,CA5819,CaO19.7472,CAWG_01420 |
| orf19.6420 | orf19.6420 | *PGA13* | chrR | 1838917 | 1840287 | GPI-anchored cell wall protein involved in cell wall synthesis B required for normal cell surface properties B induced in oralpharyngeal candidasis |  | orf19.13778,IPF20734.1,IPF1341.1,Contig4-3073_0004,orf6.7834,CA5112,CaO19.6420,CaO19.13778,IPF1341,CAWG_02138 |
| orf19.3221 | orf19.3221 | *CPA2* | chrR | 318330 | 321779 | Putative arginine-specific carbamoylphosphate synthetase B protein enriched in stationary phase yeast cultures B transcription is upregulated in both intermediate and mature biofilms | orf_classification=Uncharacterized | orf19.10732,IPF24081.1,IPF4967.1,Contig4-2886_0008,CA0687,CaO19.10732,CaO19.3221,orf6.1765,CAWG_01485 |
| orf19.2616 | orf19.2616 | *UGT51C1* | chrR | 470546 | 475087 | UDP-glucose Asterol glucosyltransferase B enzyme of sterol glucoside 8membrane-bound lipid 9 biosynthesis B has UDP-sugar binding domain B activity is UDP-glucose-specific in vitro B enzyme does not use UDP-mannose B Mig1p-regulated gene |  | UGT51,orf19.10147,IPF24795.1,IPF7800.3,Contig4-2761_0001,orf6.1655,orf6.4269,CA0618,CaO19.2616,IPF7800.2,CAWG_01562 |
| orf19.3753 | orf19.3753 | *SEF1* | chrR | 479692 | 482445 | Zn2-Cys6 transcription factor B regulates iron uptake B transcription is negatively regulated by Sfu1p and positively regulated by Tbf1p B promotes virulence in mouse systemic infection B mutants display decreased colonization of mouse kidneys |  | orf19.11237,IPF23521.1,IPF7790.2,Contig4-2761_0009,Contig4-2761_0013,orf6.4213,CA2346,CaO19.11237,CaO19.3753,CAWG_01565 |
| orf19.4379 | orf19.4379 | *PRP13* | chrR | 849450 | 852053 | Putative integral inner mitochondrial membrane protein with similarity to exonucleases | orf_classification=Uncharacterized | PRP12,PRP122,orf19.11829,orf19.11857,IPF15868.3,IPF12922.2,IPF22865.1,IPF28423.1,Contig4-2577_0006,Contig4-2720_0005,Contig4-2720_0006,orf6.2807,orf6.4544,orf6.6327,orf6.352,orf6.575,CA1349,orf19.4351,CaO19.4379,CAWG_01716 |
| orf19.760 | orf19.760 |  | chr1 | 1003980 | 1004351 | Ortholog 8s 9 have role in ribosome biogenesis and mitochondrion localization | orf_classification=Uncharacterized | GON5,orf19.8380,IPF26539.1,IPF3903.2,Contig4-2890_0010,orf6.3639,CA1932,CaO19.8380,CaO19.760,IPF3903,CAWG_00913 |
| orf19.51 | orf19.51 |  | chr1 | 1036865 | 1040956 | Ortholog 8s 9 have role in mitochondrion organization C translational initiation and eukaryotic translation initiation factor 3 complex localization | orf_classification=Uncharacterized | CLU1,orf19.7712,IPF27203.1,IPF11177.2,Contig4-2627_0005,orf6.4555,CA2600,CaO19.7712,CaO19.51,IPF11177,CAWG_00900 |
| orf19.2440 | orf19.2440 |  | chr1 | 1268667 | 1271219 | Predicted ORF in Assemblies 19 C 20 and 21 B transcription is induced in response to alpha pheromone in SpiderM medium | orf_classification=Uncharacterized | orf19.9976,orf19.9977,IPF24850.1,IPF24836.1,IPF7479.2,IPF7478.2,orf6.3618,orf6.3619,CA1917,CA1918,orf19.2441,IPF7479.3f,CAWG_00803 |
| orf19.6280 | orf19.6280 |  | chr1 | 1340369 | 1340722 | Dubious open reading fram | orf_classification=Dubious | Alias=orf19.13659,IPF20954.1,IPF27787.1,orf6.6039,CaO19.13659,CaO19.6280 |
| orf19.6277 | orf19.6277 |  | chr1 | 1344908 | 1348267 | Has domain 8s 9 with predicted phosphatidylinositol binding activity and role in cell communication | orf_classification=Uncharacterized | orf19.13656,orf19.13658,IPF19586.1,IPF19587.2,orf6.501,orf6.6038,CA3701,CA3702,orf19.6279,IPF11936.3f,orf6.6036,CAWG_00778.1 |
| orf19.6234 | orf19.6234 |  | chr1 | 1442891 | 1444495 | Putative U2 snRNP component B mutation confers hypersensitivity to 5-fluorocytosine 85-FC 9 C 5-fluorouracil 85-FU 9 C and tubercidin 87-deazaadenosine 9 B Hap43p-induced C biofilm-induced |  | orf19.13614,IPF12148.1,IPF12516.1,Contig4-2906_0007,orf6.1584,orf6.5122,CA3007,CaO19.6234,IPF12148,CAWG_00737 |
| orf19.1229 | orf19.1229 |  | chr1 | 1669828 | 1672965 | Ortholog 8s 9 have importin-alpha export receptor activity C role in cell division C protein export from nucleus and nuclear membrane localization | orf_classification=Uncharacterized | orf19.8815,orf19.8816,IPF16520.2,IPF26077.1,IPF19119.2,IPF29521.1,IPF13285.2,Contig4-1270_0001,Contig4-1270_0002,Contig4-2208_0002,orf6.5087,orf6.869,orf6.5086,CA6253,CA2982,CA2981,orf19.1230,orf19.1231,CSE1.3f,CAWG_00648 |
| orf19.5092 | orf19.5092 |  | chr1 | 1778627 | 1779265 | Ortholog of Candida tenuis NRRL Y-1498 A CANTEDRAFT_113324 C Debaryomyces hansenii CBS767 A DEHA2C11088g C Candida dubliniensis CD36 A CD36_07680 and Candida albicans WO-1 A CAWG_00608 | orf_classification=Uncharacterized | orf19.12558,IPF22155.1,IPF12967.1,Contig4-2641_0001,orf6.6284,CA3888,CaO19.12558,CaO19.5092,IPF12967,CAWG_00608 |
| orf19.398 | orf19.398 |  | chr1 | 1867938 | 1868411 | Ortholog of C. parapsilosis CDC317 A CPAR2_207180 C Candida tenuis NRRL Y-1498 A CANTEDRAFT_114140 C Debaryomyces hansenii CBS767 A DEHA2G13992g and Candida dubliniensis CD36 A CD36_08040 | orf_classification=Uncharacterized | orf19.8028,IPF26929.1,IPF8828.2,Contig4-3031_0007,orf6.6266,CA3871,CaO19.8028,CaO19.398,IPF8828,CAWG_00573 |
| orf19.4893 | orf19.4893 |  | chr1 | 2259391 | 2263716 | Protein not essential for viability | orf_classification=Uncharacterized | orf19.12358,IPF12282,IPF22500.1,IPF12282.2,Contig4-2955_0011,Contig4-2955_0014,orf6.4229,CA2356,CaO19.12358,CaO19.4893,CAWG_00410 |
| orf19.994 | orf19.994 |  | chr1 | 2325641 | 2326189 | Predicted ORF in Assemblies 19 C 20 and 21 B transcriptionally activated by Mnl1p under weak acid stress | orf_classification=Uncharacterized | orf19.8609,IPF26297.1,IPF20134.1,Contig4-2932_0016,orf6.5804,CA3522,CaO19.8609,CaO19.994,IPF20134,CAWG_00382 |
| orf19.2350 | orf19.2350 |  | chr1 | 2376214 | 2377776 | Protein similar to S. cerevisiae Yor378wp B transposon mutation affects filamentous growth B not essential for viability B member of MFS family of transporters B fungal-specific 8no human or murine homolog 9 |  | orf19.9886,IPF13377,IPF24976.1,IPF13377.2,Contig4-2380_0006,Contig4-2380_0007,orf6.4202,YOR378W,CA2339,CaO19.9886,CaO19.2350,CAWG_00362.1 |
| orf19.6347 | orf19.6347 |  | chr1 | 2779648 | 2781210 | Ortholog 8s 9 have second spliceosomal transesterification activity | orf_classification=Uncharacterized | CDC40,orf19.13703,IPF20799.1,IPF14486.2,Contig4-2232_0002,orf6.5776,CA3501,CaO19.6347,CaO19.13703,CAWG_00167 |
| orf19.3689 | orf19.3689 |  | chr1 | 484881 | 486512 | Putative protein similar to 6-phosphofructo-2-kinase Ffructose-2 C6-bisphosphatase B expression downregulated in an ssr1 null mutant | orf_classification=Uncharacterized | orf19.11173,orf19.11174,IPF23610.1,IPF23605.1,IPF18544.1,IPF13749.2,Contig4-2096_0004,Contig4-2096_0006,Contig4-2582_0008,orf6.3877,orf6.3876,CA2110,CA2109,orf19.3690,IPF13749.3f,CAWG_01151 |
| orf19.3021 | orf19.3021 |  | chr1 | 683972 | 685176 | Putative protein of unknown function B Hap43p-repressed gene B induced during planktonic growth | orf_classification=Uncharacterized | orf19.10539,IPF24316.1,IPF3701.1,Contig4-3096_0044,orf6.7758,CA5055,IPF3701,CAWG_01057 |
| orf19.1057 | orf19.1057 |  | chr1 | 878815 | 879384 | Ortholog of C. parapsilosis CDC317 A CPAR2_107050 C Candida tenuis NRRL Y-1498 A CANTEDRAFT_113999 C Debaryomyces hansenii CBS767 A DEHA2D12518g and Candida dubliniensis CD36 A CD36_03980 | orf_classification=Uncharacterized | orf19.8659,IPF26268.1,IPF17234.2,Contig4-2089_0004,Contig4-2089_0005,orf6.4932,CA2864,CaO19.1057,CaO19.8659,IPF17234.3f,IPF17234.3,CAWG_00971 |
| orf19.4127 | orf19.4127 |  | chr2 | 1013161 | 1014567 | Ortholog 8s 9 have actin filament binding activity C role in actin cortical patch localization C actin filament bundle assembly C endocytosis and actin cortical patch localization | orf_classification=Uncharacterized | IPF13275.2,Contig4-2218_0002,orf6.2516,CA1158,IPF13275,CAWG_04241 |
| orf19.4125 | orf19.4125 |  | chr2 | 1015910 | 1017148 | Putative transcription factor with zinc finger DNA-binding motif B Hap43p-induced | orf_classification=Uncharacterized | PZF1,IPF13278.2,Contig4-2828_0002,orf6.2514,CA1157,CaO19.4125,CAWG_04242 |
| orf19.5491 | orf19.5491 |  | chr2 | 1277233 | 1278420 | Ortholog of C. parapsilosis CDC317 A CPAR2_104720 C Candida tenuis NRRL Y-1498 A CANTEDRAFT_103989 C Debaryomyces hansenii CBS767 A DEHA2F02046g and Candida dubliniensis CD36 A CD36_20670 | orf_classification=Uncharacterized | orf19.12946,IPF5834.2,IPF28015.1,Contig4-2947_0022,orf6.4185,orf6.4666,CA2678,CaO19.5491,IPF5834,CAWG_04372 |
| orf19.4090 | orf19.4090 |  | chr2 | 1896799 | 1898304 | Predicted membrane transporter C member of the fucose Aproton symporter 8FHS 9 family C major facilitator superfamily 8MFS 9 | orf_classification=Uncharacterized | orf19.11571,IPF23141.1,IPF2087.2,Contig4-3095_0024,orf6.6846,CA4330,CaO19.11571,CaO19.4090,IPF2087,CAWG_06005 |
| orf19.851 | orf19.851 |  | chr2 | 776966 | 779482 | Protein of unknown function B transcription is negatively regulated by Rim101p B negatively modulates intracellular ATP levels during the development of azole resistance B induced by Ca 82 B 9 in a calcineurin-dependent manner | orf_classification=Uncharacterized | MNN42,orf19.8471,orf6.1543,IPF26385.1,IPF14030.2,Contig4-2545_0003,Contig4-2545_0006,CA0550,CaO19.8471,CaO19.851,IPF14030,CAWG_04132 |
| orf19.149 | orf19.149 |  | chr2 | 980006 | 980443 | Predicted ORF in Assemblies 19 C 20 and 21 B transcription detected in high-resolution tiling array experiments | orf_classification=Uncharacterized | IPF27154.1,IPF29941.1,orf6.62,CAWG_04221 |
| orf19.5390 | orf19.5390 |  | chr3 | 113160 | 113603 | Ortholog of Candida albicans WO-1 A CAWG_02403 | orf_classification=Uncharacterized | FKH1,FKH2,orf19.12845,orf6.8625,IPF1059.2,IPF21770.1,IPF1060.2,Contig4-3071_0030,orf6.8626,CA5753,CaO19.12845,CaO19.5390,CA5754,IPF1060,CAWG_02403 |
| orf19.6966 | orf19.6966 |  | chr3 | 1168409 | 1170277 | Ortholog 8s 9 have choline kinase activity C ethanolamine kinase activity C role in phosphatidylcholine biosynthetic process C phosphatidylethanolamine biosynthetic process and cytosol C nucleus localization | orf_classification=Uncharacterized | CKI1,IPF11120.1,Contig4-2580_0006,orf6.7712,CA5014,CaO19.6966,IPF11120,CAWG_02843 |
| orf19.7368 | orf19.7368 |  | chr3 | 1318949 | 1320481 | Ortholog 8s 9 have mRNA binding C poly 8U 9 RNA binding activity and role in nuclear-transcribed mRNA catabolic process C nonsense-mediated decay C regulation of mRNA stability C stress granule assembly | orf_classification=Uncharacterized | PUB1,IPF1257.1,Contig4-3075_0021,orf6.8528,CA5666,CaO19.7368,CAWG_02897 |
| orf19.6715 | orf19.6715 |  | chr3 | 1757968 | 1758276 | Ortholog of Candida dubliniensis CD36 A CD36_87595 and Candida albicans WO-1 A CAWG_03078 | orf_classification=Dubious | orf19.14007,IPF20459.1,IPF27667.1,orf6.6864,CaO19.14007,CaO19.6715,CAWG_03078 |
| orf19.2513 | orf19.2513 |  | chr3 | 223648 | 225798 | Ortholog of C. parapsilosis CDC317 A CPAR2_103060 C Candida tenuis NRRL Y-1498 A CANTEDRAFT_127339 C Debaryomyces hansenii CBS767 A DEHA2G01936g and Candida dubliniensis CD36 A CD36_81010 | orf_classification=Uncharacterized | orf19.10049,IPF24827.1,IPF12802.2,Contig4-1978_0004,Contig4-1978_0006,orf6.3918,CA2144,CaO19.2513,CaO19.10049,IPF12802,CAWG_02445 |
| orf19.2515 | orf19.2515 |  | chr3 | 227430 | 230084 | Has domain 8s 9 with predicted zinc ion binding activity | orf_classification=Uncharacterized | orf19.10051,IPF24819.1,IPF12799.2,orf6.3920,CA2146,CaO19.10051,CaO19.2515,IPF12799,CAWG_02447 |
| orf19.261 | orf19.261 |  | chr3 | 580882 | 582642 | Ortholog 8s 9 have dolichol kinase activity C role in protein glycosylation and endoplasmic reticulum membrane localization | orf_classification=Uncharacterized | SEC59,orf19.7894,IPF12686.3,IPF13722.2,Contig4-2723_0010,orf6.1456,orf6.2014,orf6.549,CA0488,CaO19.261,CAWG_02592 |
| orf19.746 | orf19.746 |  | chr4 | 1117501 | 1119048 | Has domain 8s 9 with predicted role in protein transport | orf_classification=Uncharacterized | orf19.8365,IPF26553.1,IPF9748.2,Contig4-2360_0002,Contig4-2360_0003,orf6.4135,CA2293,CaO19.746,CaO19.8365,IPF9748,CAWG_03299 |
| orf19.2899 | orf19.2899 |  | chr4 | 1412506 | 1413294 | Ortholog 8s 9 have cytosol C nucleus localization | orf_classification=Uncharacterized | OAR2,orf19.10417,IPF24439.1,IPF13467.1,Contig4-2946_0003,orf6.6123,CA3768,CaO19.2899,CaO19.10417,IPF13467,CAWG_03191 |
| orf19.4686 | orf19.4686 |  | chr4 | 202436 | 206038 | Ortholog of S. cerevisiae A YIL151C C C. glabrata CBS138 A CAGL0H06611g C C. parapsilosis CDC317 A CPAR2_401340 and Candida tenuis NRRL Y-1498 A CANTEDRAFT_104645 | orf_classification=Uncharacterized | orf19.12155,IPF18902.2,IPF10555.3,Contig4-2733_0012,orf6.1766,orf6.5450,CA3238,CaO19.4686,IPF10555,CA0688,IPF10555.3eoc,IPF18902.1,CAWG_03678 |
| orf19.2761 | orf19.2761 |  | chr4 | 487652 | 488449 | Putative glycosylphosphatidylinositol 8GPI 9 anchor assembly protein B transposon insertion causes decreased colony wrinkling but does not block true hyphal growth B induced by nitric oxide independent of Yhb1p |  | GPI11,orf19.10277,IPF24625.1,IPF11995.2,Contig4-2938_0025,orf6.1334,orf6.6591,CA0427,CaO19.2761,IPF11995,CAWG_03553 |
| orf19.2686 | orf19.2686 |  | chr4 | 633978 | 635720 | Ortholog 8s 9 have carboxypeptidase activity C role in nitrogen compound metabolic process C proteolysis involved in cellular protein catabolic process and fungal-type vacuole lumen localization | orf_classification=Uncharacterized | CPS1,orf19.10201,IPF19440.2,IPF7980.2,Contig4-2865_0001,orf6.116,orf6.4796,CA2770,CaO19.2686,CAWG_03494 |
| orf19.1326 | orf19.1326 |  | chr4 | 750848 | 752818 | Ortholog of Candida albicans WO-1 A CAWG_03455 | orf_classification=Uncharacterized | LPF13,IFA7,orf19.8906,IPF26011.1,IPF14593.2,Contig4-2287_0002,orf6.4888,CA2829,CaO19.8906,CaO19.1326,CAWG_03455 |
| orf19.5043 | orf19.5043 |  | chr4 | 822239 | 823705 | Ortholog of C. parapsilosis CDC317 A CPAR2_403770 C Debaryomyces hansenii CBS767 A DEHA2G16962g C Candida dubliniensis CD36 A CD36_43610 and Pichia stipitis Pignal A PICST_30926 | orf_classification=Uncharacterized | orf19.12510,IPF17702.3,IPF16758.1,Contig4-1448_0002,Contig4-2272_0007,Contig4-2272_0008,orf6.3320,orf6.429,CA1697,CaO19.5043,IPF16758,CAWG_03424 |
| orf19.787 | orf19.787 |  | chr4 | 844616 | 844921 | Ortholog of Candida albicans WO-1 A CAWG_03417 | orf_classification=Uncharacterized | orf19.8406,IPF26483.1,IPF29675.1,orf6.558,CaO19.787,CaO19.8406,CAWG_03417 |
| orf19.5304 | orf19.5304 |  | chr4 | 869238 | 869603 | Ortholog of Candida dubliniensis CD36 A CD36_43800 and Candida albicans WO-1 A CAWG_03395 | orf_classification=Dubious | orf19.12764,IPF21881.1,IPF8528.1,Contig4-2961_0010,orf6.8293,CaO19.5304,CaO19.12764,CAWG_03395 |
| orf19.5300 | orf19.5300 |  | chr4 | 876819 | 878564 | Predicted ORF in Assemblies 19 C 20 and 21 B caspofungin induced | orf_classification=Uncharacterized | CNE1,orf19.12759,IPF18102.2,IPF8537.2,Contig4-2961_0019,Contig4-2961_0020,Contig4-2961_0021,orf6.3934,orf6.8288,CA2157,CaO19.5300,IPF8537,CAWG_03398 |
| orf19.3968 | orf19.3968 |  | chr5 | 1051721 | 1052032 | Biofilm- and planktonic growth-induced gene | orf_classification=Dubious | orf19.11451,IPF23347.1,IPF28582.1,orf6.2745,CaO19.3968,CaO19.11451,CAWG_04848 |
| orf19.4326 | orf19.4326 |  | chr5 | 664935 | 666956 | Ortholog 8s 9 have role in mRNA cis splicing C via spliceosome C maturation of 5S rRNA and U4 FU6 x U5 tri-snRNP complex localization | orf_classification=Uncharacterized | SNU66,orf19.11801,IPF22949.1,IPF16019.2,Contig4-2180_0001,orf6.3260,CA1656,CaO19.11801,CaO19.4326,IPF16019,CAWG_04682 |
| orf19.4342 | orf19.4342 |  | chr5 | 697397 | 698467 | Biofilm-induced gene | orf_classification=Uncharacterized | orf19.11817,IPF11357.2,IPF14623.2,Contig4-2344_0008,orf6.1848,orf6.2799,CA1344,CaO19.4342,IPF14623,CAWG_04694 |
| orf19.2645 | orf19.2645 |  | chr5 | 760524 | 760844 | Ortholog of Candida albicans WO-1 A CAWG_04710 | orf_classification=Dubious | orf19.10168,IPF24704.1,IPF29040.1,orf6.3818,CaO19.2645,CaO19.10168,CAWG_04710 |
| orf19.948 | orf19.948 |  | chr5 | 77014 | 77355 | Ortholog of Candida dubliniensis CD36 A CD36_50425 and Candida albicans WO-1 A CAWG_04435 | orf_classification=Dubious | orf19.8563,IPF26360.1,IPF29626.1,Contig4-3103_0045,orf6.8197,CaO19.8563,CaO19.948,CAWG_04435 |
| orf19.2638 | orf19.2638 |  | chr5 | 772041 | 772868 | Ortholog of C. parapsilosis CDC317 A CPAR2_502060 C Candida tenuis NRRL Y-1498 A CANTEDRAFT_115234 C Candida dubliniensis CD36 A CD36_53200 and Pichia stipitis Pignal A PICST_32115 | orf_classification=Uncharacterized | orf19.10161,IPF6967.2,IPF18967.2,Contig4-2912_0019,orf6.1459,orf6.3825,CA2073,CaO19.2638,IPF6967,CAWG_04718 |
| orf19.3210 | orf19.3210 |  | chr5 | 882434 | 883072 | Predicted ORF in Assemblies 19 C 20 and 21 B Plc1p-regulated | orf_classification=Uncharacterized | orf19.10722,IPF24100.1,IPF12399.2,Contig4-2463_0004,orf6.1700,CA0648,CaO19.10722,CaO19.3210,IPF12399,CAWG_04773 |
| orf19.4196 | orf19.4196 |  | chr6 | 111222 | 111554 | Ortholog of Candida dubliniensis CD36 A CD36_60525 and Candida albicans WO-1 A CAWG_05297 | orf_classification=Dubious | IPF18710.1,orf6.2739,CaO19.4196,CAWG_05297 |
| orf19.692 | orf19.692 |  | chr6 | 414704 | 416044 | Hap43p-repressed gene | orf_classification=Uncharacterized | PEX20,orf19.8311,orf19.11003,IPF23773.1,IPF12234.2,IPF26641.1,IPF11987.1,Contig4-2418_0008,orf6.1983,orf6.4369,CA2459,CA0823,orf19.3509,CaO19.692,CaO19.8311,IPF11987.3eoc,CAWG_05165 |
| orf19.3508 | orf19.3508 |  | chr6 | 416316 | 417449 | Putative protein of unknown function B stationary phase enriched protein | orf_classification=Uncharacterized | orf19.11002,IPF23767.1,IPF12233.1,Contig4-2540_0001,orf6.4368,CA2458,CaO19.3508,CaO19.11002,IPF12233,CAWG_05164 |
| orf19.3503 | orf19.3503 |  | chr6 | 425580 | 425888 | Ortholog of Candida albicans WO-1 A CAWG_05160 | orf_classification=Dubious | orf19.10997,IPF23774.1,IPF28752.1,orf6.4363,CaO19.10997,CaO19.3503,CAWG_05160 |
| orf19.5747 | orf19.5747 |  | chr6 | 807460 | 808842 | Ortholog 8s 9 have structural constituent of ribosome activity and mitochondrial small ribosomal subunit localization | orf_classification=Uncharacterized | MRP4,orf19.13170,IPF15124.2,IPF27934.1,Contig4-2858_0007,orf6.2926,CA1428,CaO19.5747,CaO19.13170,CAWG_05000 |
| orf19.6897 | orf19.6897 |  | chr7 | 236122 | 236562 | Protein of unknown function | orf_classification=Uncharacterized | Alias=IPF2239.1,Contig4-2914_0012,Contig4-2965_0012,orf6.7161,CaJ7_0131,CaO19_7003,CaO19.6897 |
| orf19.6580 | orf19.6580 |  | chr7 | 312572 | 313264 | Ortholog of C. parapsilosis CDC317 A CPAR2_703400 C Candida tenuis NRRL Y-1498 A CANTEDRAFT_93186 C Debaryomyces hansenii CBS767 A DEHA2F10142g and Candida dubliniensis CD36 A CD36_71330 | orf_classification=Uncharacterized | orf19.13933,IPF20635.1,IPF1631.2,Contig4-2945_0012,Contig4-3066_0007,orf6.7568,CA4888,CaJ7.0164,CaO19.6580,CaO19_6580,CaJ7_0164,CaO19.13933,IPF1631,CAWG_05500 |
| orf19.6556 | orf19.6556 |  | chr7 | 363743 | 364477 | Protein of unknown function B late-stage biofilm-induced gene | orf_classification=Uncharacterized | orf19.13909,IPF19569.2,IPF19568.1,orf6.330,orf6.7544,CA4870,CaJ7.0190,CaJ7_0190,CaO19_6556,CaO19.6556,IPF19568,CAWG_05519 |
| orf19.5136 | orf19.5136 |  | chr7 | 693471 | 694157 | Putative pyridoxamine 5 7-phosphate oxidase B early-stage biofilm- and planktonic growth-induced gene | orf_classification=Uncharacterized | orf19.12601,IPF22064.1,IPF9255.2,Contig4-2447_0008,orf6.5962,CA3637,CaJ7.0368,CaO19_5136,CaJ7_0368,CaO19.12601,CaO19.5136,IPF9255,CAWG_05653 |
| orf19.1804 | orf19.1804 |  | chrR | 1064655 | 1065743 | Ortholog 8s 9 have mitochondrion localization | orf_classification=Uncharacterized | orf19.9370,IPF18938.1,IPF14864.1,Contig4-2476_0004,orf6.1161,orf6.1577,CA0572,CaO19.1804,IPF14864,CAWG_01826 |
| orf19.5284 | orf19.5284 |  | chrR | 1160082 | 1160396 | Ortholog of Candida albicans WO-1 A CAWG_01866 | orf_classification=Dubious | IPF27500.1,IPF27469.1,orf6.1943,orf19.3510,CaO19.5284,CAWG_01866 |
| orf19.1852 | orf19.1852 |  | chrR | 1462928 | 1463452 | Ortholog 8s 9 have mitochondrion localization | orf_classification=Uncharacterized | orf19.9410,IPF25475.1,IPF4674.2,Contig4-3021_0019,Contig4-3021_0020,orf6.5619,CA3370,CaO19.1852,CaO19.9410,IPF4674,CAWG_01981 |
| orf19.1844 | orf19.1844 |  | chrR | 1470656 | 1472911 | Protein similar to ferric reductase Fre10p | orf_classification=Uncharacterized | FRE4,CFL91,FRE43,orf19.9403,IPF4664.2,IPF29287.1,IPF19399.2,Contig4-3021_0032,orf6.184,orf6.5612,CA6264,CA3366,orf19.1845,FRE43.5f,CAWG_01986 |
| orf19.8 | orf19.8 |  | chrR | 1550784 | 1552991 | Ortholog of C. parapsilosis CDC317 A CPAR2_204290 C Candida tenuis NRRL Y-1498 A CANTEDRAFT_121459 C Debaryomyces hansenii CBS767 A DEHA2E03938g and Candida dubliniensis CD36 A CD36_32690 | orf_classification=Uncharacterized | orf19.7681,IPF11570.2,IPF29984.1,orf6.506,CA0099,CaO19.8,IPF11570.5eoc,CAWG_02009 |
| orf19.609 | orf19.609 |  | chrR | 1725648 | 1725983 | Predicted ORF in Assemblies 19 C 20 and 21 B transcription is specific to white cell type | orf_classification=Dubious | IPF29770.1,orf6.2979,CaO19.609,CAWG_02084 |
| orf19.3238 | orf19.3238 |  | chrR | 269104 | 269505 | Ortholog of Candida albicans WO-1 A CAWG_01469 | orf_classification=Dubious | orf19.10748,IPF24066.1,IPF28832.1,Contig4-2605_0004,orf6.8238,CaO19.10748,CaO19.3238,CAWG_01469 |
| orf19.2818 | orf19.2818 |  | chrR | 578073 | 581285 | Ortholog 8s 9 have RNA-dependent ATPase activity C second spliceosomal transesterification activity C role in generation of catalytic spliceosome for second transesterification step and U2-type catalytic step 2 spliceosome localization | orf_classification=Uncharacterized | PRP16,orf19.10336,IPF24524.1,IPF19698.1,Contig4-2405_0001,Contig4-2405_0002,orf6.1526,CA0537,CaO19.10336,CaO19.2818,CAWG_01599 |
| orf19.2399 | orf19.2399 |  | chrR | 726438 | 728183 | Putative transcription factor with zinc finger DNA-binding motif B similar to bacterial DnaJ B transcriptionally regulated by iron B expression greater in low iron | orf_classification=Uncharacterized | JJJ1,orf19.9935,orf6.229,IPF14284.2,IPF15229.2,Contig4-2658_0008,orf6.3674,CA1961,CaO19.2399,IPF14284,CAWG_01659 |

Gene Ontology of stopped genes by Gene Ontology Term Finder tool on Candida Genome Database (CGD).

| GO_ID | GO_term | | Cluster frequency | Adj pvalue | FDR | Gene(s) annotated to the term | Directly Annotated GOID List |
| --- | --- | --- | --- | --- | --- | --- | --- |
| 50789 | regulation of biological process | 24 out of 46 genes, 52.2% | | 0.00171 | 0.00% | TAF145:CLA4:DBF2:LTE1:PST1:KIS1:FKH2:SMC3:GDA1:MDS3:MLP1:ARG83:DCR1:CWH41:CDC68:NAM7:ZCF20:HAL9:EST1:GRX1:SEF1:PEX14:RGS2:PGA13 | 60261:34473:31028:6261:1410:36178:1900439:6256:1900445:10629:38032:90055:30447:34244:71406:48315:7623:43457:32463:7004:45454:9272:7052:6338:61428:6325:6486:11:6468:51056:42174:7035:71467:45899:34605:43254:1900436:16233:45892:34964:7188:17148:36244:30847:6368:6334:44182:7155:70785:31929:16567:32005:46712:9311:42307:34476:10811:280:6355:6351:184:35376:30466:52559:32784:9267:7130:44011:2000221:7264:60257:10570:6606:51301:36177:70783:30422:6109:36171:36170:34398:6879:1901925:9405:16075:51123:71469:30448:7131:6635:90204:44114:70887:8298:70478:71216:51278:902:33212:42268:51177:34963:9303:6310:7163:6487:36187:6562:31505:60237:30437:910:44117:16560:36168:36180:61167:71930:34475:31106:32880:31031:6369:16973:6493:19236:32298:90419:7124:71169:2000720:6364:34401:1403:7165:436:90203:35196:90282:7535:31124:6379:6357:35690:7096:71048:34724:7064:16192 |
| 50794 | regulation of cellular process | 22 out of 46 genes, 47.8% | | 0.00626 | 0.00% | TAF145:CLA4:DBF2:LTE1:PST1:KIS1:FKH2:SMC3:MDS3:MLP1:ARG83:DCR1:CDC68:NAM7:ZCF20:HAL9:EST1:GRX1:SEF1:PEX14:RGS2:PGA13 | 60261:34473:31028:6261:1410:1900439:1900445:10629:38032:90055:30447:34244:71406:48315:7623:43457:32463:7004:45454:7052:6338:61428:6325:11:6468:51056:42174:7035:71467:45899:34605:43254:1900436:16233:45892:34964:7188:17148:30847:6368:6334:44182:7155:70785:31929:16567:32005:42307:34476:10811:280:6355:6351:184:35376:30466:32784:9267:7130:44011:2000221:7264:60257:10570:6606:51301:36177:70783:30422:6109:36171:36170:34398:6879:1901925:9405:16075:51123:71469:7131:30448:6635:90204:70887:44114:8298:70478:71216:902:33212:42268:51177:34963:6310:9303:7163:6562:31505:60237:30437:910:44117:16560:36168:36180:61167:34475:71930:31106:32880:31031:6369:16973:19236:32298:90419:7124:71169:6364:2000720:34401:1403:7165:436:90203:35196:90282:7535:6379:31124:6357:35690:34724:71048:7096:7064:16192 |
| 48519 | negative regulation of biological process | 14 out of 46 genes, 30.4% | | 0.00703 | 0.00% | CLA4:DBF2:PST1:FKH2:MDS3:MLP1:DCR1:NAM7:ZCF20:HAL9:EST1:PEX14:RGS2:PGA13 | 34473:31028:1410:1900439:1900445:10629:38032:90055:30447:34244:71406:48315:7623:43457:32463:7004:7052:6338:61428:6325:11:6468:42174:7035:71467:34605:1900436:16233:45892:34964:7188:17148:30847:44182:70785:31929:16567:32005:42307:34476:10811:280:6355:184:35376:30466:9267:44011:2000221:60257:10570:6606:51301:36177:70783:30422:36171:36170:34398:1901925:9405:16075:30448:6635:90204:70887:44114:8298:70478:71216:902:42268:34963:6310:9303:7163:31505:60237:30437:910:16560:36168:36180:61167:34475:71930:31106:32880:31031:6369:16973:19236:32298:90419:7124:71169:6364:436:90203:35196:90282:7535:6379:31124:6357:35690:71048:7096 |
| 48468 | cell development | 7 out of 46 genes, 15.2% | | 0.00839 | 0.00% | PGA5:CLA4:SMC3:MDS3:SPO75:UGT51C1:PEX14 | 1410:1900439:1900445:10629:30447:48315:11:6468:30476:42174:71467:34605:1900436:45892:44182:31929:32005:42307:10811:35376:30259:9267:7130:44011:10570:36177:36171:36170:9405:7131:5975:6635:70887:44114:71216:42268:51177:7163:30437:16560:36168:32120:36180:31106:19236:7124:2000720:35690:16125:7096:7064 |
| 30154 | cell differentiation | 8 out of 46 genes, 17.4% | | 0.00858 | 0.00% | PGA5:CLA4:FKH2:SMC3:MDS3:SPO75:UGT51C1:PEX14 | 1410:1900439:1900445:10629:90055:30447:34244:71406:48315:6338:11:6468:30476:42174:71467:34605:1900436:45892:44182:31929:32005:42307:10811:35376:30259:9267:7130:44011:2000221:10570:36177:36171:36170:9405:7131:5975:6635:70887:44114:71216:902:42268:51177:7163:30437:16560:36168:32120:36180:71930:31106:6369:19236:32298:90419:7124:2000720:90282:7535:31124:6357:35690:16125:7096:7064 |
| 65007 | biological regulation | 24 out of 46 genes, 52.2% | | 0.01360 | 0.00% | TAF145:CLA4:DBF2:LTE1:PST1:KIS1:FKH2:SMC3:GDA1:MDS3:MLP1:ARG83:DCR1:CWH41:CDC68:NAM7:ZCF20:HAL9:EST1:GRX1:SEF1:PEX14:RGS2:PGA13 | 60261:34473:31028:6261:1410:36178:1900439:6256:1900445:10629:38032:90055:30447:34244:71406:48315:7623:43457:32463:7004:45454:9272:7052:6338:61428:6325:6486:11:6468:51056:42174:7035:71467:45899:34605:43254:1900436:16233:45892:34964:7188:17148:36244:30847:6368:6334:44182:7155:70785:31929:16567:32005:46712:9311:42307:34476:10811:280:6355:6351:184:35376:30466:52559:32784:9267:7130:44011:2000221:7264:60257:10570:6606:51301:36177:70783:30422:6109:36171:36170:34398:6879:1901925:9405:16075:51123:71469:7131:30448:6635:90204:70887:44114:8298:70478:51278:71216:902:33212:42268:51177:34963:6310:9303:7163:6487:36187:6562:31505:60237:30437:910:44117:16560:36168:36180:61167:34475:71930:31106:32880:31031:6369:16973:6493:19236:32298:90419:7124:71169:6364:2000720:34401:1403:7165:436:90203:35196:90282:7535:6379:31124:6357:35690:34724:71048:7096:7064:16192 |
| 30435 | sporulation resulting in formation of a cellular spore | 7 out of 46 genes, 15.2% | | 0.02322 | 0.00% | PGA5:CLA4:SMC3:MDS3:SPO75:UGT51C1:PEX14 | 1410:1900439:1900445:10629:30447:48315:11:6468:30476:42174:71467:34605:1900436:45892:44182:31929:32005:42307:10811:35376:30259:9267:7130:44011:10570:36177:36171:36170:9405:7131:5975:6635:70887:44114:71216:42268:51177:7163:30437:16560:36168:32120:36180:31106:19236:7124:2000720:35690:16125:7096:7064 |
| 48869 | cellular developmental process | 8 out of 46 genes, 17.4% | | 0.03369 | 0.00% | PGA5:CLA4:FKH2:SMC3:MDS3:SPO75:UGT51C1:PEX14 | 1410:1900439:1900445:10629:90055:30447:34244:71406:48315:6338:11:6468:30476:42174:71467:34605:1900436:45892:44182:31929:32005:42307:10811:35376:30259:9267:7130:44011:2000221:10570:36177:36171:36170:9405:7131:5975:6635:70887:44114:71216:902:42268:51177:7163:30437:16560:36168:32120:36180:71930:31106:6369:19236:32298:90419:7124:2000720:90282:7535:31124:6357:35690:16125:7096:7064 |
| 9653 | anatomical structure morphogenesis | 8 out of 46 genes, 17.4% | | 0.03369 | 0.00% | PGA5:CLA4:FKH2:SMC3:MDS3:SPO75:UGT51C1:PEX14 | 1410:1900439:1900445:10629:90055:30447:34244:71406:48315:6338:11:6468:30476:42174:71467:34605:1900436:45892:44182:31929:32005:42307:10811:35376:30259:9267:7130:44011:2000221:10570:36177:36171:36170:9405:7131:5975:6635:70887:44114:71216:902:42268:51177:7163:30437:16560:36168:32120:36180:71930:31106:6369:19236:32298:90419:7124:2000720:90282:7535:31124:6357:35690:16125:7096:7064 |
| 48856 | anatomical structure development | 8 out of 46 genes, 17.4% | | 0.06032 | 0.60% | PGA5:CLA4:FKH2:SMC3:MDS3:SPO75:UGT51C1:PEX14 | 1410:1900439:1900445:10629:90055:30447:34244:71406:48315:6338:11:6468:30476:42174:71467:34605:1900436:45892:44182:31929:32005:42307:10811:35376:30259:9267:7130:44011:2000221:10570:36177:36171:36170:9405:7131:5975:6635:70887:44114:71216:902:42268:51177:7163:30437:16560:36168:32120:36180:71930:31106:6369:19236:32298:90419:7124:2000720:90282:7535:31124:6357:35690:16125:7096:7064 |
| 43934 | sporulation | 7 out of 46 genes, 15.2% | | 0.07641 | 0.55% | PGA5:CLA4:SMC3:MDS3:SPO75:UGT51C1:PEX14 | 1410:1900439:1900445:10629:30447:48315:11:6468:30476:42174:71467:34605:1900436:45892:44182:31929:32005:42307:10811:35376:30259:9267:7130:44011:10570:36177:36171:36170:9405:7131:5975:6635:70887:44114:71216:42268:51177:7163:30437:16560:36168:32120:36180:31106:19236:7124:2000720:35690:16125:7096:7064 |
| 48646 | anatomical structure formation involved in morphogenesis | 7 out of 46 genes, 15.2% | | 0.08201 | 0.67% | PGA5:CLA4:SMC3:MDS3:SPO75:UGT51C1:PEX14 | 1410:1900439:1900445:10629:30447:48315:11:6468:30476:42174:71467:34605:1900436:45892:44182:31929:32005:42307:10811:35376:30259:9267:7130:44011:10570:36177:36171:36170:9405:7131:5975:6635:70887:44114:71216:42268:51177:7163:30437:16560:36168:32120:36180:31106:19236:7124:2000720:35690:16125:7096:7064 |
| 30447 | filamentous growth | 13 out of 46 genes, 28.3% | | 0.08796 | 0.62% | CLA4:DBF2:KIS1:FKH2:FGR44:GDA1:MDS3:ARG83:CWH41:NAM7:HAL9:SEF1:PGA13 | 31028:1410:36178:1900439:6256:1900445:10629:90055:30447:34244:71406:7623:32463:9272:7052:6338:6486:11:6468:42174:7035:71467:34605:43254:1900436:36244:44182:7155:70785:31929:16567:32005:46712:9311:42307:10811:280:6355:6351:184:35376:30466:52559:9267:44011:2000221:60257:10570:36177:70783:6109:36171:36170:6879:9405:6012:71469:30448:70887:44114:8298:70478:51278:71216:902:33212:6310:7163:6487:36187:6562:31505:910:44117:36168:36180:61167:71930:31106:32880:31031:6369:6493:19236:32298:90419:7124:1403:7165:90282:7535:31124:6357:35690:7096 |

**Supplementary Table 6. Summary of out-of-frame mutations in the whole genome sequenced YL1 isolate.** Information about genes, including ORF number, systematic and standard name and description, as well as ORF position in the *C. albicans* SC5314 reference strain are reported.

| ORF position (chr) in SC5314 reference strain | | | ORF | Parent ORF | Gene | Description | Note | Alias |
| --- | --- | --- | --- | --- | --- | --- | --- | --- |
| chr1 | 1340369 | 1340722 | orf19.6280 | orf19.6280 |  | Dubious open reading frame | orf_classification=Dubious | orf19.13659,IPF20954.1,IPF27787.1,orf6.6039,CaO19.13659,CaO19.6280 |
| chr1 | 2600273 | 2601934 | orf19.5280 | orf19.5280 | MUP1 | Putative high affinity methionine permease B alkaline upregulated by Rim101p B biofilm induced | orf_classification=Uncharacterized | orf19.12745,IPF21939.1,IPF4701.2,Contig4-3082_0038,orf6.7662,CA4972,CaO19.5280,CaO19.12745,CAWG_00254 |
| chr2 | 1732194 | 1735709 | orf19.3623 | orf19.3623 | SMC2 | Protein similar to S. cerevisiae Smc2p C which is a component of the condensin complex involved in mitotic chromosome condensation B induced under hydroxyurea treatment | orf_classification=Uncharacterized | orf19.11106,IPF23694.1,IPF11993.2,Contig4-2568_0001,orf6.1515,CA0529,CaO19.3623,CAWG_05940 |
| chr2 | 514977 | 517088 | orf19.1580 | orf19.1580 |  | Ortholog of Candida dubliniensis CD36 A CD36_17050 C Candida guilliermondii ATCC 6260 A PGUG_05142 and Candida albicans WO-1 A CAWG_04023 | orf_classification=Uncharacterized | YFW3,orf19.9153,IPF25755.1,IPF13187.2,Contig4-3005_0023,Contig4-3005_0025,orf6.5340,CA3164,CaO19.1580,CaO19.9153,IPF13187,CAWG_04023 |
| chr2 | 932070 | 933323 | orf19.4506 | orf19.4506 | LYS22 | Putative homocitrate synthase B fungal-specific 8no human or murine homolog 9 B repressed by nitric oxide and by hypoxia B protein level decreases in stationary phase cultures B induced by ketoconazole | orf_classification=Uncharacterized | LYS211,LYS21,orf19.11982,IPF18482.1,IPF15851.2,Contig4-2894_0013,orf6.4198,orf6.5005,CA2335,CaO19.4506,CAWG_04202 |
| chr3 | 1201851 | 1204193 | orf19.6979 | orf19.6979 |  | Ortholog 8s 9 have role in cellular manganese ion homeostasis C mitochondrion organization and fungal-type vacuole membrane localization | orf_classification=Uncharacterized | AMI3,IPF3003.1,Contig4-2646_0008,Contig4-2646_0009,orf6.7725,CA5025,CaO19.6979,CAWG_02854 |
| chr3 | 231364 | 232086 | orf19.2516 | orf19.2516 |  | Has domain 8s 9 with predicted role in cell redox homeostasis | orf_classification=Uncharacterized | orf19.10052,IPF24860.1,IPF18533.1,orf6.3921,CA2147,IPF18533.5eoc,IPF18533,CAWG_02448 |
| chr4 | 1287448 | 1287813 | orf19.1266 | orf19.1266 |  | Predicted ORF in Assemblies 19 C 20 and 21 B transcription detected in high-resolution tiling array experiments | orf_classification=Uncharacterized | orf19.8852,IPF10280.1,IPF14956.3,Contig4-1503_0003,orf6.5733,CA0944,CaO19.1266,IPF10280,orf6.2158,CAWG_03243 |
| chr4 | 1434188 | 1434862 | orf19.2888 | orf19.2888 |  | Ortholog 8s 9 have role in early endosome to Golgi transport C protein complex assembly and TRAPP complex C clathrin-coated vesicle localization | orf_classification=Uncharacterized | orf19.10406,IPF24448.1,IPF10431.2,Contig4-2827_0010,orf6.6134,CA3778,CaO19.2888,CaO19.10406,IPF10431,CAWG_03181 |
| chr4 | 1442224 | 1443855 | orf19.2885 | orf19.2885 | PRI2 | Putative DNA primase B gene adjacent to and divergently transcribed with CDC68 B Hap43p-induced gene | orf_classification=Uncharacterized | orf19.10403,IPF24426.1,IPF14788.3,Contig4-2827_0021,orf6.1820,CA0723,CaO19.10403,CaO19.2885,IPF14788.2,CAWG_03178 |
| chr4 | 1457501 | 1460491 | orf19.2881 | orf19.2881 | MNN4 | Regulator of mannosylphosphorylation of N-linked mannans to cell wall proteins B not required for virulence Fkidney burden in mice or normal interaction with macrophages B mutants induce high levels of inflammatory cytokines in dendritic cells |  | orf19.10399,orf6.4390,IPF16016.2,IPF28958.1,Contig4-2084_0003,orf6.296,CA2477,CaO19.2881,IPF16016,CAWG_03174 |
| chr4 | 584483 | 585841 | orf19.2715 | orf19.2715 | RPC53 | Ortholog 8s 9 have DNA-directed RNA polymerase activity C role in tRNA transcription from RNA polymerase III promoter and DNA-directed RNA polymerase III complex localization | orf_classification=Uncharacterized | orf19.10230,IPF24613.1,IPF19910.1,Contig4-2673_0008,orf6.2704,CA1275,CaO19.2715,CAWG_03518 |
| chr4 | 844616 | 844921 | orf19.787 | orf19.787 |  | Ortholog of Candida albicans WO-1 A CAWG_03417 | orf_classification=Uncharacterized | orf19.8406,IPF26483.1,IPF29675.1,orf6.558,CaO19.787,CaO19.8406,CAWG_03417 |
| chr5 | 280836 | 283307 | orf19.1944 | orf19.1944 | GPR1 | Plasma membrane G-protein-coupled receptor of the cAMP-PKA pathway B required for wild-type hyphal growth B reports differ on role in cAMP-mediated glucose signaling B Gpr1p C terminus binds Gpa2p B regulates HWP1 and ECE1 B biofilm-induced |  | orf19.9499,IPF11281.2,IPF29241.1,Contig4-2985_0021,Contig4-2985_0023,orf6.5595,CA3354,CaO19.1944,CaO19.9499,CA3354 gi A30513635,IPF11281,CAWG_04513 |
| chr5 | 511629 | 513644 | orf19.4239 | orf19.4239 |  | Planktonic growth-induced gene | orf_classification=Uncharacterized | orf19.11714,IPF22950.1,IPF20163.1,Contig4-2209_0003,orf6.6606,CA4135,CaO19.4239,CaO19.11714,IPF20163,CAWG_04613 |
| chr5 | 77014 | 77355 | orf19.948 | orf19.948 |  | Ortholog of Candida dubliniensis CD36 A CD36_50425 and Candida albicans WO-1 A CAWG_04435 | orf_classification=Dubious | orf19.8563,IPF26360.1,IPF29626.1,Contig4-3103_0045,orf6.8197,CaO19.8563,CaO19.948,CAWG_04435 |
| chr5 | 940701 | 941291 | orf19.3906 | orf19.3906 |  | Protein not essential for viability | orf_classification=Uncharacterized | orf19.11387,IPF23302.1,IPF17640.2,orf6.5142,CA3021,CaO19.11387,CaO19.3906,IPF17640,CAWG_04796 |
| chr6 | 13539 | 13784 | orf19.6325.1 | orf19.6325.1 |  | Ortholog 8s 9 have mitochondrion localization | orf_classification=Uncharacterized | IPF5730.1,CA3291,IPF5730,CAWG_05345 |
| chr6 | 718017 | 720212 | orf19.5701 | orf19.5701 |  | Ortholog 8s 9 have role in DNA replication initiation C establishment of mitotic sister chromatid cohesion and condensed nuclear chromosome kinetochore localization | orf_classification=Uncharacterized | orf19.13076,orf19.13124,IPF18416.1,IPF27982.1,IPF21471.1,IPF8472.2,orf6.4790,orf6.4631,CA2766,CA2653,orf19.5631,CaO19.13124,CaO19.5701,IPF8472.3eoc,CAWG_05028 |
| chrR | 1208480 | 1208878 | orf19.6644 | orf19.6644 |  | Biofilm-induced gene B transcription detected in high-resolution tiling array experiments | orf_classification=Uncharacterized | orf19.13965,IPF20528.1,IPF5012.1,orf6.6541,CaO19.13965,CaO19.6644,CAWG_01889 |
| chrR | 1586310 | 1587170 | orf19.6140 | orf19.6140 | FRE30 | Protein with similarity to ferric reductases B downregulated in response to amphotericin B C estradiol C or ciclopirox olamine C and upregulated by interaction with macrophage B un-merged from orf19.6139 in a revision of Assembly 21 | orf_classification=Uncharacterized | orf19.13559,FRE30.53,orf6.5674,CA3415,FRE30.53f,IPF17765.2 |
| chrR | 1637901 | 1638206 | orf19.2625 | orf19.2625 |  | Ortholog of Candida albicans WO-1 A CAWG_02051 | orf_classification=Dubious | orf19.10156,IPF24719.1,IPF29046.1,Contig4-2829_0006,orf6.2649,CaO19.10156,CaO19.2625,CAWG_02051 |

**Supplementary Table 7. Summary of out-of-frame mutations in the whole genome sequenced YQ2 isolate.** Information about genes, including ORF number, systematic and standard name and description, as well as ORF position in the *C. albicans* SC5314 reference strain are reported.

| ORF | Parent ORF | ORF position (chr) in SC5314 reference strain | | | Description | Note | Alias |
| --- | --- | --- | --- | --- | --- | --- | --- |
| orf19.2968 | orf19.2968 | chr1 | 584392 | 584994 | Ortholog of Debaryomyces hansenii CBS767 A DEHA2D07920g C Candida dubliniensis CD36 A CD36_02600 C Pichia stipitis Pignal A PICST_32663 and Spathaspora passalidarum NRRL Y-27907 A SPAPADRAFT_57891 | orf_classification=Uncharacterized | IPF7774.2,IPF28910.1,Contig4-2061_0004,orf6.2121,CA4165,CaO19.2968,IPF7774,orf6.6648,IPF7774.1,CAWG_01104 |
| orf19.1536 | orf19.1536 | chr2 | 433195 | 434631 | Putative vacuolar transporter B Hap43p-induced gene C required for normal filamentous growth B mRNA binds to She3p and is localized to hyphal tips | orf_classification=Verified | orf19.9111,IPF19060.1,Contig4-2088_0003,orf6.1061,orf6.1062,orf6.2308,CA6257,CA1030,orf19.1537,ZRC.5f,CAWG_03987.1 |
| orf19.4515 | orf19.4515 | chr2 | 918785 | 919528 | Predicted ORF in Assemblies 19 C 20 and 21 B possibly an essential gene C disruptants not obtained by UAU1 method | orf_classification=Uncharacterized | CIS303,orf19.11990,IPF11417.1,IPF11206.1,Contig4-2521_0010,orf6.392,orf6.4996,CA2912,CaO19.4515,IPF11206,CAWG_04195 |
| orf19.2512 | orf19.2512 | chr3 | 222909 | 223568 | Ortholog of C. parapsilosis CDC317 A CPAR2_103050 C Candida tenuis NRRL Y-1498 A CANTEDRAFT_116326 C Debaryomyces hansenii CBS767 A DEHA2G01958g and Candida dubliniensis CD36 A CD36_81000 | orf_classification=Uncharacterized | orf19.10048,IPF12803.2,IPF29085.1,Contig4-1978_0003,orf6.3917,CA2143,CaO19.2512,CaO19.10048,IPF12803,CAWG_02444 |
| orf19.2725 | orf19.2725 | chr4 | 566926 | 567228 | Ortholog of Candida albicans WO-1 A CAWG_03525 | orf_classification=Dubious | IPF29033.1,orf6.325,CaO19.2725,CAWG_03525 |
| orf19.5042 | orf19.5042 | chr4 | 821221 | 821859 | Ortholog 8s 9 have role in maintenance of rDNA and nuclear periphery localization | orf_classification=Uncharacterized | orf19.12509,IPF22193.1,IPF16759.2,Contig4-2272_0004,orf6.3319,CaO19.12509,CaO19.5042,CAWG_03425 |
| orf19.4264 | orf19.4264 | chr5 | 562920 | 563825 | Biofilm-induced gene | orf_classification=Uncharacterized | orf19.11740,IPF9520.1,IPF19069.2,Contig4-2550_0015,orf6.1033,orf6.3493,CA1827,CaO19.11740,CaO19.4264,IPF9520,CAWG_04634 |
| orf19.1105.3 | orf19.1105.3 | chr5 | 855473 | 855730 | Ortholog of Candida albicans WO-1 A CAWG_04761 | orf_classification=Uncharacterized | IPF26207.1,IPF20047.1,Contig4-2815_0006,orf6.2981,CAWG_04761 |
| orf19.3219 | orf19.3219 | chr5 | 859577 | 861382 | Ortholog 8s 9 have role in proton transport | orf_classification=Uncharacterized | orf19.10730,IPF24094.1,IPF14538.2,Contig4-2205_0004,Contig4-2205_0005,orf6.3200,CA1616,CaO19.3219,CaO19.10730,IPF14538,CAWG_04765 |
| orf19.3904 | orf19.3904 | chr5 | 937242 | 938012 | Ortholog of C. parapsilosis CDC317 A CPAR2_502450 C Lodderomyces elongisporus NRLL YB-4239 A LELG_04018 C Candida dubliniensis CD36 A CD36_53900 and Pichia stipitis Pignal A PICST_29703 | orf_classification=Uncharacterized | orf19.11385,IPF23281.1,IPF11508.2,orf6.5144,CA3023,CaO19.11385,CaO19.3904,IPF11508,CAWG_04794 |
| orf19.2163 | orf19.2163 | chr6 | 1028818 | 1031331 | Ortholog 8s 9 have cytosol localization | orf_classification=Uncharacterized | orf19.9709,IPF25178.1,IPF2702.1,orf6.2476,CA1134,CaO19.2163,CaO19.9709,IPF2702,CAWG_04918 |
| orf19.7108 | orf19.7108 | chr7 | 19179 | 19871 | D-ribulose-5-phosphate 3-epimerase B stationary phase enriched protein | orf_classification=Uncharacterized | RPE1,IPF5616.1,Contig4-2995_0027,orf6.8445,CA5592,CaJ7.0022,CaO19.7108,CaJ7_0022,CaO19_7108,CAWG_05372 |
| orf19.6493 | orf19.6493 | chr7 | 483239 | 483631 | Ortholog of Candida dubliniensis CD36 A CD36_72020 and Candida albicans WO-1 A CAWG_05570 | orf_classification=Uncharacterized | orf19.13846,IPF8448.2,IPF19356.2,Contig4-2822_0001,orf6.3358,CA1727,CaJ7.0257,CaO19.6493,CaO19_6493,CaJ7_0257,IPF8448,CAWG_05570 |
| orf19.6465 | orf19.6465 | chr7 | 526552 | 527907 | Ortholog of Candida dubliniensis CD36 A CD36_83910 C Candida tropicalis MYA-3404 A CTRG_05087 and Candida albicans WO-1 A CAWG_00654 | orf_classification=Uncharacterized | IPF17991.2,Contig4-2630_0004,orf6.5994,CA3661,CaJ7.0286,CaO19.6465,IPF17991,CAWG_00654 |
| orf19.711 | orf19.711 | chrR | 1396726 | 1397067 | Predicted ORF from Assembly 19 B induced by nitric oxide B removed from Assembly 20 B restored based on transcription data | orf_classification=Uncharacterized | orf6.2632,orf6.522,CA1234,CaO19.711,IPF18732,IPF18732.1 |
| orf19.1841 | orf19.1841 | chrR | 1484684 | 1485808 | Hap43p-induced gene | orf_classification=Uncharacterized | orf19.9399,IPF17745.2,IPF16028.2,Contig4-2086_0001,orf6.119,orf6.1253,CA0383,CaO19.1841,IPF16028,CAWG_01989 |

**Supplementary Table 8. The complete list of DEGs is reported for each comparison.**

The table is provided separately as an excel file

**Supplementary Table 9. Functional enrichment over Gene Ontology Biological Process categories using DAVID tool.** Adjusted P value (Bonferroni correction) is reported.

| ***Biological process enrichment "YL1 vs YQ2 - FBS 1h" (down)*** |  |
| --- | --- |
| *Term* | *Pvalue (bonferroni correction)* |
| *GO:0022402~cell cycle process* | 6.21E-05 |
| GO:0048610~reproductive cellular process | 7.39E-05 |
| *GO:0022403~cell cycle phase* | 1.07E-04 |
| GO:0000279~M phase | 1.13E-04 |
| *GO:0007049~cell cycle* | 1.61E-04 |
| GO:0007017~microtubule-based process | 2.33E-04 |
| GO:0000747~conjugation with cellular fusion | 2.67E-04 |
| GO:0019953~sexual reproduction | 3.41E-04 |
| GO:0000746~conjugation | 3.99E-04 |
| GO:0000226~microtubule cytoskeleton organization | 4.10E-04 |
| GO:0019236~response to pheromone | 4.73E-04 |
| GO:0032392~DNA geometric change | 6.86E-04 |
| GO:0051327~M phase of meiotic cell cycle | 6.88E-04 |
| GO:0007126~meiosis | 6.88E-04 |
| GO:0000755~cytogamy | 8.08E-04 |
| GO:0051321~meiotic cell cycle | 9.15E-04 |
| GO:0007097~nuclear migration | 0.001463333 |
| GO:0040023~establishment of nucleus localization | 0.001726308 |
| GO:0051647~nucleus localization | 0.001726308 |
| GO:0007127~meiosis I | 0.002254827 |
| *GO:0070726~cell wall assembly* | 2.95E-03 |
| GO:0010033~response to organic substance | 3.07E-03 |
| GO:0000278~mitotic cell cycle | 3.09E-03 |
| GO:0007059~chromosome segregation | 3.35E-03 |
| GO:0006259~DNA metabolic process | 5.11E-03 |
| GO:0022413~reproductive process in single-celled organism | 5.31E-03 |
| GO:0032508~DNA duplex unwinding | 0.005750936 |
| *GO:0009272~fungal-type cell wall biogenesis* | 0.006020492 |
| GO:0030473~nuclear migration along microtubule | 0.007935683 |
| GO:0010970~microtubule-based transport | 0.007935683 |
| GO:0007010~cytoskeleton organization | 9.32E-03 |
|  |  |
| ***Biological process enrichment "YL1 vs YQ2 - FBS 1h" (up)*** |  |
| *Term* | *PValue* |
| *GO:0019216~regulation of lipid metabolic process* | 0.001459378 |
| *GO:0045834~positive regulation of lipid metabolic process* | 0.00280716 |
| GO:0045893~positive regulation of transcription, DNA-dependent | 0.002895322 |
| GO:0051254~positive regulation of RNA metabolic process | 0.003840984 |
| GO:0045449~regulation of transcription | 0.005303872 |
| *GO:0016192~vesicle-mediated transport* | 0.005557125 |
| GO:0045941~positive regulation of transcription | 0.006697472 |
| GO:0010628~positive regulation of gene expression | 0.00700778 |
| GO:0010551~regulation of specific transcription from RNA polymerase II promoter | 0.008939405 |
| GO:0051235~maintenance of location | 0.009044813 |
| GO:0045944~positive regulation of transcription from RNA polymerase II promoter | 0.009944929 |
|  |  |
| ***Biological process enrichment "YL1 vs YQ2 - FBS 24h" (down)*** |  |
| *Term* | *PValue* |
| GO:0006396~RNA processing | 0.00125793 |
| GO:0022613~ribonucleoprotein complex biogenesis | 0.004798 |
| GO:0006364~rRNA processing | 0.009072225 |
| GO:0016072~rRNA metabolic process | 0.010561167 |
|  |  |
| ***Biological process enrichment "YL1 vs YQ2 - FBS 24h" (up)*** |  |
|  |  |
| *Term* | *PValue* |
| GO:0006511~ubiquitin-dependent protein catabolic process | 1.81E-06 |
| GO:0019941~modification-dependent protein catabolic process | 7.37E-06 |
| GO:0051603~proteolysis involved in cellular protein catabolic process | 1.16E-05 |
| GO:0043632~modification-dependent macromolecule catabolic process | 1.78E-05 |
| GO:0030163~protein catabolic process | 8.68E-05 |
| GO:0006508~proteolysis | 1.18E-04 |
| *GO:0016192~vesicle-mediated transport* | 1.30E-04 |
| GO:0009057~macromolecule catabolic process | 1.47E-04 |
| GO:0044257~cellular protein catabolic process | 1.93E-04 |
| GO:0044265~cellular macromolecule catabolic process | 7.22E-04 |
| *GO:0006890~retrograde vesicle-mediated transport, Golgi to ER* | 0.013466376 |
| GO:0006888~ER to Golgi vesicle-mediated transport | 0.01576139 |
| GO:0046907~intracellular transport | 0.02263552 |
|  |  |
| ***Biological process enrichment "YL1 vs YQ2 + FBS 1h" (down)*** |  |
| *Term* | *PValue* |
| *GO:0022403~cell cycle phase* | 0.001622974 |
| *GO:0022402~cell cycle process* | 0.001935684 |
| GO:0032505~reproduction of a single-celled organism | 0.003057703 |
| GO:0022413~reproductive process in single-celled organism | 0.004645509 |
| GO:0000278~mitotic cell cycle | 0.006617619 |
| GO:0007049~cell cycle | 0.007290461 |
| GO:0000755~cytogamy | 0.015291083 |
| GO:0048610~reproductive cellular process | 0.015866073 |
| GO:0000279~M phase | 0.028774149 |
| GO:0034727~piecemeal microautophagy of nucleus | 0.034083116 |
| GO:0006914~autophagy | 0.03538269 |
|  |  |
| ***Biological process enrichment "YL1 vs YQ2 + FBS 1h" (up)*** |  |
| *Term* | *PValue* |
| GO:0006396~RNA processing | 7.10E-06 |
| GO:0034660~ncRNA metabolic process | 2.87E-05 |
| GO:0034470~ncRNA processing | 1.10E-04 |
| GO:0022613~ribonucleoprotein complex biogenesis | 3.19E-04 |
| GO:0042254~ribosome biogenesis | 6.01E-04 |
| GO:0016072~rRNA metabolic process | 0.001380342 |
| GO:0006364~rRNA processing | 0.003049625 |
| GO:0006641~triglyceride metabolic process | 0.005818435 |
| GO:0000462~maturation of SSU-rRNA from tricistronic rRNA transcript (SSU-rRNA, 5,8S rRNA, LSU-rRNA) | 0.006478893 |
| GO:0000469~cleavages during rRNA processing | 0.006592295 |
| GO:0000447~endonucleolytic cleavage in ITS1 to separate SSU-rRNA from 5,8S rRNA and LSU-rRNA from tricistronic rRNA transcript (SSU-rRNA, 5,8S rRNA, LSU-rRNA) | 0.00698291 |
| GO:0030490~maturation of SSU-rRNA | 0.007267345 |
| GO:0006639~acylglycerol metabolic process | 0.008038266 |
| GO:0006638~neutral lipid metabolic process | 0.008038266 |
| GO:0000478~endonucleolytic cleavages during rRNA processing | 0.008382493 |
| GO:0000479~endonucleolytic cleavage of tricistronic rRNA transcript (SSU-rRNA, 5,8S rRNA, LSU-rRNA) | 0.008382493 |
| GO:0000054~ribosome export from nucleus | 0.010807607 |
|  |  |
| ***Biological process enrichment "YL1 vs YQ2 + FBS 24h" (down)*** |  |
| *Term* | *PValue* |
| *GO:0046907~intracellular transport* | 1.93E-05 |
| GO:0070727~cellular macromolecule localization | 1.08E-04 |
| GO:0006886~intracellular protein transport | 1.21E-04 |
| GO:0034613~cellular protein localization | 1.85E-04 |
| GO:0009163~nucleoside biosynthetic process | 4.87E-04 |
| GO:0042455~ribonucleoside biosynthetic process | 4.87E-04 |
| GO:0008104~protein localization | 5.37E-04 |
| GO:0045184~establishment of protein localization | 5.94E-04 |
| GO:0043094~cellular metabolic compound salvage | 9.43E-04 |
| GO:0015031~protein transport | 0.001111358 |
| *GO:0006417~regulation of translation* | *0.001557433* |
| *GO:0016192~vesicle-mediated transport* | *0.002080955* |
| GO:0010608~posttranscriptional regulation of gene expression | 0.002689508 |
| GO:0032268~regulation of cellular protein metabolic process | 0.003920179 |
| GO:0042254~ribosome biogenesis | 0.005582625 |
| GO:0048193~Golgi vesicle transport | 0.005653932 |
| GO:0046129~purine ribonucleoside biosynthetic process | 0.005829502 |
| GO:0042451~purine nucleoside biosynthetic process | 0.005829502 |
| *GO:0043174~nucleoside salvage* | *0.005829502* |
| GO:0006166~purine ribonucleoside salvage | 0.005829502 |
| *GO:0006888~ER to Golgi vesicle-mediated transport* | *0.005904149* |
| *GO:0006890~retrograde vesicle-mediated transport, Golgi to ER* | *0.00602851* |
| GO:0022613~ribonucleoprotein complex biogenesis | 0.008570157 |
| GO:0051169~nuclear transport | 0.01082432 |
| GO:0006913~nucleocytoplasmic transport | 0.01082432 |
|  |  |
| ***Biological process enrichment "YL1 vs YQ2 + FBS 24h" (up)*** |  |
| *Term* | *PValue* |
| GO:0006261~DNA-dependent DNA replication | 0.013932951 |
| *GO:0030447~filamentous growth* | *0.031679725* |
| GO:0006577~betaine metabolic process | 0.031698477 |
| *GO:0009437~carnitine metabolic process* | *0.031698477* |
| GO:0006352~transcription initiation | 0.032787479 |
| GO:0045449~regulation of transcription | 0.040474332 |

**Supplementary Table 10. Functional enrichment over Gene Ontology Biological Process, FunCat categories and KEGG pathways using FungiFun 2.0 tool.** Adjusted P value (Bonferroni correction) is reported.

| ***GO Biological process enrichment "YL1 vs YQ2 - FBS 1h" (down)*** | |  |
| --- | --- | --- |
| *GO ID* | *GO name* | *Exact p-value* |
| GO:0008150 | biological_process | 2.557e-7 |
| GO:0007059 | chromosome segregation | 0.002033 |
| GO:0051382 | kinetochore assembly | 0.006927 |
| *GO:0016042* | *lipid catabolic process* | *0.009963* |
| GO:0015074 | DNA integration | 0.01427 |
| GO:0000398 | mRNA splicing, via spliceosome | 0.018348 |
| *GO:0000045* | *autophagic vacuole assembly* | *0.021626* |
| *GO:1900443* | *regulation of filamentous growth of a population of unicellular organisms in response to biotic stimulus* | *0.040437* |
| GO:0000750 | pheromone-dependent signal transduction involved in conjugation with cellular fusion | 0.042559 |
| GO:0070785 | negative regulation of growth of unicellular organism as a thread of attached cells | 0.042559 |
| GO:0038032 | termination of G-protein coupled receptor signaling pathway | 0.042559 |
| GO:0051653 | spindle localization | 0.04893 |
| GO:0046777 | protein autophosphorylation | 0.04893 |
| GO:0034501 | protein localization to kinetochore | 0.04893 |
| *GO:0010506* | *regulation of autophagy* | *0.04893* |
| GO:0032220 | plasma membrane fusion involved in cytogamy | 0.04893 |
| *GO:0042127* | *regulation of cell proliferation* | *0.04893* |
| GO:0018343 | protein farnesylation | 0.04893 |
| GO:0006233 | dTDP biosynthetic process | 0.04893 |
| GO:0006296 | nucleotide-excision repair, DNA incision, 5'-to lesion | 0.04893 |
| GO:0007023 | post-chaperonin tubulin folding pathway | 0.04893 |
| GO:0042938 | dipeptide transport | 0.04893 |
| GO:0000183 | chromatin silencing at rDNA | 0.04893 |
| GO:0000746 | conjugation | 0.04893 |
| GO:0001319 | inheritance of oxidatively modified proteins involved in replicative cell aging | 0.04893 |
| GO:0000244 | spliceosomal tri-snRNP complex assembly | 0.04893 |
|  |  |  |
| ***FunCat categories enrichment "YL1 vs YQ2 - FBS 1h" (down)*** | |  |
| *FunCat ID* | *FunCat description* | *Exact p-value* |
| 10.03.02 | meiosis | 3.22E-06 |
| 20.01.01.07 | anion transport | 0.000388 |
| 20.01.03 | C-compound and carbohydrate transport | 0.000439 |
| 10.01.05.03.01 | meiotic recombination | 0.003094 |
| 38.01 | LTR retroelements (retroviral) | 0.003723 |
| 10.03.04.01 | centromere/kinetochore complex maturation | 0.004364 |
| 20.01.11 | amine / polyamine transport | 0.004814 |
| 32.07.03 | detoxification by modification | 0.009551 |
| 20.03.02.02 | symporter | 0.009582 |
| 01.03.16.03 | DNA degradation | 0.011837 |
| 10.03.04.09 | nuclear migration | 0.013129 |
| 1.07 | metabolism of vitamins, cofactors, and prosthetic groups | 0.013257 |
| 10.03.01 | mitotic cell cycle and cell cycle control | 0.014677 |
| 42.04.05 | microtubule cytoskeleton | 0.016653 |
| 14.07.03 | modification by phosphorylation, dephosphorylation, autophosphorylation | 0.016797 |
| 34.03.01 | synaptic transmission | 0.01733 |
| 10.03.01.02 | cell cycle arrest | 0.020699 |
| 20.03 | transport facilities | 0.021033 |
| *20.09.07* | *vesicular transport (Golgi network, etc.)* | *0.022999* |
| 43.01.03.09 | development of asco- basidio- or zygospore | 0.023283 |
| 20.03.02.03.01 | proton driven antiporter | 0.027734 |
| 40.10.02.03 | induction of apoptosis | 0.028484 |
| 32.01.01 | oxidative stress response | 0.030402 |
| 34.01.03 | homeostasis of anions | 0.03605 |
| 42.07 | endoplasmic reticulum | 0.03605 |
| 02.01.01 | glycolysis methylglyoxal bypass | 0.03605 |
| 10.01 | DNA processing | 0.040799 |
| 10.03.01.01 | mitotic cell cycle | 0.040978 |
| 30.05 | transmembrane signal transduction | 0.046396 |
| 20.01.26 | neurotransmitter transport | 0.048305 |
|  |  |  |
| ***KEGG pathways enrichment "YL1 vs YQ2 - FBS 1h" (down)*** | |  |
| *KEGG pathway ID* | *KEGG pathway name* | *Exact p-value* |
| *4140* | *Regulation of autophagy* | *0.029411* |
| 3420 | Nucleotide excision repair | 0.034942 |
| 3440 | Homologous recombination | 0.046394 |
|  |  |  |
| ***GO Biological process enrichment "YL1 vs YQ2 - FBS 1h" (up)*** | |  |
| *GO ID* | *GO name* | *Exact p-value* |
| GO:0045944 | positive regulation of transcription from RNA polymerase II promoter | 3.84E-05 |
| GO:0035690 | cellular response to drug | 0.000444 |
| GO:0006355 | regulation of transcription, DNA-templated | 0.000796 |
| GO:0006351 | transcription, DNA-templated | 0.000889 |
| GO:0070409 | carbamoyl phosphate biosynthetic process | 0.001378 |
| *GO:0008204* | *ergosterol metabolic process* | *0.001378* |
| GO:0016125 | sterol metabolic process | 0.001378 |
| GO:0006366 | transcription from RNA polymerase II promoter | 0.001442 |
| GO:0006796 | phosphate-containing compound metabolic process | 0.001592 |
| GO:1900443 | regulation of filamentous growth of a population of unicellular organisms in response to biotic stimulus | 0.002384 |
| GO:1900189 | positive regulation of cell adhesion involved in single-species biofilm formation | 0.00252 |
| GO:1900239 | regulation of phenotypic switching | 0.003028 |
| GO:0006543 | glutamine catabolic process | 0.004033 |
| *GO:0030447* | *filamentous growth* | *0.004454* |
| GO:0019740 | nitrogen utilization | 0.006717 |
| *GO:1900439* | *positive regulation of filamentous growth of a population of unicellular organisms in response to chemical stimulus* | *0.006776* |
| *GO:0010811* | *positive regulation of cell-substrate adhesion* | *0.012562* |
| GO:0070783 | growth of unicellular organism as a thread of attached cells | 0.012753 |
| GO:0070784 | regulation of growth of unicellular organism as a thread of attached cells | 0.012794 |
| GO:0034605 | cellular response to heat | 0.013208 |
| *GO:1900241* | *positive regulation of phenotypic switching* | *0.013647* |
| GO:0006470 | protein dephosphorylation | 0.0167 |
| GO:0044117 | growth of symbiont in host | 0.018929 |
| GO:0006506 | GPI anchor biosynthetic process | 0.019879 |
| GO:0016573 | histone acetylation | 0.025576 |
| GO:0070785 | negative regulation of growth of unicellular organism as a thread of attached cells | 0.025576 |
| *GO:0007155* | *cell adhesion* | *0.026879* |
| GO:0097502 | mannosylation | 0.027431 |
| GO:0044182 | filamentous growth of a population of unicellular organisms | 0.029228 |
| GO:0009405 | pathogenesis | 0.03549 |
| GO:0051131 | chaperone-mediated protein complex assembly | 0.037207 |
| GO:0006777 | Mo-molybdopterin cofactor biosynthetic process | 0.037207 |
| GO:0051592 | response to calcium ion | 0.037207 |
| GO:0043619 | regulation of transcription from RNA polymerase II promoter in response to oxidative stress | 0.037207 |
| GO:0045722 | positive regulation of gluconeogenesis | 0.037207 |
| *GO:0046513* | *ceramide biosynthetic process* | *0.037207* |
| GO:0034402 | recruitment of 3'-end processing factors to RNA polymerase II holoenzyme complex | 0.037207 |
| GO:0006113 | fermentation | 0.037207 |
| GO:0007010 | cytoskeleton organization | 0.037207 |
| GO:0019379 | sulfate assimilation, phosphoadenylyl sulfate reduction by phosphoadenylyl-sulfate reductase (thioredoxin) | 0.037207 |
| GO:0051693 | actin filament capping | 0.037207 |
| GO:0070407 | oxidation-dependent protein catabolic process | 0.037207 |
| GO:0097359 | UDP-glucosylation | 0.037207 |
| GO:0019359 | nicotinamide nucleotide biosynthetic process | 0.037207 |
| GO:0019310 | inositol catabolic process | 0.037207 |
| GO:0097043 | histone H3-K56 acetylation | 0.037207 |
| GO:0008298 | intracellular mRNA localization | 0.037207 |
| GO:0009166 | nucleotide catabolic process | 0.037207 |
| GO:0001897 | cytolysis by symbiont of host cells | 0.037207 |
| GO:0000288 | nuclear-transcribed mRNA catabolic process, deadenylation-dependent decay | 0.037207 |
| GO:0006928 | movement of cell or subcellular component | 0.037207 |
| GO:0031990 | mRNA export from nucleus in response to heat stress | 0.037207 |
| GO:0090296 | regulation of mitochondrial DNA replication | 0.037207 |
| GO:0006493 | protein O-linked glycosylation | 0.04175 |
| *GO:0043710* | *cell adhesion involved in multi-species biofilm formation* | *0.04175* |
|  |  |  |
| ***FunCat categories enrichment "YL1 vs YQ2 - FBS 1h" (up)*** | |  |
| *FunCat ID* | *FunCat description* | *Exact p-value* |
| *20.09.16.02* | *Type II protein secretion system (general secretory pathway, exocytosis)* | *0.001789* |
| *20.09.07.03* | *ER to Golgi transport* | *0.008902* |
| 18.01.01 | regulation by modification | 0.009677 |
| 16.13 | C-compound binding | 0.014585 |
| 11.02.03.01 | general transcription activities | 0.017429 |
| *30.01* | *cellular signalling* | *0.017536* |
| 40.01.05 | growth regulators / regulation of cell size | 0.021066 |
| 34.11.09 | temperature perception and response | 0.027116 |
| 14.07.02 | modification with sugar residues (e.g. glycosylation, deglycosylation) | 0.032666 |
| *40.01* | *cell growth / morphogenesis* | *0.039367* |
| 01.02.03.03 | assimilatory reduction of sulfur | 0.041952 |
| *20.09.07.05* | *intra Golgi transport* | *0.042051* |
| 1.04 | phosphate metabolism | 0.045681 |
| 20.09.07 | vesicular transport (Golgi network, etc.) | 0.047736 |
| 01.01.06.05 | metabolism of methionine | 0.049034 |
|  |  |  |
| ***KEGG pathways enrichment "YL1 vs YQ2 - FBS 1h" (up)*** | |  |
| *KEGG pathway ID* | *KEGG pathway name* | *Exact p-value* |
| *565* | *Ether lipid metabolism* | *0.024406* |
| 4113 | Meiosis - yeast | 0.034606 |
|  |  |  |
| ***GO Biological process enrichment "YL1 vs YQ2 + FBS 1h" (down)*** | |  |
| *GO ID* | *GO name* | *Exact p-value* |
| GO:0008150 | biological_process | 0.000299 |
| GO:0051321 | meiotic cell cycle | 0.004852 |
| GO:0009102 | biotin biosynthetic process | 0.004954 |
| GO:0044070 | regulation of anion transport | 0.009638 |
| GO:0006821 | chloride transport | 0.009638 |
| GO:0000226 | microtubule cytoskeleton organization | 0.009638 |
| GO:0051276 | chromosome organization | 0.040318 |
| GO:0034220 | ion transmembrane transport | 0.040318 |
| GO:0030010 | establishment of cell polarity | 0.040318 |
| GO:0032220 | plasma membrane fusion involved in cytogamy | 0.041284 |
| GO:0001919 | regulation of receptor recycling | 0.041284 |
| GO:0032008 | positive regulation of TOR signaling | 0.041284 |
| GO:0019441 | tryptophan catabolic process to kynurenine | 0.041284 |
| GO:0006296 | nucleotide-excision repair, DNA incision, 5'-to lesion | 0.041284 |
| GO:0006271 | DNA strand elongation involved in DNA replication | 0.041284 |
| GO:0032258 | CVT pathway | 0.041284 |
| GO:0007040 | lysosome organization | 0.041284 |
| GO:0051653 | spindle localization | 0.041284 |
| GO:0032439 | endosome localization | 0.041284 |
| GO:0044805 | late nucleophagy | 0.041284 |
| GO:0071230 | cellular response to amino acid stimulus | 0.041284 |
| GO:0034501 | protein localization to kinetochore | 0.041284 |
| GO:0034497 | protein localization to pre-autophagosomal structure | 0.041284 |
| GO:0000910 | cytokinesis | 0.041284 |
| GO:0006528 | asparagine metabolic process | 0.041284 |
| GO:0034727 | piecemeal microautophagy of nucleus | 0.041284 |
| GO:0042632 | cholesterol homeostasis | 0.041284 |
| GO:2000601 | positive regulation of Arp2/3 complex-mediated actin nucleation | 0.041284 |
| GO:0000746 | conjugation | 0.041284 |
|  |  |  |
| ***FunCat categories enrichment "YL1 vs YQ2 + FBS 1h" (down)*** | |  |
| FunCat ID | FunCat description | Exact p-value |
| *1.02* | *nitrogen, sulfur and selenium metabolism* | *0.003547* |
| 20.01.11 | amine / polyamine transport | 0.005105 |
| 40.01.05.01 | interpretation of external signals that control cell growth | 0.010756 |
| 30.05.02.24 | G-protein coupled receptor signalling pathway | 0.015003 |
| 10.03.02 | meiosis | 0.015093 |
| 01.01.03.01.02 | degradation of glutamine | 0.016048 |
| 42.04.05 | microtubule cytoskeleton | 0.017718 |
| 01.01.06.02.02 | degradation of asparagine | 0.018082 |
| 20.01.01.07.09 | chloride transport | 0.019031 |
| 11.02.03.04.01 | transcription activation | 0.024882 |
| 32.07 | detoxification | 0.025205 |
| 02.13.05 | regulation of respiration | 0.025629 |
| *42.19* | *peroxisome* | *0.03116* |
| 10.03.01.03 | cell cycle checkpoints (checkpoints of morphogenesis, DNA-damage,-replication, mitotic phase and spindle) | 0.032446 |
| 30.01.09.09 | fatty acid derivatives mediated signal transduction | 0.033012 |
| 34.01.03.05 | homeostasis of chloride | 0.033012 |
| 16.03.01 | DNA binding | 0.033739 |
| 1.2 | secondary metabolism | 0.036703 |
| *2.25* | *oxidation of fatty acids* | *0.039234* |
|  |  |  |
| ***KEGG pathways enrichment "YL1 vs YQ2 + FBS 1h" (down)*** | |  |
| *KEGG pathway ID* | *KEGG pathway name* | *Exact p-value* |
| 780 | Biotin metabolism | 0.004375 |
| 280 | Valine, leucine and isoleucine degradation | 0.007102 |
| 250 | *Alanine, aspartate and glutamate metabolism* | 0.032009 |
| 4111 | Cell cycle - yeast | 0.045643 |
|  |  |  |
| ***GO Biological process enrichment "YL1 vs YQ2 + FBS 1h" (up)*** | |  |
| *GO ID* | *GO name* | *Exact p-value* |
| GO:0006351 | transcription, DNA-templated | 0.004053 |
| GO:0006974 | cellular response to DNA damage stimulus | 0.009652 |
| GO:1900439 | positive regulation of filamentous growth of a population of unicellular organisms in response to chemical stimulus | 0.01231 |
| GO:0042254 | ribosome biogenesis | 0.016611 |
| GO:0008033 | tRNA processing | 0.019016 |
| GO:0008654 | phospholipid biosynthetic process | 0.021709 |
| GO:0032786 | positive regulation of DNA-templated transcription, elongation | 0.023445 |
| GO:0006383 | transcription from RNA polymerase III promoter | 0.023445 |
| GO:0035998 | 7,8-dihydroneopterin 3'-triphosphate biosynthetic process | 0.023445 |
| GO:0070125 | mitochondrial translational elongation | 0.023445 |
| GO:0071025 | RNA surveillance | 0.023445 |
| GO:0070481 | nuclear-transcribed mRNA catabolic process, non-stop decay | 0.023445 |
| GO:0070966 | nuclear-transcribed mRNA catabolic process, no-go decay | 0.023445 |
| GO:0097053 | L-kynurenine catabolic process | 0.023445 |
| GO:0046513 | ceramide biosynthetic process | 0.023445 |
| GO:0000379 | tRNA-type intron splice site recognition and cleavage | 0.023445 |
| GO:0006470 | protein dephosphorylation | 0.024453 |
| GO:0007067 | mitotic nuclear division | 0.029532 |
| GO:0007049 | cell cycle | 0.041561 |
| GO:0070526 | threonylcarbamoyladenosine biosynthetic process | 0.046345 |
| GO:0043419 | urea catabolic process | 0.046345 |
| GO:0009749 | response to glucose | 0.046345 |
|  |  |  |
| ***FunCat categories enrichment "YL1 vs YQ2 + FBS 1h" (up)*** | |  |
| *FunCat ID* | *FunCat description* | *Exact p-value* |
| 11.04.01 | rRNA processing | 0.004181 |
| 32.05.01.03 | chemical agent resistance | 0.004566 |
| 10.03.01.01.03 | G1/S transition of mitotic cell cycle | 0.038151 |
| *20.01.01.01.01.01* | *siderophore-iron transport* | *0.04119* |
| 20.01.01.07.08 | organic anion transport | 0.041323 |
| 01.01.06.06.01.03 | aminoadipic acid pathway | 0.043698 |
| 20.03.02.02 | symporter | 0.047074 |
|  |  |  |
| ***KEGG pathways enrichment "YL1 vs YQ2 + FBS 1h" (up)*** | |  |
| *KEGG pathway ID* | *KEGG pathway name* | *Exact p-value* |
| 3020 | RNA polymerase | 0.005417 |
|  |  |  |
| ***GO Biological process enrichment "YL1 vs YQ2 - FBS 24h" (down)*** | |  |
| *GO ID* | *GO name* | *Exact p-value* |
| GO:0008380 | RNA splicing | 0.002486 |
| GO:0000398 | mRNA splicing, via spliceosome | 0.00738 |
| GO:0006833 | water transport | 0.011043 |
| GO:0043103 | hypoxanthine salvage | 0.011043 |
| GO:0006146 | adenine catabolic process | 0.011043 |
| GO:0070544 | histone H3-K36 demethylation | 0.011043 |
| GO:0071497 | cellular response to freezing | 0.011043 |
| GO:0036164 | cell-abiotic substrate adhesion | 0.014678 |
| GO:0006397 | mRNA processing | 0.021369 |
| GO:0009168 | purine ribonucleoside monophosphate biosynthetic process | 0.021966 |
| GO:1902859 | propionyl-CoA catabolic process | 0.021966 |
| GO:0002666 | positive regulation of T cell tolerance induction | 0.021966 |
| GO:0005987 | sucrose catabolic process | 0.021966 |
| GO:0006573 | valine metabolic process | 0.021966 |
| GO:0008150 | biological_process | 0.026436 |
| GO:0036177 | filamentous growth of a population of unicellular organisms in response to pH | 0.02835 |
| GO:0071467 | cellular response to pH | 0.02835 |
| *GO:0007155* | *cell adhesion* | *0.030624* |
| GO:0006364 | rRNA processing | 0.033219 |
| GO:0007067 | mitotic nuclear division | 0.040264 |
| GO:0009117 | nucleotide metabolic process | 0.043457 |
|  |  |  |
| ***FunCat categories enrichment "YL1 vs YQ2 - FBS 24h" (down)*** | |  |
| *FunCat ID* | *FunCat description* | *Exact p-value* |
| 20.03.01 | channel / pore class transport | 0.006769 |
| 11.04.01 | rRNA processing | 0.014439 |
| 20.03.01.01 | ion channels | 0.016002 |
| 11.02 | RNA synthesis | 0.016587 |
| 01.01.11.02.02 | degradation of isoleucine | 0.039232 |
| 01.03.01.01 | purine nucleotide /nucleoside/nucleobase catabolism | 0.039232 |
| *42.19* | *peroxisome* | *0.042585* |
| 01.03.04.01 | pyrimidine nucleotide/nucleoside/nucleobase catabolism | 0.04403 |
| 40.01.05.01 | interpretation of external signals that control cell growth | 0.04403 |
| *1.01* | *amino acid metabolism* | *0.044476* |
| 20.01.25 | vitamine/cofactor transport | 0.046145 |
| 16.03.03 | RNA binding | 0.047185 |
| *2.25* | *oxidation of fatty acids* | *0.048577* |
| 01.01.11.03.02 | degradation of valine | 0.048803 |
| 11.04.03.01.10 | regulation of splicing | 0.048803 |
|  |  |  |
| ***KEGG pathways enrichment "YL1 vs YQ2 - FBS 24h" (down)*** | |  |
| *KEGG pathway ID* | *KEGG pathway name* | *Exact p-value* |
| 280 | Valine, leucine and isoleucine degradation | 0.005942 |
| 3040 | Spliceosome | 0.013388 |
|  |  |  |
| ***GO Biological process enrichment "YL1 vs YQ2 - FBS 24h" (up)*** | |  |
| *GO ID* | *GO name* | *Exact p-value* |
| GO:0045821 | positive regulation of glycolytic process | 0.000334 |
| GO:0045991 | carbon catabolite activation of transcription | 0.000334 |
| GO:1900233 | positive regulation of single-species biofilm formation on inanimate substrate | 0.001172 |
| GO:0006457 | protein folding | 0.001428 |
| GO:0015991 | ATP hydrolysis coupled proton transport | 0.001503 |
| GO:0006046 | N-acetylglucosamine catabolic process | 0.001954 |
| GO:0006043 | glucosamine catabolic process | 0.001954 |
| GO:0006511 | ubiquitin-dependent protein catabolic process | 0.002595 |
| *GO:0044182* | *filamentous growth of a population of unicellular organisms* | *0.002909* |
| GO:0007096 | regulation of exit from mitosis | 0.003218 |
| GO:0007035 | vacuolar acidification | 0.004769 |
| GO:0051701 | interaction with host | 0.005457 |
| GO:1900442 | positive regulation of filamentous growth of a population of unicellular organisms in response to neutral pH | 0.008146 |
| GO:0030010 | establishment of cell polarity | 0.00869 |
| *GO:0043709* | *cell adhesion involved in single-species biofilm formation* | *0.010302* |
| *GO:0009405* | *pathogenesis* | *0.011238* |
| GO:0044011 | single-species biofilm formation on inanimate substrate | 0.013054 |
| GO:0097316 | cellular response to N-acetyl-D-glucosamine | 0.013636 |
| *GO:0000032* | *cell wall mannoprotein biosynthetic process* | *0.016468* |
| *GO:0030447* | *filamentous growth* | *0.016789* |
| GO:0046476 | glycosylceramide biosynthetic process | 0.018349 |
| GO:0033615 | mitochondrial proton-transporting ATP synthase complex assembly | 0.018349 |
| GO:0042784 | active evasion of host immune response via regulation of host complement system | 0.018349 |
| GO:0006566 | threonine metabolic process | 0.018349 |
| GO:0034198 | cellular response to amino acid starvation | 0.018349 |
| GO:0007159 | leukocyte cell-cell adhesion | 0.018349 |
| GO:0008298 | intracellular mRNA localization | 0.018349 |
| GO:0015764 | N-acetylglucosamine transport | 0.018349 |
| GO:0051259 | protein oligomerization | 0.018349 |
| GO:0071456 | cellular response to hypoxia | 0.018349 |
| GO:0019318 | hexose metabolic process | 0.018349 |
| GO:0001897 | cytolysis by symbiont of host cells | 0.018349 |
| GO:1900445 | positive regulation of filamentous growth of a population of unicellular organisms in response to biotic stimulus | 0.01896 |
| GO:0009306 | protein secretion | 0.019527 |
| GO:0051603 | proteolysis involved in cellular protein catabolic process | 0.022805 |
| *GO:0008652* | *cellular amino acid biosynthetic process* | *0.029714* |
| GO:0032119 | sequestering of zinc ion | 0.036364 |
| GO:0016125 | sterol metabolic process | 0.036364 |
| GO:0006435 | threonyl-tRNA aminoacylation | 0.036364 |
| GO:0006616 | SRP-dependent cotranslational protein targeting to membrane, translocation | 0.036364 |
| GO:0045916 | negative regulation of complement activation | 0.036364 |
| GO:0045046 | protein import into peroxisome membrane | 0.036364 |
| GO:0006431 | methionyl-tRNA aminoacylation | 0.036364 |
| *GO:0008204* | *ergosterol metabolic process* | *0.036364* |
| GO:0007231 | osmosensory signaling pathway | 0.036364 |
| GO:1900188 | negative regulation of cell adhesion involved in single-species biofilm formation | 0.036364 |
| GO:0051453 | regulation of intracellular pH | 0.036364 |
| GO:0006357 | regulation of transcription from RNA polymerase II promoter | 0.037118 |
| GO:0006508 | proteolysis | 0.044632 |
| *GO:1900429* | *negative regulation of filamentous growth of a population of unicellular organisms* | *0.046223* |
|  |  |  |
| ***FunCat categories enrichment "YL1 vs YQ2 - FBS 24h" (up)*** | |  |
| *FunCat ID* | *FunCat description* | *Exact p-value* |
| 14.13.01.01 | proteasomal degradation (ubiquitin/proteasomal pathway) | 1.16E-05 |
| *20.09.07.03* | *ER to Golgi transport* | *0.000764* |
| 14.07.02.02 | N-directed glycosylation, deglycosylation | 0.000944 |
| 14.07.02.01 | O-directed glycosylation, deglycosylation | 0.002284 |
| 14.07.11 | protein processing (proteolytic) | 0.006577 |
| 14.1 | assembly of protein complexes | 0.010001 |
| 14.01 | protein folding and stabilization | 0.011042 |
| *01.01.13* | *regulation of amino acid metabolism* | *0.0163* |
| 41.01.01 | mating (fertilization) | 0.020939 |
| *20.09.16* | *cellular export and secretion* | *0.022796* |
| 32.01 | stress response | 0.023432 |
| 18.01.07 | regulation by binding / dissociation | 0.023797 |
| 40.01.03 | directional cell growth (morphogenesis) | 0.028852 |
| 16.01 | protein binding | 0.03153 |
| 10.03.05 | cell cycle dependent cytoskeleton reorganization | 0.032566 |
| 01.25.01 | extracellular polysaccharide degradation | 0.032566 |
| 20.09.05 | non-vesicular ER transport | 0.034992 |
| 14.07.02 | modification with sugar residues (e.g. glycosylation, deglycosylation) | 0.035319 |
| 18.02.01.02 | enzyme inhibitor | 0.03991 |
| 11.06.03 | mRNA modification | 0.043186 |
|  |  |  |
| ***KEGG pathways enrichment "YL1 vs YQ2 - FBS 24h" (up)*** | |  |
| *KEGG pathway ID* | *KEGG pathway name* | *Exact p-value* |
| 3050 | Proteasome | 0.000217 |
| 4141 | Protein processing in endoplasmic reticulum | 0.001568 |
| 4145 | Phagosome | 0.006763 |
|  |  |  |
| ***GO Biological process enrichment "YL1 vs YQ2 + FBS 24h" (down)*** | |  |
|  |  |  |
| *GO ID* | *GO name* | *Exact p-value* |
| GO:0015031 | protein transport | 5.57E-05 |
| *GO:0006412* | *translation* | *0.000173* |
| *GO:0016192* | *vesicle-mediated transport* | *0.000284* |
| GO:0009116 | nucleoside metabolic process | 0.000634 |
| GO:0006886 | intracellular protein transport | 0.000862 |
| GO:0006810 | transport | 0.000876 |
| GO:0045048 | protein insertion into ER membrane | 0.001589 |
| *GO:0006166* | *purine ribonucleoside salvage* | *0.001589* |
| GO:0035435 | phosphate ion transmembrane transport | 0.003129 |
| GO:0006817 | phosphate ion transport | 0.003129 |
| GO:0035690 | cellular response to drug | 0.003728 |
| *GO:0006888* | *ER to Golgi vesicle-mediated transport* | *0.006604* |
| GO:0006406 | mRNA export from nucleus | 0.010461 |
| GO:0006038 | *cell wall chitin biosynthetic process* | 0.010461 |
| *GO:0006417* | *regulation of translation* | *0.010461* |
| GO:0006189 | 'de novo' IMP biosynthetic process | 0.013736 |
| GO:0006487 | protein N-linked glycosylation | 0.017394 |
| GO:0071432 | peptide mating pheromone maturation involved in conjugation with cellular fusion | 0.023276 |
| GO:0034969 | histone arginine methylation | 0.023276 |
| *GO:0006896* | *Golgi to vacuole transport* | *0.023276* |
| GO:0071586 | CAAX-box protein processing | 0.023276 |
| GO:0018195 | peptidyl-arginine modification | 0.023276 |
| GO:0019307 | mannose biosynthetic process | 0.023276 |
| GO:0019919 | peptidyl-arginine methylation, to asymmetrical-dimethyl arginine | 0.023276 |
| GO:0018279 | protein N-linked glycosylation via asparagine | 0.023276 |
| GO:0071816 | tail-anchored membrane protein insertion into ER membrane | 0.023276 |
| GO:0017148 | negative regulation of translation | 0.023276 |
| *GO:0006168* | *adenine salvage* | *0.023276* |
| GO:0016051 | carbohydrate biosynthetic process | 0.023276 |
| GO:0051223 | regulation of protein transport | 0.023276 |
| GO:0044209 | AMP salvage | 0.023276 |
| GO:0043985 | histone H4-R3 methylation | 0.023276 |
| GO:0006042 | glucosamine biosynthetic process | 0.023276 |
| GO:0061077 | chaperone-mediated protein folding | 0.023276 |
| GO:0046836 | glycolipid transport | 0.023276 |
| GO:0070988 | demethylation | 0.023276 |
| GO:0035247 | peptidyl-arginine omega-N-methylation | 0.023276 |
| GO:0051603 | proteolysis involved in cellular protein catabolic process | 0.035468 |
| GO:0006696 | ergosterol biosynthetic process | 0.040759 |
| GO:0006486 | protein glycosylation | 0.043665 |
| GO:0006525 | arginine metabolic process | 0.046013 |
| GO:0035246 | peptidyl-arginine N-methylation | 0.046013 |
| GO:0010430 | fatty acid omega-oxidation | 0.046013 |
| GO:0006167 | AMP biosynthetic process | 0.046013 |
| GO:0048252 | lauric acid metabolic process | 0.046013 |
| GO:0006428 | isoleucyl-tRNA aminoacylation | 0.046013 |
| GO:0007323 | peptide pheromone maturation | 0.046013 |
| GO:0007266 | Rho protein signal transduction | 0.046013 |
|  |  |  |
| ***FunCat categories enrichment "YL1 vs YQ2 + FBS 24h" (down)*** | |  |
| *FunCat ID* | *FunCat description* | *Exact p-value* |
| *12.04* | *translation* | *9.42E-05* |
| 12.01.01 | ribosomal proteins | 0.000278 |
| 12.01 | ribosome biogenesis | 0.000543 |
| *01.03.01* | *purin nucleotide/nucleoside/nucleobase metabolism* | *0.000586* |
| *20.09.07.03* | *ER to Golgi transport* | *0.003769* |
| 14.07.02.02 | N-directed glycosylation, deglycosylation | 0.003864 |
| 01.05.02 | sugar, glucoside, polyol and carboxylate metabolism | 0.004652 |
| 01.03.01.03 | purine nucleotide/nucleoside/nucleobase anabolism | 0.01286 |
| 14.07.02 | modification with sugar residues (e.g. glycosylation, deglycosylation) | 0.023698 |
| 14.07 | protein modification | 0.024351 |
| 34.01.03.03 | homeostasis of phosphate | 0.027292 |
| 01.05.03.03.04 | chitin anabolism | 0.027292 |
| 01.05.03.03 | chitin metabolism | 0.028724 |
| 01.05.03.03.07 | chitin catabolism | 0.028724 |
| 01.05.02.01 | nucleotide-sugar metabolism | 0.033264 |
| 16.05 | polysaccharide binding | 0.042777 |
| 10.03.05 | cell cycle dependent cytoskeleton reorganization | 0.042777 |
| 01.05.09.04 | aminosaccharide anabolism | 0.046553 |
|  |  |  |
| ***KEGG pathways enrichment "YL1 vs YQ2 + FBS 24h" (down)*** | |  |
| *KEGG pathway ID* | *KEGG pathway name* | *Exact p-value* |
| 3010 | Ribosome | 0.000365 |
| 4141 | Protein processing in endoplasmic reticulum | 0.002877 |
| 513 | Various types of N-glycan biosynthesis | 0.015773 |
| 510 | N-Glycan biosynthesis | 0.016893 |
|  |  |  |
| ***GO Biological process enrichment "YL1 vs YQ2 + FBS 24h" (up)*** | |  |
| *GO ID* | *GO name* | *Exact p-value* |
| GO:0070370 | cellular heat acclimation | 0.000814 |
| *GO:0006075* | *(1->3)-beta-D-glucan biosynthetic process* | *0.007587* |
| GO:0000076 | DNA replication checkpoint | 0.01665 |
| GO:0015718 | monocarboxylic acid transport | 0.01665 |
| GO:0015727 | lactate transport | 0.01665 |
| GO:0006021 | inositol biosynthetic process | 0.01665 |
| GO:0006833 | water transport | 0.01665 |
| GO:0016598 | protein arginylation | 0.01665 |
| GO:0071497 | cellular response to freezing | 0.01665 |
| GO:0036086 | positive regulation of transcription from RNA polymerase II promoter in response to iron ion starvation | 0.01665 |
| GO:0035873 | lactate transmembrane transport | 0.01665 |
| GO:0070414 | trehalose metabolism in response to heat stress | 0.01665 |
| *GO:0007155* | *cell adhesion* | *0.019301* |
| GO:0071897 | DNA biosynthetic process | 0.025023 |
| GO:0006573 | valine metabolic process | 0.033025 |
| GO:1902859 | propionyl-CoA catabolic process | 0.033025 |
| GO:0006435 | threonyl-tRNA aminoacylation | 0.033025 |
| GO:0051085 | chaperone mediated protein folding requiring cofactor | 0.033025 |
| GO:0009186 | deoxyribonucleoside diphosphate metabolic process | 0.033025 |
| GO:0007329 | positive regulation of transcription from RNA polymerase II promoter by pheromones | 0.033025 |
| GO:0007109 | cytokinesis, completion of separation | 0.033025 |
| GO:0006260 | DNA replication | 0.04753 |
| GO:0007039 | protein catabolic process in the vacuole | 0.04913 |
| GO:0006084 | acetyl-CoA metabolic process | 0.04913 |
| *GO:0042546* | *cell wall biogenesis* | *0.04913* |
|  |  |  |
| ***FunCat categories enrichment "YL1 vs YQ2 + FBS 24h" (up)*** | *FunCat description* | *Exact p-value* |
| *FunCat ID* | peroxisome | 0.001732 |
| 42.19 | C-compound and carbohydrate transport | 0.006594 |
| 20.01.03 | C-compound and carbohydrate metabolism | 0.007936 |
| 1.05 | regulation of C-compound and carbohydrate metabolism | 0.010255 |
| 01.05.25 | *lipid/fatty acid transport* | *0.011575* |
| *20.01.13* | metabolism of secondary monosaccharides | 0.015652 |
| 01.20.01.01 | proton driven symporter | 0.018793 |
| 20.03.02.02.01 | DNA synthesis and replication | 0.020726 |
| 10.01.03 | metabolism of nonprotein amino acids | 0.0259 |
| 01.20.17.01 | *fatty acid metabolism* | *0.028649* |
| *01.06.05* | general transcription activities | 0.037924 |
| 11.02.03.01 | peptide transport | 0.040939 |
| 20.01.09 | metabolism of alkaloids | 0.046236 |
| 01.20.17.09 |  |  |
|  |  |  |
| ***KEGG pathways enrichment "YL1 vs YQ2 + FBS 24h" (up)*** | |  |
| *No significant category as significance level* | |  |
